# Wild food plants of popular use in Sicily

**DOI:** 10.1186/1746-4269-3-15

**Published:** 2007-03-30

**Authors:** Francesca Lentini, Francesca Venza

**Affiliations:** 1 Department of Botanical Sciences, University of Palermo, Palermo. Italy

## Abstract

In the present work the authors report the result of their food ethnobotanical researches, which have been carried out in Sicily during the last thirty years. Data concerning 188 wild species used in the traditional Sicilian cuisine are reported. The authors underline those species that are partially or completely unknown for their culinary use and they illustrate other species that local inhabitants suggested in the prevention or treatment of symptomatologies caused by a refined diet, poor in vegetables. These data want to contribute to avoid the loss of traditional knowledge on uses and recipes concerning wild food botanicals, and to encourage further studies for those species that have not yet been sufficiently researched in their food chemical and nutritional profile. These studies may also suggest new applications for a few botanicals in medico-nutritional fields. The work includes also a short review of the seaweeds and mushrooms traditionally gathered and consumed in Sicily.

## Background

Numerous scientific researches conducted in the last few years have revealed that a diet rich in fibre, complex carbohydrates, vitamins and mineral salts, is the diet considered most ideal in order to maintain good health and prevent various illnesses. Fruit and vegetables are the food that contains a large quantity of vitamins and it is mainly because of this that many researchers have focused their attention on the studies of nutritional plants.

Many species have already been examined concerning this aspect but there are nevertheless many more which merit examination. Ethnobotany is a preliminary method of research, suitable for gathering information on the nutritional use of plants. It has been proven, time and time again, that the 'quack' medical knowledge handed down by the common people constitutes sources of information useful for scientific research and that many plants utilised exclusively in popular tradition, when exposed under scientific examination, have been found to be useful for different sectors in the industry [1]. Therefore, science and tradition have a strong connection between them; science, in fact, has often traditional origins.

Considering the fact that ethnobotany mainly concentrates on the individuation of plants with an applicative purpose, the authors, that have shown an interest in the argument for a long time, have completed a research conducted on the plants used as food and/or for aromatic purposes in popular, Sicilian tradition and have referred to, in this contribution, the results obtained.

One of the main objectives of this research is to individuate, amongst the plants of the Sicilian flora, those more or less known for their nutritional use and to provide suggestions on how to embark upon researches in the medical-nutritional field. In fact, many nutritional plants are also utilised for medical purposes and are often advised as a remedy in order to stabilise alternative functions of the human organism or simply to purify or cure some trivial pathologies.

To consider food as medicine is part of a culture and a millennial human practice, in fact, ancient documents, testify the consummation of many plants in order to prevent numerous illnesses. In the Ebers papyrus (around 1550 B.C.) barley, figs, olives, garlic and onions were mentioned amongst the plants of therapeutic use and today, more advanced scientific research reveals that human health is directly connected to nutrition.

This research aims to verify to what extent can popular tradition consent to the validation of the uses suggested by common people and to revaluate the consumption of the local species of flora by suggesting their integration as medicinal food in alimentary diet.

## Methods of study

**Figure 1:**
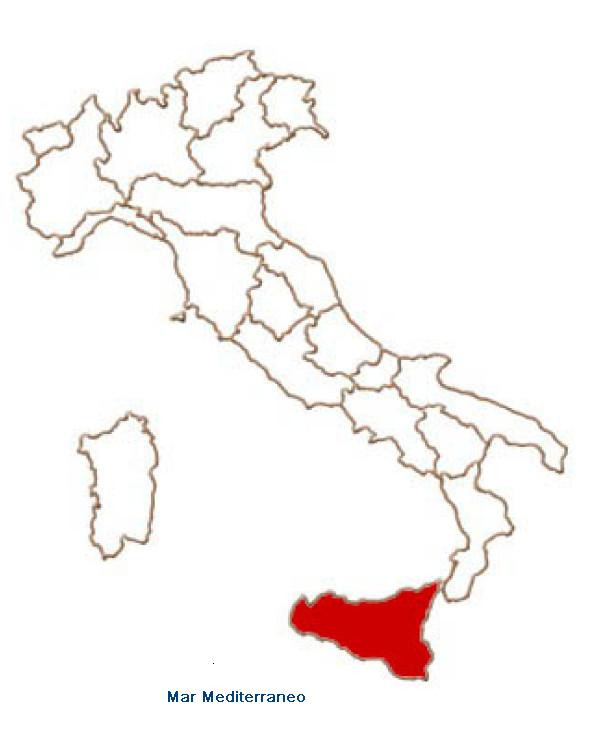
Map of research area.

The studies have been conducted in Sicily (Fig. 1), an island with a surface of 25,707 square km, situated in the middle of the Mediterranean sea and is divided from Italy (the country to which it belongs) by a narrow section of water (Messina strait). Due to its unique position, vast variety of mountains and substrates and its mild climate, the island is rich in species.

According to Di Martino and Raimondo [2] its vascular flora consists of 2361 taxa. This number has grown because many more taxa have been added, today in fact almost 3000 taxa can be counted, including those in the surrounding islands [3]. Due to its richness in species, Sicily is considered as one of the territories with the higthest vegetal biodiversity in the whole of the Mediterranean area. As for food plants of traditional use which are the subject of this research, the information has been collected by working on researches carried out in the island's rural communities and in particularly in the provinces of Agrigento, Caltanissetta, Enna, Messina, Palermo, Ragusa, Siracusa, Trapani, and in the Eolic islands (Messina), Egadi Islands and Pantelleria (Trapani), Pelagie islands (Agrigento) and Ustica (Palermo) which, in the past, have contributed to a lot of useful information concerning other researches [4-18]. Nevertheless, where the popular flora of Catania is concerned, the authors have referred exclusively to bibliographical researches instead [19-23]. This study has been carried out in two stages: during the first stage the cooperation of the local people, above all shepherds, farmers and old housewives has been indispensable and has provided valuable information about the plants of popular use, such as the vernacular name, parts used, period of collection and the receipts used in order to prepare traditional dishes; furthermore they also contributed in the collection of the plants. The second stage has been carried out in the Department of Botanical Sciences of the University of Palermo, where the collected species have been dehydrated and taxonomically determined using the analytical keys. The obtained exiccata have been kept in the Ethnobotanical Herbarium in the same Department. The study has been completed through an accurate bibliographical research which has allowed comparing the information collected in Sicily with the one known from literature [24-61].

The data reported has been shortened and summarised and placed in a scheme (Additional file 1) in which for every species the scientific, names, family, Italian vernacular names, used parts, traditional receipts and also the food uses recorded outside Sicily with the corresponding bibliographical references have been provided. In the scheme the species marked with the symbol @ are quoted as edible in the database PFAF [24]. The nomenclature used is the same adopted by Pignatti in Flora d'Italia [60] apart from the following families *Apiaceae*, *Asteraceae*, *Brassicaceae*, *Fabaceae*, *Lamiaceae *and *Smilacaceae *for which the authors referred to Jude et al., 1999 [61].

Furthermore, in Additional file 2, the scientific names and the corresponding Sicilian vernacular names are reported and, in Table 1, some traditional Sicilian vernacular culinary terms have been cited in the Additional file 1.

**Table 1:** Explanations of the Sicilian vernacular culinary terms used in Additional file 1

**Alivi acciurate**: Black olives cooked on the oven with salt and seasoned with *Rosmarinus officinalis*.
**Alivi cunsate**: Green olives seasoned with pounded garlic, salt, oil, celery, carrots and oregano.
**Babaluceddi**: Little white snails, boiled in water and seasoned whit typical sauces made whit parsley, tomatoes, garlic, pepper, salt and olive oil
**Cotognata**: Typical sweets for the winter made with fruits of *Cynodon oblonga *cooked with sugar, rind of ripe lemon, cinnamon, passed through the sieve and then dried in moulds for sweets.
**Cous cous**: Typical dish of the traditional cuisine of Trapani Province prepared with drench of wheat mixed (*n'cucciata*) with oil and salt in a soup tureen called "*mafaradda*" and then cooked with stem in a typical earthenware called "*kuskusiera*" with laurel leaves, onions and seasoned with a "pesto" of almonds, parsley, oil and a fish soups, meat soup or vegetables soup.
**Cubaida o quadrelli**: Pastry of almonds, hazelnuts and pistachios, coated with burnt sugar.
**Cuccia**: Typical dish eaten in every part of Sicily during the festivity in homage of Saint Lucy (13 December) It is prepared cooking wheat, chick-peas, orange's rinds and 8 broad beans whit the stem. After a night this soup can be seasoned with ricotta and sugar or with cooked and sugared must of grapefruit.
**Cuddiruni**: It is a typical kind of pizza of the province of Messina made with wheat flour, water, salt, oil yeast and filled with boiled leaves of *Beta vulgaris*, potatoes, and onions
**Focacce**: Leavened bread seasoned with vegetables, spices, oil and salt.
**Frittata o frocia**: Omelette with cheese and browned vegetables.
**Frutta di Marturana**: Almond paste mixed with sugar, water and 3 drops for kg of essential oil of clove, shaped and painted with vegetal dyes to represent different types of Sicilian fruits, it is traditionally eaten during the festivity of All Saints (1 November).
**Maccu ri favi e finocchiu**: Soup made with broad beans, onion, tomatoes, chilly, boiled leaves of *Foeniculum vulgare *subsp. *vulgare *seasoned with oil and eaten with toasted bread.
**Mostarda di ficurinia**: Typical pastries prepared with juice of *Opuntia ficus indica *boiled and mixed with drench of wheat, almonds, hazelnuts, cinnamon and sugar.
**Mostarda di vinu cottu**: Cooked wine boiled with wheat flour and sugar.
**Mustazzoli di vinu cottu**: Typical pastries prepared with flour, sugar, cooked must, and aniseeds.
**Nieputiddata**: Typical dish of the Eolian Islands prepared by mixing the plant with garlic and cooking it with oil, tomatoes, adding eggs, some other vegetables and stale bread.
**Paparotta**: Soup made with *Borago officinalis *leaves and semolina and seasoned with salt and olive oil.
**Pizichintì**: Mostarda di ficurinia cut in very small pieces dusted with sugar.
**Purpette cu sucu**: Meat balls seasoned with *Leontodon tuberosus*, fried in bowling olive oil and seasoned with tomato sauce.
**Purpette di finucchieddru cu sucu**: Boiled leaves of *Foeniculum vulgare *subsp. *vulgare *well minced, and mixed with eggs, cheese, pine-seeds and raisin and shaped as vegetable rissoles then fried in bowling olive oil and seasoned with tomato sauce.
**Rianata**: Typical pizza eaten in Trapani seasoned with oil, tomatos, con olio, salt, anchovies, pecorino cheese and origanum.
**Salmoriglio**: Typical seasoning for meat, fish and vegetables prepared with oil, olio, origanum, laurel leaves, pepper and parsley.
**Stemperata**: Typical dish prepared with capers, olives, garlic, oil, broccoli, chilli peppers, carrots and celery.
**Sucu cu a mazzaredda**: Sauce for pasta prepared with leaves of *Rapistrum rugosum*, tomato sauces, garlic, olive oil and pecorino cheese.
**Sucu cu a sarvia sarvaggia**: Sauce for pasta prepared browning the leaves of *Phlomis fruticosa *in bacon fat, butter or olive oil.
**Sucu cu l'agghia a la trapanisa**: Sauce for pasta prepared with oil, garlic, almonds, tomatoes, parsley, basil and pecorino cheese.
**Sucu da pasta cu li sardi**: Sauce for pasta prepared with boiled leaves and seeds of *Foeniculum vulgare *browned in olive oil, onion saffron, sardines, raisin, pine-seeds.
**Sucu da pasta cu lu tonnu**: Sauce for pasta prepared with onion, garlic, black and green olives, oil, tomatoes, capers and tuna fish.
**Sucu di sparaci di scupazzu impurrazzati**: A traditional dish of Palermo, prepared by wrapping the "sparaci" in the "purrazzu" leaves (*Asphodelus microcarpus*) and by cooking them in tomato sauce.
**Sucu pa pasta cu li ficurinnia**: Sauce for pasta prepared with olive oil, garlic and pulp of the fruits of *Opuntia ficus indica *without seeds.
**Tagliatelle verdi**: Hand made pasta prepared whit wheat flour water, eggs and leaves of different vegetables.
**Vinu cottu**: Dense liquid obtained cooking must of grapefruits and sugar for at least 1 hour.
**Viscotta ri ficu**, **buccellati**: Pastry cookies filling of figs jam, cinnamon, almonds and comfits.
**Vucciddrati**: Leavened bread seasoned with oil, salt, rosemary and cooked in oven during the festivity of Saint Joseph (9th March).

This work has been expanded with data taken from Battiato [62] concerning edible seaweeds of traditional use and data concerning edible mushrooms in Sicily taken from [63][64][65]: this data is reported in a table (Table 2).

**Table 2:** Review of the mushrooms traditionally gathered and consumed in Sicily

**Scientific name**	**Family and Italian vernacular name**	**Sicilian vernacular name**	**Traditional recipes in Sicily**
*Agaricus arvensis *Scheff.:Fr.	Agaricaceae	Funcia campagnola, funcia picurina (63)	It is eaten raw or cooked (63)
*Agaricus campester *L.:FR.	Agaricaceae	Funcia campagnola, funcia picurina (63)	It is eaten raw or cooked (63)
*Agrocybe cylindracea *(DC.:Fr.)Maire	Bolbitiaceae	Funcia di chiuppo, funcia di Cacciamo (63)	The caps it is cooked in risottos added to the sauce or preserved in vinegar (63)
*Amanita cesarea *(Scop.:FR.)Pers.	*Amanitaceae *Ovolo buono	Funcia d'ovu (63)	It is eaten raw in salads with olive oil, pepper and salt (63)
*Amanita vaginata *(Bull.:Fr.) Lam.f.vaginata	Amanitaceae	Funci palummi (63)	It is eaten but can be responsible for gastro-intestinal disorders (63)
*Armillaria mellea *(Vahl.:Fr.) Kumm	*Tricholomataceae *Chiodino	Funcia di chiuppu, funcia di ceusu, funcia di salici, funcia di urmu, funcia di speziu, funcia di aranciu, funcia di pirali, funci di mennula (64)	It is preserved in olive oil (63). It is eaten only well cooked (64)
*Armillaria tabescens *(Scop.:Fr.) Emel.	*Tricholomataceae *Chiodino senza anello	Funcia i traversa (63).	It is preserved in olive oil (63).
(Pers.) Morgan	Astraeaceae	Piditu di lupu a stidda (63).	It is edible (63)
*Boletus aereus *Bull.: Fr.	*Boletaceae *Bronzino, porcino Nero	Purcini (63) Porcinu niuru, funciu siddu, testa niura (64)	It is eaten raw in salads or cooked in many recipes, it is also suitable for freezing and as alimentary preserves (63). It is edibleand it is consumed from the spring to the autumn (2)
*Boletus aestivalis *(Paulet) Fr.	*Boletaceae *Porcino estivo	Funciu di istati (64)	It is edible and it is consumed from the spring to the autumn (64)
*Boletus badius *(Fr.:Fr.) Fr	Boletaceae	Funcia castagnara (63)	It is edible (63)
*Boletus chrysenteron *Bull.S.str.	Boletaceae	Funci 'i filici (63)	It is edible (63)
*Boletus edulis *Bull.:Fr.s.str.	Boletaceae	Purcini (63) Funciu siddu, testa di fagu (64)	It is eaten raw in salads or cooked in many recipes, it is also suitable for freezing and as alimentary preserves (63). It is consumed from the summer to the autumn (64)
*Boletus impolidus *Fr.	*Boletaceae *Boleto a cappello granuloso	Funciu d'ogghiu (64)	It is consumed from the summer to the autumn (64).
*Boletus luridus *Schaeff.	Boletaceae	Funciu vilinusu, musso di voi (64)	It is eaten after well cooked (64).
*Boletus reticulatus *Scheff.	Boletaceae	Purcini (64)	It is eaten raw in salads or cooked in many recipes; it is also suitable for freezing and as alimentary preserves (64).
*Boletus rhodoxanthus *(Krombth.) Kallenb.	Boletaceae	Funcia lardara (64).	It is eaten well cooked (64).
*Cantharellus cibarius *F.:Fr.	*Cantharellaceae *Galletto, gallinaccio	Funcia spizzera cricchia di jadu Cricchia di jaddu, iadduzzu, ciurittu (64)	It is consumed from the spring to the autumn (63,64).
*Cantharellus cinereus *Pers.: Fr.	Cantharellaceae	Trombette dei morti (63)	It is dried, ground and used as a substitute for pepper or it is cooked with others fungi as a sauce for pasta and rice (63)
*Cratarellus *cornucopioides (Linn.) Pers.	*Cantharellaceae*	Trombetta dei morti (63)	It is dried, ground and used as a substitute for pepper or it is cooked with others fungi as a sauce for pasta and rice (63)
*Clytocibe geotropa *(Lam&DC.) Quél.	*Tricholomataceae *Chiodino	Funci i rota, funci filera (63)	It is used cooked with oil and garlic to flavour tomato sauce, pasta or meat sauces (63)
*Clytocibe gibba *(Pers.:Fr.) Kumm	Tricholomataceae	Funcia campagnuledda (63)	It is cooked with oil and garlic to flavour tomato sauce for pasta or meat sauces (63)
*Clytocibe nebularis *(Batsch:fr.) Kumm.	Tricholomataceae	Funcia di pampina (63)	It is eaten but can give allergic reactions (63)
*Clytocibe odora *(Bull.:Fr.) Kumm.	*Tricholomataceae *Fungo dell'anice, agarico anisato	Funciu d'anici (64)	This mushroom gives a pleasant and intense smell of aniseeds to dishes or it is mixed with others fungi to improve their taste (63). It is eaten in soup with others mushrooms, onion, oil, bread during the autumn (64)
*Coprinus comatus *(Müll.:fr) Pers.	*Coprinaceae *Agarico chiomato, fungo dell'inchiostro, coprino chiomato	Calamaru calamaricchiu (63). Funciu calamaru (64)	It is edible (63) and eaten from the spring to the autumn (64)
*Chroogomphus rutilus *(Sch.:Fr) O.K.Miller.	*Gomphidaceae *Chiodetto	Funciu vavusu, funciu viscidu di zappino (64).	It is edible and consumed from the summer to the autumn (64)
*Fistulina hepatica *(Bull.:Fr.)	Fistulinacae Lingua di bue	Lingua di bue (64).	It is eaten in salads added to tomato sauce or preserved in olive oil (63)
*Grifola frondosa *(Dicks.: Fr.) S.F. Gray	Polyporaceae	Funci di jaddu (63) Rinasca (65)	The young mushroom is ebible (1) and is preserved in olive oil or in winegar (65)
*Gyroporus castaneus *(Bull. :Fries) Quél.	*Boletaceae *Boleto castano, fungo del castagno	Funcia di li vigni (63). Funci di castagna, funciu di cannilla (64)	It is edible (63) and consumed from the summer to the autumn (64)
*Helvella crispa *(Scoop.) Fr.	*Helvellaceae *Spugnola crespa	Spugnola crespa (63), funci di chiddi rizzi, munachessi (64)	It is edible (63) and consumed in autumn (64)
*Hericium coralloides *(Scop.:Fr.) Pers.	Hydnaceae	Funcia varvavitrana masculina (63)	It is eaten fried in olive oil (63)
*Hericium erinaceus *(Bull.:Fr.) Pers.	Hydnaceae	Varvazzi, varva di vecchiu, varvavitranu (63)	It is edible (63)
*Hydnum repandum *L.:Fr.	*Hydnaceae *Steccherino dorato	Funciu musca, funci di jaddu (64)	It is edible and consumed from the summer to the autumn (64)
Hydnum *rufescens *Sch.:Fr.	*Boletaceae *Steccherino dorato	Funciu musca, funci di jaddu (64)	It is edible and consumed from the summer to the autumn (64)
*Hypsizygus ulmarius *(Bull:Fr.) Redhead	Tricholomataceae	Funcia d'urmu (63)	It is edible (63)
*Lactarius deliciosus *(L.:Fr.) S.F. Gray	*Russulaceae *Sanguinaccio, Sanguinello, Rossella	Lattario (64)	It is cooked in tomato sauce as condiment for pasta or roasted on the barbecue (63). It is edible and consumed from the summer to the first part of winter (64)
*Lactarius piperatus *(L.:Fr.) Pers.	Russulaceae	Funcia lataria, funcia pipirita (63)	This mushroom is difficult to digest, but it is eaten by a few people who appreciate its bitter taste (63)
*Laetiporus sulphureus *(Bull.) Murrill	*Polyporacae *Poliporo sulfureo	Funciu di carrubbu (63)	It is eaten in Ragusa province sliced and fried in olive oil (63)
*Langermannia gigantea *(Batsch:Pers.) Rostk.	Lycoperdaceae	Bissinu, fissinu, sbissinu, ricuttuni (63)	It is edible (63)
*Leccinum aurantiacum *(Bull.:Fr.) S.F.Gray.	*Boletaceae *Porcinello rosso	Funciu d'arbaneddu, porcineddu russu (64)	It is edible and consumed from the summer to the autumn (64)
*Leccinum corsicum *(Roll.) Sing.	*Boletaceae *porcinello del cisto	Funcia di rusedda, funci ebrei (64)	The caps, well cooked, are stuffed with chopped garlic, onion, olive oil, salt, pepper and anchioves and recoated with bread-crumbs and pecorino cheese (63)
*Leccinum lepidum *(Bouchet ex Essette) Quadr.	*Boletaceae *Leccino porcinello, della sabbia	Funcia arancini (63). Liccinu, funci i vacca (64)	It is edible (63) and consumed in autumn (64)
*Leccinum scabrum *(Bull.:Fr.) S.F.Gray	*Boletaceae *Porcinello grigio	Funciu di vitudda (64)	It is edible and consumed from the summer to the autumn (64)
*Lepista nuda *(Bull. : Fr.) Cooke	*Tricholomataceae *Agarico violetto	Funcia di latticuognu (63)	It is finely sliced and cooked in oil and garlic to be served with other fungi as an accompaniment to meat or a sauce for pasta (63)
*Lycoperdon echinatum *Pers.:Pers.	*Lycoperdaceae *Vescia, vescia echinata	Funci ri tabaccu, funci tabbaccari (64)	It is edible and consumed from the summer to the autumn (64)
*Lycoperdon perlatum *Pers:Pers	Lycoperdaceae	Piditu di lupi (63).	It is edible (63)
Schaeff.:Pers.	*Lycoperdaceae *Vescia	Piditu di lupi (63)	It is edible (63)
*Macrolepiota excoriata *(Schaeff.:Fr.) Wasser	*Agaricaceae *Bubbola buona, bubbola di prato	Funcia picurina (63)	It is edible (63)
*Macrolepiota procera *(Scop. Fr.) Sing.	*Agaricaceae *Mazza di tamburo	Funciu cappidinu (63)	The large caps can be coated with bread-crumbs then cooked under the grill while the stipe can be dried, finely chopped and used as a seasoning in cooking (63)
*Marasmius oreades *(Bolt:Fr.)Fr.	*Marasmiaceae *Gambe secche, chiodini dei prati		it is use as flavouring (63)
*Morchella esculenta *(L.:Fr.) Pers.	*Morchellaceae *s Spugnola gialla	Sponsi, cugni di vecchia, ventripecura, funci ventri di pecura (64)	It is edible (63). but only well cooked (64)
*Morchella conica *Pers. var.*costata *Ventenat	*Morchellaceae *Spugnola bruna	Ventripecura, funci ventri di pecora (64,65)	It is edible but only well cooked (64,65)
*Pisolithus arhizus *(Scop.: Pers.) Rauschert	*Pisolithaceae *Pisello di pietra	Catatunfuli, falsi tartufi (63)	It is edible (63)
*Pleurotus eryngii *(DC.:Fr.) Quel. var. *eryngii*	Pleurotaceae	Funcia di panicaudu, funcia di masticogna, funcia di spina (63)	It is edible fresh or dried, cooked in oven, or roasted it has delicious flavor and taste (63)
*Pleurotus eryngii *(DC.:Fr.) Quel. var. *ferulae *Lanzi	*Pleurotaceae *Fungo della ferula	Funcia di Levanzu, funcia di ferla, funci di ferra (63)	It is eaten fresh or dried, cooked in oven or roasted; it has delicious flavor and taste it is collected from the spring to last part of autumn (63)
*Pleurotus eryngii *(DC.:Fr.) Quel. var.*elaeoselini *Venturella, Zervakis & La Rocca	Pleurotaceae	Funcia di Dabs (63)	It is eaten fresh or dried, cooked in oven or roasted. It has delicious flavour and taste (63)
*Pleurotus eryngii *(DC.:Fr.) Quel. var.*thapsiae *Venturella, Zervakis & Saitta	Pleurotaceae	Funcia di firrazzolo (63)	It is eaten fresh or dried, cooked in oven or roasted it has delicious flavour and taste (63)
(Inz.) Quèl..	Pleurotaceae	Funcia di basiliscu, funcia di carmu, funcia dii li Madunni (63)	It is eaten fresh or dried, cooked in oven or roasted. It has delicious flavour and taste (63)
*Pleurotus opuntiae *(Durieu & Lévillé) Sacc.	*Pleurotaceae *Orecchione	Funcia di la ficurinia (63)	It is eaten fresh or dried, cooked in oven or roasted. It has delicious flavor and taste (63)
*Pleurotus ostreatus *(Jacq.:Fr.) Kummer	Pleurotaceae	Funcia di nipitedda, funcia di olivella(63), funci di olivella (64)	It is eaten (63) and consumed during summer and winter (64)
*Polyporus arcularius *(Batsch) Fries	Polyporaceae	Funcia di suvaru (63).	It is edible (63)
*Russula delica *Fries.	Russulaceae	Funcia di ilici, funcia piperita (63)	It is cooked in the oven with garlic, parsley, oil, salt and pepper (63)
*Russula Heterophylla *Fries	Russulaceae	Funcia di ferra (63)	It is edible (63)
*Russula virescens *(Schaeff.)Fr.	*Russulaceae *Colombina verdognola	Palummedda virdi (63)	It is edible (63)
*Sarcodon imbricatus *(L.:Fr) Karsten	*Bankeraceae *Steccherino bruno	Ventri di crapa (64)	It is edibleand consumed during summer and autumn (64)
*Suillus bellini *(Inzenga) Kuntze	Boletaceae		It is edible(63)
*Suillus collinitus *(Fries) O.Kuntze	*Boletaceae *Pinarello	Vavusi, funci i pigna (63)	It is edible(63)
*Suillus granulatus *(L.:Fr.) Roussel	*Boletaceae *Boleto granuloso, pinarolo	Vavusi, funci i pigna, funciu di zappinu, funciu di pignu (63)	It is ediblebut it has a strong laxative effect which can be reduced by removing the cuticle and most of the hymenium (63,64)
*Suillus luteus *(L.:Fr.) Roussel	*Boletaceae *Boleto giallo bruno	Vavusi, funci i pigna (63) Pinarolu, funciu di zappinu (64)	It is edible but has a strong laxative effect which can be reduced by removing the cuticle and most of the hymenium (63), from the last part of summer to the autumn (64)
*Terfezia arenaria *(Morris) Trappe	*Terzeziaceae *Tartufo delle sabbie	Traffulu, catatunfuli janchi (63)	It is eaten roasted or cooked (63)
*Tricholoma populinum *Lange	*Tricholomataceae *tricoloma del pioppo	Funci di chiuppu (64)	It is eaten and consumed during the autumn (64)
*Tricholoma terreum *(Schum.:Fr.) Kummer	*Tricholomataceae *Tricoloma terreo, agarico color crema, moretta	Funci i cannittu (2)	It is eaten and consumed from the summer to the autumn (64)
*Tricholoma tridentinum *Singer var.*cedretorum *M.Bon	Tricholomataceae	Funcia di cedru (63)	It is edible (63)
*Tuber aestivum *Vittad.	*Tuberaceae *Tartufo scorzone		It is edible (63)
*Volvariella gloiocephala *(D.C.:Fr.) Boekhout-Enderle	Pluteaceae	Funcia di la pagghia (63)	It is edible (63)
*Volvariella volvacea *(Bull.Fr.) Sing.	Pluteaceae		It is eaten well cooked (63)
*Xerocomus chrysenterion *(Bull.) Quèl.	*Boletaceae *Boleto dorato	Funciu di inestra, funciu di filici (64)	It is edible and consumed from the summer to the autumn (64)

## Discussion and results

The study conducted has consented the gathering of information on traditional uses linked with collective past memories and to nutritional habits of the Sicilian past conducted always in such a way so that they could take advantage of the territory resources. Furthermore, this has proven that even nowadays, in the island, there are ingredients and preparation methods used that remind us of ancient civilizations which have taken turns in dominating the island in the past centuries imposing their own customs. Greeks, Romans, Arabs, Normans, Svevian, and Spanish left a deep trace in the island not only concerning monuments and cultural traditions but also concerning the use of plants. *Centaurea calcitrapa *L., for instance, has been appreciated since the time of the Greeks and *Ceratonia siliqua *L., already known by the Romans, as showed by traces of its fruits in the pantries of the houses in Ercolano and Pompei after the eruption of the Vesuvio, are still as much used today as in the past. The researches conducted both in the internal part of the rural communities in Sicily and the coastal ones, have permitted to show that it concerns plants that are seasonally consumed, simply boiled or cooked in a pan with oil, garlic and pepper, often by also adding other vegetables; in fact, the expressions "ervi maritate" and "misticanza" which are recurrent, indicate the mixture of more vegetables in the preparation of soups and of other traditional dishes.

The study shows that 74 species are eaten boiled which are namely: 26 *Asteraceae*, 12 *Brassicaceae*, 9 *Liliaceae*, 6 *Fabaceae*, 3 *Chenopodiaceae*, 2 *Apiaceae*, 2 *Malvaceae*, 2 *Polygonaceae*, 2 *Urticaceae *and only 1 species of *Amaryllidaceae*, *Boraginaceae, Capparidaceae*, *Iridaceae, Juncaceae*, *Lamiaceae, Orchidaceae*, *Papaveraceae, Plantaginaceae *and *Solanaceae*.

62 are eaten raw or in salads and seasoned with oil, lemon or vinegar. Among them the *Asteraceae *are once more the most numerous with 23 species, following the *Rosaceae *with 8 species and the *Brassicaceae *and the *Apiaceae *with 6 species each, the *Moraceae *and the *Valerianaceae *with 3, the *Fabaceae *with 2 and the *Ericaceae*, the *Liliaceae*, the *Malvaceae*, the *Myrtaceae*, the *Oxalidaceae*, the *Palmae*, the *Portulacaceae*, the *Punicaceae*, the *Rubiaceae*, the *Scrophulariaceae *and the *Ulmaceae *with only one species. 55 species are fried for preparing side-dishes, vegetarian stuffing balls and omelets among them 15 *Asteraceae*, 10 *Brassicaceae*, 8 *Liliaceae*, 5 *Fabaceae*, 3 *Chenopodiaceae*, e only 1 of *Agavaceae*, *Amaranthaceae*, *Apiaceae*, *Boraginacea*e, *Cactaceae*, *Capparidaceae*, *Caprifoliacea*e, *Caryohyllaceae*, *Dioscoreaceae, Plantaginaceae, Ranunculaceae*, *Smilacaceae, Solanaceae *and *Valerianaceae*.

2 *Asteraceae *and 2 *Liliaceae *and only 1 species of *Orchidaceae *and *Oxalidaceae *are eaten roasted and seasoned with oil and salt. They are altogether 6 species, while 13 are used to prepare stews and namely 4 *Asteaceae *and 4 *Fabaceae*, 3 *Brassicaceae *and only 1 of *Lamiaceae *and *Liliaceae*.

Among the "*erbe maritate*" and "*misticanze *": 12 *Asteraceae*, 4 *Brassicaceae*, 2 *Caryophyllaceae*, 2 *Chenopodiaceae*, 2 *Fabaceae*, 2 *Urticaceae *and only 1 species of *Apiaceae, Boraginaceae *and *Portulacaceae*.

2 *Apiaceae *and 2 *Capparidaceae *and only 1 species of *Brassicaceae*, *Liliaceae, Myrtaceae *and *Oleaceae *are pickled or conserved in oil or vinegar. 4 *Rosaceae, 3 Moraceae *and 1 of *Cactaceae, Ericaceae*, *Myrtacea*e, and *Rubiaceae *are used to prepare jams, and preserves of fruits. 3 *Lamiaceae*, and only 1 species of *Lauraceae, Rosaceae *and *Rubiaceae *are used to prepare liquors.

The aromatic species are 37 and namely 13 *Lamiaceae*, 9 *Liliaceae*, 4 *Apiaceae, 4 Asteraceae *and only 1 species of *Brassicaceae*, *Fabaceae, Lauraceae*, *Papaveraceae, Pinaceae, Polygonaceae *e *Rosaceae*.

The fruits are eaten fresh, dried or in a jam. Among the wild species such as: *Crataegus azalorus *L., *Cydonia oblonga *L., *Pyrus amigdaliformis *Vill., *Rosa canina *L., *Rubus ulmifolius *Schott., *Sorbus domestica *L., *Celtis australis *L., *Myrtus communis *L., *Sambucus nigra *L. e *Arbutus unedo *L., it is preferable to eat only small quantities in order to prevent unpleasant health implications; the "pruna servaggi" (*Prunus spinosa *L.), were commonly eaten in the past.

The fruits of *Morus alba *L. and *M. nigra *L., *Pistacia vera *L., *Prunus dulcis *(Miller) D.A. Webb., *Castanea sativa *Miller, *Corylus avellana *L., *Punica granatum *L., and di *Opuntia ficus-indica *(L.) Miller, are used very often, as those are all plants which have been introduced to different cultures in antiquity.

This last species have adapted so well along the coasts of the island so as to become a typical specimen of the Sicilian flora and also one of the symbols of the island. Its fruits are often eaten; jams and typical sweets are prepared such as: mustard which in Castelbuono, a village in the Park of Madonie, is called "pizichintì". With the skins of the fruit and the cladodes "pale", fried with oil, side dishes or sweet omelets are prepared if sugar is added. Other fruits which are considered a specimen of the Sicilian flora, because they have also acclimatised very well in the island, are the citrus fruits, rutacee which have origins in eastern and central Asia. Those species have been introduced in Europe and widely cultivated, spreading in the Mediterrenean area, particularly in Sicily where they have nevertheless never shown the tendency to a spontaneous growth.

*Citrus sinensis *(L.) Osbek, (sweet orange),*Citrus Limon *(L.) Burm. f. (lemon) and *Citrus deliciosa *Ten. (Mandarin orange), which is not reported in the summarizing table, because only the species with a big variety of cultivars in the island are cultivated.

Concerning the mandarin orange, big merit goes to the Botanical Gardens of Palermo, where between 1810 and 1820 the first specimen of *Citrus deliciosa *Ten. was introduced and in less than 30 years time from its introduction in Sicilian cultivation, it became one of the species which most changed the agricultural landscape of the island, contributing in generating the famous "Conca d'oro" (big extension of citrus with golden-like coloured fruits). Among the plants of big interest in Sicily for the production of edul fruits, the *Vitis vinifera *L. occupies one of the first places. Cultivated since the antiquity, it spread sporadically in the island. The fruit is eaten both fresh (grapes) and dried (raisin). The lattest is used to prepare traditional sweets "buccellati", cookies, cakes or can be added to various condiments.

The varieties of cultivated vine are numerous and are generally placed into two categories: vine used for the production of wine or vine used as fruit. The ones used to produce wine are collected between September and October. Also the olive tree (*Olea europaea *L.) plays an important role in the local economy. From *Olea europaea *L. var. *sylvestris *Hoffmgg et Link (wild olive) which grows spontaneously in Sicily and along the coasts of the Mediterranean Sea, derive the numerous cultural varieties of domestic olive oil, widely cultivated because of its edible fruits and its production of oil.

The olives, conserved or eaten with bread have been a characteristic food for Sicilian farmers for many centuries as is also the oil produced from them. Both green and black olives are eaten; they are first marinated in water, which must be changed every day, until they lose their bitter taste. They are eaten seasoned with bay, oregano, rosemary, thyme; in "brine" and also mashed.

### Food plants used for different culinary purposes

#### Boiled

*Ammi majus *L., *Anthemis precox *L., *Apium nodiflorum *L., *Asparagus albus *L., *Asparagus officinalis *L., *Asphodeline lutea *L., *Asphodelus microcarpus *Salmz. et Viv., *Atractylis gummifera *L., *Barlia robertiana *Loisel, *Beta vulgaris *L. subsp. *vulgaris, Beta vulgaris *L. subsp. *maritima *(L.) Arcang., *Borago officinalis *L., *Brassica nigra *(L.) Koch, *Brassica rapa *L. subsp.*sylvestris *(L.) Janchen,*Brassica tournefortii *Gouan, *Bunias erucago *L., *Capparis spinosa *L., *Cardus argyroa *Biv., *Carlina sicula *Ten., *Centaurea calcitrapa *L., *Centaurea nicaeensis *All., *Centaurea solstitialis *L. subsp. *schouwii *(DC.)Dostal, *Chondrilla juncea *L., *Cichorium intybus *L., *Chenopodium album *L. subsp.*album, Chrysanthemum coronarium *L., *Cynara cardunculus *L.subsp. *cardunculus, Crepis bursifolia *L., *Crepis vesicaria *L. subsp. *vesicaria, Cydonia oblonga *L., *Cynara cardunculus *L. subsp. *cardunculus, Hedysarum coronarium *L., *Hermodactylis tuberosus *(L.) Salish., *Hirschfeldia incana *(L.) Lagr. F., *Hyoseris radiata *L., *Juncus acutus *L., *Lactuca viminea *(L.) Presl., *Lathyrus articulatus *L., *Lathyrus odoratus *L., *Lathyrus sylvestris *L., *Lavatera trimestris *L., *Leontodon tuberosus *L., *Leopoldia comosa *(L.) Parl., *Lupinus albus *L., *Lycium europaeum *L., *Malva sylvestris *L., *Moricandia arvensis *(L.) DC.,*Narcissus tazetta *L.subsp. *tazetta, Onopordum illyricum *L., *Papaver rhoeas *L. subsp. *rhoeas, Pisum sativum *L. subsp. *sativum, Plantago lagopus *L., *Raphanus raphanistrum *L. subsp. *raphanistrum, Raphanus raphanistrum *L. subsp. *landra *(Moretti) Bonnier, *Rapistrum rugosum *(L.) All., *Rumex crispus *L., *Rumex thyrsoides *Desf., *Ruscus aculeatus *L., *Ruscus hypoglossum *L., *Scolymus grandiflorus *Desf., *Scolymus hispanicus *L., *Sinapis alba *L., *Sisymbrium irio *L., *Sisymbrium officinale *(L.) Scop., *Smilax aspera *L., *Sonchus oleraceus *L., *Taraxacum officinale *Weber, *Teucrium fruticans *L., *Tolpis virgata *(Desf.) Bertol., *Tragopogon crocifolius *L., *Tragopogon porrifolius *L., subsp.*australis *(Jordan) Br.-Bl.*Urospermum picroides *(L.) Schmitd, *Urtica membranacea *Poiret, *Urtica urens *L..

#### Raw in salads

*Ammi majus *L., *Anthemis precox *L., *Apium graveolens *L., *Apium nodiflorum *L., *Arbutus unedo *L., *Carthamus lanatus *L., *Celtis australis *L., *Centranthus ruber *L., *Ceratonia siliqua *L., *Chamaerops humilis *L., *Chondrilla juncea *L., *Cichorium intybus *L., *Chrysanthemum coronarium *L., *Crataegus azarolus *L., *Crataegus monogyna Jacq*. subsp. *monogyna, Diplotaxis erucoides *(L.)DC., *Diplotaxis tenuifolia *(L.)DC., *Eryngium campestre *L., *Fedia cornucopiae *(L.) Gaertner, *Ficus carica *L., *Hyoseris radiata *L., *Hypochoeris cretensis *(L.) Chaub.et Bory, *Lactuca serriola *L., *Lactuca viminea *(L.)Presl., *Lathyrus articulatus *L., *Leopoldia comosa *(L.)Parl., *Malva nicaensis *All., *Mespilus germanica *L., *Morus alba *L., *Morus nigra *L., *Myrtus communis *L., *Nasturtium officinale *R. Br., *Notobasis syriaca *(L.) Cass., *Oxalis pes-caprae *L., *Picris echioides *L. Roth., *Portulaca oleracea *L., *Prunus spinosa *L., *Punica granatum *L., *Pyrus amygdaliformis *Vill., *Raphanus raphanistrum *L., *Raphanus raphanistrum *L. subsp. *raphanistrum, Reichardia picroides *(L.) Roth, *Ridolfia segetum *Moris, *Rosa canina *L., *Rubia peregrina *L., *Rubus ulmifolius *Schott., *Scolymus grandiflorus *Desf., *Scolymus hispanicus *L., *Scolymus maculates *L., *Silybum marianum *L., *Sisymbrium irio *L., *Smyrnium olusatrum *L., *Sonchus asper *L., *Sonchus asper *(L.) Hill subsp. *nymanii *(Tineo et Guss.) Hegi, *Sonchus oleraceus *L., *Sonchus tenerrimus *L., *Sorbus domestica *L., *Tragopogon crocifolius *L., *Tragopogon porrifolius *L. subsp. *australis *(Jordan) Br.-Bl., *Urospermum picroides *(L.) Schimtd, *Valerianella eriocarpa *Desf., *Veronica anagallis-aquatica *L..

#### Fried, browned in oil, in meat and vegetables balls, in omelettes

*Agave americana *L., *Allium ampeloprasum *L., *Amaranthuis retroflexus *L., *Asparagus acutifolius *L., *A.albus *L., *A. officinalis *L., *Asphodeline lutea *L., *Beta vulgaris *L. subsp. *maritima *(L.) Arcang., *Beta vulgaris *L. subsp. *vulgaris, Borago officinalis *L., *Brassica nigra *(L.) Koch, *Brassica rapa *L. subsp. *sylvestris *(L.) Janchen, (L.) Janchen *Capparis spinosa *L., *Carduncellus pinnatus *(Desf.) DC., *Cardus argyroa *Biv., *Cardus pycnocephalus *L., *Chenopodium album *L., *Chondrilla juncea *L., *Clematis vitalba *L., *Cynara cardunculus *L.*subsp. cardunculus, Diplotaxis erucoides *(L.) DC., *Erucastrum virgatum *(Presl) Presl, *Fedia cornucopieae *(L.) Gaertner, *Foeniculum vulgare Miller *subsp.*vulgare, Hedysarium coronarium *L., *Hirsfeldia incana Hyoserys radiate *L., *Hypochoeris laevigata *L., *H.radicata *L., *Lathyrus clymenum *L., *Lathyrus ochrus *(L.) DC., *Lathyrus odoratus *L., *Lathyrus sylvestris *L., *Leopoldia comosa *(L.) Parl., *Lycium europium *L., *Onopordum illyricum *L., *Opuntia ficus-indica *(L.) Miller, *Plantago serraria *L., *Raphanus raphanistrum *L., *R. raphanistrum *L. subps. *landra *(Moretti) Bonnier, *Rapistrum rugosusm *(L.) All., *Ruscus aculeatus *L., *Ruscus hypoglossum *L., *Sambucus nigra *L., *Scolymus grandiflorus *Desf., *Scolymus hyspanicus *L., *Silene vulgaris *(Moench.) Garcke *subsp. angustifolia *(Miller) Hayek, *Sylibum marianum *L., *Sinapis arvensis *L., *Smilax aspera *L., *Sisymbrium officinale *(L.) Scop., *Sonchus oleraceus *L., *Tamus communis *L., *Taraxacum offcinalis *Weber. *Urospermum dalechampii *(L.) Schmitd.

#### Roasted

*Allium ampeloprasum *L., *Barlia robertiana *Loisel, *Cynara cardunculus *L. subsp. *cardunculus, Hermodactylis tuberosus *(L.) Salsh., *Leopoldia comosa *(L.) Parl., *Oxalis pes-caprae *L.

#### Stewed

*Atractylis gummifera *L., *Calamintha nepeta *(L.) Savi, *Cynara cardunculus *L. subsp. *cardunculus, Hirschfeldia incana *(L.) Lagr.F., *Lathyrus clymenum *L., *Lathyrus ochrus *(L.) DC., *Lathyrus odoratus *L., *Pisum sativum *L. subsp. *sativum, Raphanus raphanistrum *L., *Raphanus raphanistrum *L. subsp. *landra *(Moretti) Bonnier, *Ruscus aculeatus *L., *Silybum marianum *L., *Tamus communis *L..

#### Mixed soups

*Bellis perennis *L., *Beta vulgaris *L. subsp. *maritima *(L.) Arcang., *Beta vulgaris *L. subsp. *vulgaris, Borago officinalis *L., *Brassica nigra *(L.) Koch, *Foeniculum vulgare *Miller subsp. *piperitum *(Ucria) Coutinho, *Hypochoeris levigata *L., *Hypochoeris radicata *L., *Hypochoeris radicata *L. subsp. *neapolitana *(DC.) Guadagno, *Lathyrus articulatus *L., *Lathyrus clymenum *L., *Portulaca oleracea *L., *Raphanus raphanistrum *L., *Reichardia picroides *(L.) Roth., *Silene vulgaris *(Moench.) Garcke, *Silene vulgaris *(Moench.) Garckesubsp. *angustifolia *(Miller) Hayek, *Sinapis arvensis *L., *Sisymbrium officinale *(L.) Scop., *Sonchus asper *L., *Sonchus asper *(L.) Hill subsp. *nymanii *(Tineo et Guss.) Hegi, *Sonchus oleraceus *L., *Sonchus tenerrimus *L., *Tragopogon crocifolius *L., *Urospermum dalechampii *(L.) Schmitd., *Urospermum picroides *(L.) Schmitd., *Urtica membranacea *Poiert, *Urtica urens *L..

#### Preserved in olive oil, brine or others

*Allium ampeloprasum *L., *Apium nodiflorum *L., *Arabis turrita *L., *Capparis ovata *Desf., *Capparis spinosa *L., *Myrtus communis *L., *Foeniculum vulgare *Millersubsp. *vulgare, Olea europea *L.

#### Jam

*Arbutus unedo *L., *Crataegus azalorus *L., *Crataegus monogyna *Jacq., *Cydonia oblonga *L., *Ficus carica *L., *Morus alba *L., *Morus nigra *L., *Myrtus communis *L., *Opuntia ficus-indica *(L.) Miller, *Rubia peregrina *L., *Rubus ulmifolius *Schott.

#### Liqueurs

*Laurus nobilis *L., *Menta spicata *L., *Menta suaveolens *Ehrh., *Rubia peregrina *L., *Rubus ulmifolius *Schott, *Teucrium scordium *L..

#### Spices

*Allium ampeloprasum *L., *Allium nigrum *L., *Allium roseum *L., *Allium sativum *L., *Allium triquetrum *L., *Allium schoenoprasum *L., *Apium graveolens *L., *Asparagus acutifolius *L., *Asphodeline lutea *L., *Brassica fruticolosa *Cyr., *Calamintha nepeta *(L.) Savi, *Ceratonia siliqua *L., *Crithmum maritimum *L., *Cydonia oblonga L., Foeniculum vulgare *Miller subsp. *piperitum *(Ucria) Coutinho, *Helicrysum italicum *(Roth) Don , *Laurus nobilis *L., *Leopoldia comosa *(L.) Parl., *Mentha acquatica *L., *Mentha pulegium *L., *Mentha spicata *L., *Mentha suaveolens *Ehrh., *Origanum heracleoticum *L., *Origanum majorana *L., *Papaver setigerum *DC., *Petroselinum sativum *Hoffm., *Phagnalon saxatile *(L.), Cass., *Phlomis fruticosa *L., *Pinus pinea *L., *Rosmarinus officinalis *L., *Rumex scutatus *L., *Salvia officinalis *L., *Salvia sclarea *L., *Tanacetum vulgare *L., *Taraxacum officinale *Weber, *Thymus capitatus *(L.) Hoffmanns et Link, *Thymus spinolosus *Ten.

### Analogies between food uses of plants in Sicily and in other regions

The study has allowed showing how many species of the Sicilian flora are eaten, not only in Italy but also in many countries of the Mediterranean and many analogies between the uses of those plants in our island and in other territories have been discovered. After a bibliographical comparison, it results that *Apium graveolens *L., for instance, is eaten in North Africa, Cyprus, Spain, Turkey and many Italian regions as a seasoning or as a freshly boiled, green vegetable. A similar use has been recorded for *Apium nodiflorum *L. used in Sicily, Tuscany, Basilicata, Latium, Spain, Crete and Tunisia.

Common and similar uses are those of *Capparis spinosa *L., some species of *Asparagus *L., *Beta *L., *Brassica *L., *Sonchus *L., *Borago officinalis *L., *Olea europea *L., *Portulaca oleracea *L., *Punica granatum *L. and *Rosmarinus officinalis *L..

An analogy has also been shown for some species whose use is exclusive in the rural communities of the island but which are also similarly used in other Mediterranean countries. For istance *Barlia robertiana *Loisel., is eaten in San Pietro di Caltagirone, in the Catania province and also in Bodrum, in Turkey; *Cardus pycnocephalus *L., in the Petralie, mountain communities, in the Madonie which is a province of Palermo and in Cyprus; the seeds of *Lathyrus clymenum *L., in Sambuca di Sicilia, in the Agrigent province and in Tunisia; the shoots of *Narcissus tazetta *L subsp.*tazetta*. in Calatafimi in the Trapani province and in Egypt; *Plantago lagopus *L. in the Messina province and also in Tunisia and Crete;*Prasium majus *L. in the Palermo and Trapani provinces as also in Tunisia. A certain analogy has been discovered also among the customs of some Albanian etnical groups who settled in the past in Italy and namely the Arbëreshë Albanian etnical group in Lucania or that of Piana degli Albanesi in Siciliy.*Origanum heracleoticum *L., *Ruscus aculeatus *L., *Salvia officinalis *L., *Urospermum dalechampii *(L.) Schmidt-B. are used in the same way by both gropus. *Oxalis pes-caprae *L., *Punica granatum *L., *Allium triquetrum *L., *Ceratonia siliqua *L., show similar uses in Contessa Entellina (Siciliy), where lives a Greek-Albanian community and in Cyprus.

Noticeable similarities in the vernacular names have also been discovered. For example the name of "sparaci" and "sparangh'i" are respectively used in both Sicily and Cyprus to indicate some species of *Asparagus L*.; also "vurrania" and "voragho" for *Borago officinalis *L.; "finocchiu" and "finokyo" for *Foeniculum vulgare *Miller subsp.*vulgare*; "lipini" and "loupinarya" for *Lupinus albus *L. [42]. In Tunisia [31,40]*Capparis spinosa *Desf. is known like "kabbar" or "quibbar" and in Siciliy like "chiappara"; *Cichorium inthybus *L. like "chkonia" and "cicoria" in our island, *Cynara cardunculus *L. subsp *cardunculus *like "kardouni" and "carduni" in Sicily.

### Medicinal-food plants

The recipe used has also facilitated the gathering relevant information to the medicinal properties of many edible plants. In popular tradition, food is often indicated as medicine and the use of one herb or another is suggested, according to the cases. In order to avoid excessive constipation, it is recommended to consume the leaves of the *Beta vulgaris *L. and *Beta vulgaris *L. subsp. *maritima *(L.) Arcang. "gira", of *Borago officinalis *L. "vurrania", the tender parts of *Cichorium intybus *L. "cicoria", which are also considered refreshing; in order to encourage diuresis and to purify the organism, *Foeniculum vulgare *Miller subsp. *vulgare *"finocchiu" leaves should be used.

Sometimes the same plant can have different effects. For example, the fruit of *Opuntia ficus-indica *(L.) Miller "ficurinnia" can cause constipation if consumed with the seeds, without the seeds it becomes a laxative instead.

They are 37 plants among which *12 Asteracae*, 4 *Lamiaceae*, 3 *Brassicaceae*, 3 *Apiaceae*, 2 *Liliaceae*, 2 *Chenopodiaceae*, 2 *Malvaceae*, 2 *Rosaceae *and only 1 species of *Boraginaceae*, *Cactaceae, Fabaceae*, *Lauraceae*, *Moraceae*, *Polygonaceae *e *Ulmaceae*. They are: *Asparagus acutifolius *L., *Asparagus albus *L., *Beta vulgaris *L. subsp. *vulgaris, Beta vulgaris *L. subsp. *maritime *(L.) Arcang., *Borago officinalis *L., *Brassica nigra *(L.) Koch, *Celtis australis *L., *Centaurea calcitrapa *L., *Centaurea nicaeensis *All., *Chondrilla juncea *L., *Cichorium intybus *L., *Crataegus monogyna *Jacq., *Crepis vesicaria *L. subsp. *vesicaria, Cynara cardunculus *L. subsp. *cardunculus, Diplotaxis erucoides *(L.)DC., *Eryngium campestre *L., *Ficus carica *L., *Foeniculum vulgare *Millersubsp. vulgare,, *Hedysarum coronarium *L., *Hyoseris radiata *L., *Laurus nobilis *L., *Lavatera trimestris *L., *Malva nicaensis *All., *Opuntia ficus indica *(L.) Miller,*Origanum heracleoticum *L., *Origanum majorana *L., *Origanum vulgare *L., *Raphanus raphanistrum *L. subsp. *landra *(Moretti) Bonnier, *Reichardia picroides*(L.) Roth., *Ridolfia segetum *Moris, *Rosmarinus officinalis *L., *Rumex crispus *L., *Scolymus hispanicus *L., *Sonchus oleraceus *L.*,, Sonchus tenerrimus *L., *Sorbus domestica *L., *Taraxacum officinale *L..

### Potentially toxic plants

Furthermore, during the course of the research, some interesting data on the potential toxicity of some plants has emerged. In fact, in the Sicilian popular tradition, as already mentioned in a previous contribution [1], the food plants are separated into two categories: refreshing and purifying "erve frische" or are endowed with stimulating and disturbing effects "erve caure". The immoderate use of the last category may have toxic effects; that is why *Sisymbrium officinale *and some species of *Sinapis *should be consumed moderately; others like *Capparis ovata *Desf. and *C. spinosa *L. must be "cured" ("curate") namely kept in water or boiled like *Lupinus albus *L. e *Smilax aspera *L.. Very dangerous are: *Clematis vitalba *L. and *Tamus communis *L., which can be only eaten after having been cooked, and *Atractylis gummifera *L., of which only the aerial part can be used.

### Edible seaweeds of traditional use in Sicily

It is important to underline that in Sicily the seaweeds are rarely used as food; their use is not well known and often limited only to some parts of eastern Sicily. According to Battiato's contribution [62] some *Rodophyceae *are considered to be edible in the island, for instance *Nemalion helminthioides *(Velley) Batters, (*Nemalionales*) a weed known in Palermo with the name of "Turkish spaghetti" ("spaghetti turchi"); *Hypnea musciformis *(Wulfen) Lamouroux (*Hypneaceae*) eaten in south-east Sicily; *Gigartina acicularis *(Wulfen) Lamouroux (*Gigartinaceae*), used to prepare omelettes in the Catania province where it is called "mauru rizzu", "capidduzzu" ; *Gigartina teedii *(Roth) Lamouroux (*Gigartinaceae*), known as "mauru, curaddina"; *Grateloupia proteus *Kuetzing (*Cryptonemalies*), called "mauru 'mpiriali", mauru ciuffu longu", mauru ciuffu curtu" and also used to prepare omelettes in the Catania province.

### Edible mushrooms of traditional use in Sicily

The results of the contributes [63-65] show that 78 mushrooms are eaten in different rural communities of Sicily. Quite commonly used are the *Pleurotus eryngii *(DC.: Fr.) Quélet var. *ferulae *Lanzi known like "funci di ferra" and *Pleurotus eryngii *(DC.: Fr.) Quélet var. *eryngii*, "funci di panicauru", called as such because it is referring to the Sicilian vernacular names of the two apiacee under which they grow. As Table 2 shows many other mushrooms are consumed raw or stewed in order to prepare delicious traditional dishes.

## Conclusion

Thanks to this study, and as also shown by the sinoptyc scheme (Additional file 1), the edible plants of traditional use in Sicily are 188; to those we must add *Olea europaea *L. and some species of *Citrus *L. commonly cultivated.

Some species are often used in the whole of the Sicilian territory: *Borago officinalis *for instance is used everywhere in Sicily while some others have a limited use in some areas of the island. This is the case of *Agave americana*, used only in Piazza Armerina; *Allium nigrum *in the Agrigent province;*Amaranthus retroflexus *in the Ragusa province; *Ammi majus *and *Anthemis praecox *respectively in Butera (CL) and Licata (AG); *Crithmum maritimum *in the Egadi Islands (TP); *Mentha pulegium *in the Aeolic Islands (ME); *Lathyrs sylvestris *in Messina, *Cynara cardunculus *in Agrigento and, sporadically, in the provinces of Palermo, Catania and Messina.

Exclusively in Sicily, like food plants are used 30 taxa. Among them, as shown by Pignatti in Flora d'Italia [60]*Allium nigrum *L., *Anthemis praecox *L.,*Arabis turrita *L., *Hedysarum coronarium *L.,*Lathyrus sylvestris *L,*Oryzopsis miliacea *(L.) Ash. et Schweinf,*Raphanus raphanistrum *L. subsp. *raphanistrum *and *Tolpis virgata *(Desf.) Bertol. are diffused in the whole of the Sicilian and Italian territory;*Hypochoeris levigata *L. is common only in Sicily and in the Island of Marittimo (TP), quite rare indeed in South Italy where, apart from Sicily, *Cardus agyroa *Biv. are common,*Centaurea nicaensis *All., *Diplotaxis crassifolia *DC. *Carlina sicula *Ten. and *Erucastrum virgatum *(Presl) Presl are endemic, while *Carduncellus pinnatus *(Desf.) DC., *Hermodactylis tuberosus *(L.) Salish., *Hypochoeris cretensis *(L.) Chaub. et Bory, *Hypochoeris radicata *L. subsp. *neapolitana *(DC.)Guadagno, *Lathyrus articulatus *L., *Notobasis syriaca *(L.) Cass., *Papaver setigerum *DC., *Phagnalon saxatile *(L.) Cass., *Plantago serraria *L., *Ridolfia segetum *Moris, *Ruscus hypoglossum *L.,*Ruscus hypophyllum *L.,*Teucrium fruticans L*. and *Teucrium scordium *L. are rather rare.

The discovery of their uses up to now not well known, is a stimulation to deepen the study on the plants of the local flora. Some wild species which are part of the daily life of the island, whome use seems to be new in the literature, could became object of bromatological and fitochimical studies. Those studies could possibly put on evidence the presence of substances important under the nutritional aspect that would allow emphasising species still unknown under this aspect and revaluing the natural and the cultural legacy of the island. With this work the authors, not only wish to give some information to attempt interdisciplinary studies but also hope to contribute in recovering the ancient culinary traditions and saving a legacy rich in information and intended to be lost if not written down. Since nowadays is growing the interest towards everything wihich is "natural" and it counts also for the food, the dishes which in the past connoted poor people could became today very "fancy". The continous flourishing of agritourism which offers traditional dishes to the visitors mostly prepared with the so-called "spontaneous vegetables", helps in spreading the knowledge and allows to appreciate their wholesomeness.

Reassesing the plants of the spontaneous flora, thus retrieving old traditional receipts, can help in introducing cultivations which could benefit the local economy.

## Supplementary Material

Additional file 1 Wild food plants used in Sicily Click here for file

Additional file 2 Sicilian vernacular names of food plants Click here for file
