# Supplementary material for: Wild food plants of popular use in Sicily
Source: J Ethnobiol Ethnomed. 2007 Mar 30;3:15. doi: 10.1186/1746-4269-3-15 (PMC1858679; doi:10.1186/1746-4269-3-15)
Supplement: Additional file 1 — Wild food plants used in Sicily [file 1746-4269-3-15-S1.pdf]

Table 1. Wild food plants used in Sicily

Scientific name<br>Family<br>Italian vernacular name	Parts used	Traditional food use in Sicily	Worldwide food uses recorded in the literature
Agave americana L. @<br>(*Agavaceae*)<br>Agave, Zammara	t. st	Fried in boiling oil.	The flowers are used to prepare "*pulque*" fermented wines used by the Indians of Latin America. The rhizome, when roasted, is used as food by the Indians of Arizona and Latin America [25].
Allium ampeloprasum L.<br>(*Liliaceae*) @<br>Porraccio	bu<br>le<br>yb	The bulb should be consumed steamed and seasoned with oil, salt and vinegar or should be preserved in oil; grounded and mixed with flour, eggs, parsley and cheese, it is used to prepare meat balls ready to be fried. It is also consumed roasted on ashes and seasoned with oil and salt. The young shoot (*u' tennìru*) and the bulb (*a' testa*) are used to season soups, sauces, chicken broth and veal. The leaves are used to add aroma to omelettes and to the "*olive cunsate*".	It is consumed as condiment for salads and soups in Sardinia [26]. The Albanian community Arbëreshë that lives in Basilicata uses the bulb as an aromatic seasoning [27], like the inhabitants of the small rural village of Castelmezzano situated in the same region [28].<br>It is consumed in many parts of southern Europe as Turkey [29] and Spain [30].
Allium nigrum L.<br>(*Liliaceae*)<br>Aglio maggiore	bu	Fried in oil, it is added to the tomato sauce in order to make it more aromatic.	
Allium roseum L.<br>(*Liliaceae*) @<br>Aglio roseo	bu	As seasoning.	A very precious seasoning in Tunisia [31], Crete [32], Spain [30, 33] and in many parts of the Mediterranean countries [25].
Allium sativum L.<br>(*Liliaceae*) @<br>Aglio comune	bu	In order to add aroma to meat, fish, and tomato sauce and as seasoning for cous-cous mixed with oil minced almonds (*mennuli pistate*) and parsley "*piddrusino*".	Cultivated and used as seasoning in various parts of the world [34].
Allium schoenoprasum L. @<br>(*Liliaceae*)<br>Erba cipollina	bu	Seasoning for salads and soups.	Aromatic seasoning in Garfagnana [35, 36, 37], Spagna [30] and Scotland [25].
Allium triquetrum L. @<br>(*Liliaceae*)<br>Aglio triquetro	bu	Seasoning for salads and traditional dishes with a base of cheese and olives.	Seasoning in Sardinia [26] and Turkey [38].
Amaranthus retroflexus L. @<br>(*Amaranthaceae*)<br>Amaranto comune	t. p	Boiled and pan-fried then seasoned with garlic and oil.	Nutritional plant in Crete [32] and in Turkey where it is known by the name of "*pancar otu*" [29, 38].<br>The young shoots of the "*u pedē rossē*" are consumed boiled and fried in Castelmezzano (Basilicata) [28] and in the Arbëreshë Albanian community that lives in the same region and that called this plant "nen"

			[39].
Ammi majus L. @<br>(*Apiaceae*)<br>Visnaga maggiore	le	Raw or boiled and seasoned with oil and lemon.	According PFAF the seeds are used as condiment [24].
Anthemis precox L.<br>(*Asteraceae*)<br>Camomilla precoce	t. p	Raw or boiled and seasoned with oil and lemon.	
Apium graveolens L. @<br>(*Apiaceae*)<br>Sedano comune	t. st	In order to add flavour to salads, meat soups and vegetables; it is consumed cut in cubes and mixed with goat cheese.	The plant is used as food in North Africa but also as digestible and carminative spices [31, 40, 41].<br>In Cyprus the same is noted as "*apion*" and is part of a typical salad called "*paysanne*" [42].<br>Green leaves and stalks are used as an ingredient in the soups, in Italy, England, America [25], Turkey [29] and Spain where are also used for salads [30]. The Arbëreshë Albanian community that lives in Basilicata uses the aerial parts as aromatic seasoning [27].<br>In Sardinia it is eaten raw in salads with cheese or in soup [26]. In Latium the whole plant is eaten raw and it is considered an aphrodisiac and lowers blood pressure [43].
Apium nodiflorum L. @<br>(*Apiaceae*)<br>Sedano d'acqua	ap	It is eaten boiled raw in salads, seasoned with oil and salt.	In the upper province of Lucca the aerial part of "*crescione*" is used raw in salads and the fresh juice is used as a digestive [44].<br>In Garfagnana is eaten in salads and soups [35, 36, 37]. In Versilia leaves and roots are used to add aroma to soups as and as appetizers [45]; the same use is noticed in Latium [43] and in Sardinia [26]. The Albanian community Arbëreshë of Basilicata use the aerial parts as seasoning, raw in salads or boiled like a soup [27]. Tender stem and leaves of this plants are also eaten in Spain [30, 33].
Arabis turrita L.<br>(*Brassicaceae*)<br>Arabetta maggiore	ap	Seasoned with oil, lemon and salt.	
Arbutus unedo L. @<br>(*Ericaceae*)<br>Corbezzolo	fr	The raw fruits are consumed or in ready-made marmalades jams with 1 kg of mature fruits, 600 gr of sugar, ½ lemon, ½ glass of maraschino cherries and ½ glass of water.	Fresh fruits of "albatro" are eaten in Garfagnana [35, 36, 37] in Versilia [45], in Sardinia [26], in the island of Elba [46], in Salento [47], in Basilicata [28]. In Liguria, is prepared with the fruits an alcoholic drink called "*vinetta*" [48]; in Latium are prepared marmalades with the fruits and the "*mosto cotto*" a vital ingredient in some traditional sweets [43].<br>In Cyprus with the pulp of "*arvoutos*" is prepared a liqueur which has aphrodisiac, sedative and anti-diarrhoea effects [42].<br>The plant was utilised in beekeeping for the preparation of the honey "*koumaromelo*" [42]. Also in Turkey [29] and Spain [30] the fruits are eaten raw or are used sometimes for making jam and liqueur, while the branches with leaves are used as preservative for olives only in Spain [30].

Asparagus acutifolius L. @<br>(*Liliaceae*)<br><u>Asparago pungente</u><br>(*)	tu	Boiled like vegetables in an omelette. Pan-fried with onion and oil, in order to add aroma to the tomato sauce or as seasoning for risottos.<br>Considered a medicinal food which has diuretic and purifying effects.	The turions of the plant have probably been an important part of human nutrition even before the discovery of the fire element [49]. In Garfagnana [35, 36, 37] and in Sardinia [26] they are consumed boiled or in omelette and they are used as a diuretic food medicine [33], in Latium they are cooked with the soups, in boiled water or steamed [43]. The ethnic Albanians Arbëreshë that live in Basilicata use the young shoot boiled and then fried with eggs [27], as the inhabitants of Castelmezzano in the province of Potenza [28].<br>In Cyprus the "*sparanghi*" are eaten as vegetables, used especially in the cases of anaemia [42]. In Turkey [29] and Spain [30] the young shoots are eaten stewed, in omelettes, sometimes in scrambled eggs and rarely raw as a snack. In Spain the shoots are used as medicinal food plant for theirs diuretic effects [33].
Asparagus albus L. @<br>(*Liliaceae*)<br><u>Asparago bianco</u><br>(*)	tu	Boiled and seasoned with oil and lemon, scrambled with eggs.<br>Considered medicinal food with a purifying and refreshing effect.	The leaves are used as food in Tunisia [31] and in many other countries of the Mediterranean region [25] as Sardinia [26] and Spain where young shoots are eaten stewed, in omelettes or with scrambled eggs [30].
Asparagus officinalis L. @<br>(*Liliaceae*)<br><u>Asparago comune</u>	tu	They are eaten boiled and seasoned with oil and lemon; pan-fried; covered in oil and with an aroma from bay leaves, pepper and carnation cloves.	It is a plant very much appreciated as nutritional since ancient times as proven through the writings of Plinius, Cato and others [25]. In Chieti the shoots are eaten raw or preserved in olive oil. The stiff stems are bound around salami to keep mice away [50].<br>In Latium [43]. and in Sardinia [26)] the young shoots are eaten boiled or in omelette and have a detoxifier effect. Same uses are recorded in Spain [30] Slovakia and in Croatia [51].
Asphodeline lutea L. @<br>(*Liliaceae*)<br><u>Asfodelo giallo</u>	fl<br>ap<br>ys	The flowers are eaten boiled and seasoned with oil, lemon, salt and pepper or covered with flour and fried; the tender stem is used for the preparation of omelettes together with asparagus; when fried it is added to the sauce for extra flavour.<br>The sprouts harvested before full bloom are taken out of their external skin, are cooked and fried with eggs.	Plyni says that the roots of this plant were generally roasted under embers and then eaten with salt and oil, mashed with figs to obtain an excellent dish [25].<br>In Palestine this plant is used as food which provides energy [52].
Asphodelus microcarpus Salzm. et Viv.<br>(*Liliaceae*)<br><u>Asfodelo mediterraneo</u>	ro	The underground parts are eaten boiled and with a bit of olive oil.	The underground parts are eaten in Sardinia and in Corsica [26].
Atractylis gummifera L. @<br>(*Asteraceae*)<br><u>Masticogna laticifera</u>	inf	The inflorescences "*caccocciulidda*" are consumed simply boiled or in a traditional stew seasoned with garlic, parsley and cheese.	The aerial parts are eaten raw or cooked in Sardinia [26], Spain [30] and in the the Arabian peninsula, the root is highly toxic [34]. However in north Africa root ashes are boiled in water then dried and mixed with semolina and butter. They are eaten in the morning as strengthening

			food [41].
Barlia robertiana Loisel.<br>(*Orchidaceae*)<br>Barlia	bu	Is eaten washed, cut in slices and roasted on the barbecue, seasoned with salt and oil, red chilli pepper, black pepper or rosemary, or just boiled.	The bulbs are eaten in Turkey in the Bodrum area [29].
Bellis perennis L. @<br>(*Asteraceae*)<br>Pratolina comune,<br>Margheritina	br	It is used to be added in vegetable soups in times of poverty or lack of other vegetables.	The leaves of "*margheritina*" are eaten in vegetables soups in the upper valley of the Serchio river (Tuscany) [37] and raw in salad in Sardinia [26]. In Britain and northern Europe the young flower buds and young basal leaves can be eaten on sandwiches, in soups and in salads. The buds, preserved in vinegar, can be used as a substitute for capers [53].
Beta vulgaris L. subsp. *maritima* (L.) Arcang. @<br>*(Chenopodiaceae)*<br>Bietola comune,<br>Barba; Barbabietola<br>(*)	le	They are eaten boiled and seasoned with olive oil and lemon; fried with garlic, bread crumbs, cheese and parsley; brown with tomatoes and garlic, chili pepper and small pieces of cheese; boiled and fried as filling for "*focacce scacciate*"; fried with potatoes for preparing the filling of the "*cuddiruni*" a typical kind of pizza. Also used as refreshing food and medical food in soups against constipation.	In (Tuscany) [35, 37, 44] and in Sardinia [26], the leaves are eaten boiled, stewed, in the soup, in omelettes or in stuffing for pies, the cooked leaves are used as "intestine cleansing"<br>In Latium, the leaves are boiled and eaten seasoned with olive oil and lemon as medicine food in case of constipation [43].<br>It is eaten along the Jonic coast [53].<br>In Turkey it is eaten raw [29]. In Cyprus it is used as purifying food [42] while in Spain as refreshing food useful against dyspepsia, constipation, stomach inflammation [33].
Beta vulgaris L. subsp. *vulgaris* @<br>(*Chenopodiaceae*)<br>Bietola comune, Barba; barbabietola<br>(*)	ap	It is eaten boiled and seasoned with olive oil, salt and black pepper; fried "*ngranciata*" (= browned) with garlic, olive oil and tomatoes; "*maritata*" (= married) with other wild vegetables or as ingredient for soups. It can also be boiled and fried with potatoes and onions and used for preparing the filling of the "*cuddiruni*" a typical kind of pizza of the province of Messina. Furthermore it is used as refreshing medicinal food or purifiers in soups against constipation.	In Tunisia the tender parts of the "*soukr lift ahmar*" are used alone or as an ingredient for preparing soups and ragout [31, 40]. The leaves, boiled, stewed with peas or put in omelettes are used as "intestine cleaning" in Tuscany [44]. On the Elba island it is considered as refreshing, laxative and cleansing, it is recommended for preparing soups [46], in Liguria the leaves of "bieda" are used to fill "*tortelli*" and "*torte di èrbi*" [48] as in Corsica [26].<br>Is called "*a jetē*" in Castelmezzano (Basilicata) and it is consumed boiled and fried in olive oil with chilli pepper; a typical dish of this village is "*u cauzonē pē le jetē*" a cake prepared with boiled leaves, cheese, flour and other ingredients [28].<br>In Turkey it is eaten raw, cooked or fried with eggs in oil [38].
Borago officinalis L. @<br>(*Boraginaceae*)<br>Borragine comune<br>(*)	le<br>fl<br>ap	The basal leaves are eaten boiled and seasoned with olive oil, lemon, and salt as vegetables; in soups or in omelettes; also boiled, minced and mixed with water and flour, are used to prepare the green *tagliatelle*. In the Etna region, during spring, people collect the upper parts (*i spicuni*) in order to prepare dishes of vegetables and soups such as the "*paparotta*".	This plant is used as human food since thousand years [49].<br>In Garfagnana (Tuscany) the leaves are eaten boiled, in soup, or as fillings for tortelli and for savour cakes. The aerial parts fried, boiled, stewed or used as stuffing for pies and tortelli [35, 36, 37].<br>This is a medicinal-food used for "intestinal cleansing" [44]. In Versilia (Tuscany) "*burana, borana, boraggine*" is one of the most used among the wild plants which find culinary application to prepare green pasta, ravioli and also a fennel-flavoured soup [45].<br>It is used in Tunisia for preparing soups with refreshing and soothing

		The leaves are seen as medicinal-food thanks to their refreshing, soothing, purifying and diuretic properties, they also help in the digestion and in curing constipation.	properties [31].<br>In Campania along the Amalfitan coast [55] and in the Elba island (Tuscany) is eaten as diuretic and purifying food, [46].<br>The leaves of "*boràsena*" are used, today like in the past in Liguria in the promontory of Caprione as filling of tortelli and "*torte di èrbi*" [48]. This wild species edible is also eaten in Salento [47] and in Sardinia [26]. The leaves of this plant, called *a* "*vurrascinē*" in Castelmezzano (Basilicata), are consumed in soup with other vegetables and generally served with cheese. The flowers called "a sucamelē" are sucked as snack [28]. The Albanian ethnic group Arbëreshë, which lives in Basilicata uses the leaves boiled and fried or in soup with beans called "*verajne e fazulje*" [28].<br>In Cyprus the "*voragho*" the leaves marinated with vinegar, are a very valuable food. According to the popular tradition the leaves must be used as purifying medicinal food [42]. In Spain it is eaten fried in butter [30] and the flowers are useful as medicine food for their diuretic properties [33]. Refreshing and healthy food widely used also in the northern part of Africa [41].<br>In Turkey it is eaten raw, simply cooked or fried with eggs [38].
Brassica fruticulosa Cyr.<br>(Brassicaceae)<br><u>Cavolo rapiciolla</u>	br<br>yb<br>inf	Very valuable vegetable in the Etna region, eaten fried with garlic oil and chili pepper above all as side dish to pork sausages. Is also eaten in the Eolic islands.	The leaves and the flowers of this species have been eaten since ancient times both raw and cooked [25].
Brassica nigra (L.) Koch @<br>(Brassicaceae)<br><u>Senape nera</u><br>(*)	ap<br>br	The lower part, still tender, is eaten boiled and seasoned with olive oil and lemon or cooked and fried with oil, garlic and tomatoes like side dish of typical meat-based meals.<br>The tender parts, boiled and seasoned with olive oil or added to the soup, are used as a laxative.	In Cyprus it is used as seasoning, stomachic and laxative. The seeds are used as seasoning [42].<br>In northern Africa is used both as food and for its stomachic and laxative properties [41].<br>This is the mustard of the ancients and it is cultivated in Alsace, Bohemia, Italy, Holland and England. The ancient Egyptian and Greeks used to eat the young plants as an equivalent to spinach and used the seed for preparing the mustard [25].<br>Today is used as greens in Turkey [29] and in Sardinia [26].
Brassica rapa L. subsp. *sylvestris* (L.)Janchen<br>(*Brassicaceae*)<br><u>Cavolo rapa</u>	br;<br>inf	The lower part, still tender, is eaten boiled and seasoned with olive oil and lemon or cooked and fried with oil, garlic and tomatoes. The young flowers (*sciuriddi*) are eaten boiled with salty water and mixed with eggs or fried in the pan.	
Brassica tournefortii Gouan @<br>(*Brassicaceae*)<br><u>Cavolo di Tournefort</u>	le	They are eaten boiled and seasoned with olive oil and lemon.	In Tunisia it is used as an alimentary plant [31].
Bunias erucago L. @	le	They are eaten boiled and seasoned with	In upper Lucca province (Tuscany) young aerial parts are eaten boiled, or

(*Brassicaceae*)<br>Cascellore comune		olive oil and salt.	in soup with other greens [36]. The cooked plants is used as a depurative and mild laxative [44]. In Sardinia it is used as fodder plant [26].
Calamintha nepeta (L.) Savi @<br>(*Lamiaceae*)<br>Mentuccia comune<br>Nepetella<br>Poleggio selvatico	le<br>yb	In order to season omelettes or other meals and specially the boiled "*babbaluceddi*" bolliti (white escargots). A typical dish of the Eolian Islands the "*nieputiddata*" is prepared by mixing the plant with garlic, pestle and mortar and cooking it with oil, tomatoes, adding eggs, some other vegetables and stale bread.	The leaves are used in Tuscany as condiment for mushrooms, vegetables, and sauces and as a digestive [35, 36, 37, 44].<br>In Versilia the "*nepitella*, mentuccia, *nipitella*" is often used to season different meals [45]. In Latium it is also used like seasoning for meat and many other meals [43], in the promontory of Caprione [48] like seasoning side-dish together with escargots. In Spain different parts of "*hierba nieta*", are used as herbal tea, for making liqueur or as a condiment for snails, soups, meat stews or olives [30].
Capparis ovata Desf.<br>(*Capparidaceae*)<br>Cappero peloso	fl. b<br>le<br>yb	The flower buds are preserved in salt, vinegar, olive oil and added in order to season salads during the summer or also sauces and various meals. The leaves and the young parts are boiled in salty water and seasoned with salt, oil and vinegar and are added to tomato-salad.<br>With capers, olives and tuna fish are prepared pasta sauces.	In Turkey it is used as a condiment [38].
Capparis spinosa L. @<br>(*Capparidaceae*)<br>Cappero comune	fl. b<br>le<br>fr	Before consuming the parts of the plant you need to "cure" them boiling them and changing the water for at least 2-3 days in order to eliminate a bitter substance; and they are preserved or under salt or under vinegar.<br>The leaves, boiled or seasoned with oil, salt, oregano and vinegar are eaten as vegetables; the small fruits "*cucunci*", kept in salty water, are used to season many dishes; the shoots are eaten boiled with oil and lemon or in omelettes. With capers, olives, garlic, oil, broccoli, chilli peppers, carrots and celery is prepared a traditional meal, the "*stemperata*".	Along the Amalfitan coast [55]. and in Sardinia [26] it is used as a condiment and also as digestive or appetizer.<br>In Latium usually the buds are preserved in salt or vinegar and used as appetizer [43].<br>The "*kabar*" is spreadly used as nutrition and as medicament in Tunisia [31, 40].<br>In Palestine, like in many others Mid East countries, "*caper busch, qubbar*" it is used as a condiment [40]. In the northern part of Africa its tender fruits are used as carminative [41]. In Cyprus is used a condiment made with tasty capers, salt, thymus, sesam, cumin, majoram, olive oil and vinegar to prepare the dressing for the "*khoritaiki*" salad used to stimulate appetite [42]. This plant is also considered a good condiment in Spain [30], Crete [32] and in Turkey [38]. The Greeks of the Crimea eat the young part as a very tasty food [25].
Carduncellus pinnatus (Desf.) DC.<br>(*Asteraceae*)<br>Cardoncello siciliano	ap	Fried with the batter or with eggs.	
Cardus argyroa Biv.<br>(*Asteraceae*)<br>Cardo argiroa	t. st	Boiled in salty water fried in butter or with eggs.	
Cardus pycnocephalus L.	le	Fried with butter or with eggs.	In Sardinia the stem it is eaten raw, stewed, in soup, in omelettes or

(*Asteraceae*)<br>Cardo saettone			browned with oil, tomatoes and onions [26] and in Cyprus the young leaves of "*cardos*" are eaten as vegetable [42].
Carlina sicula Ten.<br>(*Asteraceae*)<br>Carlina siciliana	ap	It is eaten boiled and the trunks (*trunzi)* boiled and seasoned with oil and vinegar are well appreciated for their peculiar taste similar to artichocke and hazelnuts	
Carthamus lanatus L. @<br>(*Asteraceae*)<br>Zafferanone selvatico	t. st	It is eaten raw as vegetable.	It is present on PFAF database but only for the edible oil that is obtained from the seeds [24], it is used in Sardinia as in Sicily [26].
Castanea sativa Miller@<br>(Fagaceae)<br>Castagno comune	fr	They are eaten both like dried fruits or cooked, boiled and sugared or roasted adding salt.	The chestnuts begun to be used as food thousand years ago [49].<br>They are eaten boiled, roasted, dried in Sardinia [26] and in Garfagnana (Tuscany) where are used to make a "polenta" extremely nutritious and pancakes [35, 37, 44].<br>During the Christmas' eve in Latium are eaten the boiled "*marroni*" with salt and bay leaves. Furthermore in the Viterbo province is made a soup of "*mosciarelle*" seasoned with tomatoes, oil, salt and stale bread [43].<br>The Arbëreshë Albanian community that lives in Basilicata uses the fruits boiled alone or to make cakes [27]. The inhabitants of the little village Castelmezzano (Basilicata) eat the seeds of "*a castagnē*" boiled with bay leaves and wild fennel or simply roasted [28]. In Spain the fruits of "*castañero*" are eaten raw, dried. roasted or stewed [30].
Celtis australis L. @<br>(Ulmaceae)<br>Bagolaro comune<br>(*)	fr	They are eaten fresh or dried. In the Ragusa province are eaten as a medicinal-food which helps to improve sight.	The fruit of this plant is eaten in southern Europe, temperate Asia and east India. The Greeks are very fond of this fruits [25].<br>In Sardinia [26] as in Spain [30] the fruits are used for making a liqueur while only in Spain they are eaten raw as medicine food for theirs astringent and diuretic properties [33]. It is also eaten in Salento [47].
Centaurea calcitrapa L. @<br>(*Asteraceae*)<br>Fiordaliso stellato,<br>Calcatreppoli<br>Calcatreppole, Ippofesto<br>Cardo riccio<br>(*)	le	They are boiled and seasoned with olive oil and lemon, then eaten as refreshing food.	In Sardinia it is eaten as in Sicily [26]<br>In northern Africa is used as food and for its stomachic and appetizing properties [41], in Spain it is used as food and for its diuretic action [30, 33]. In Cyprus the leaves of "*kalanthi*" are used as salad [42]. Young stems and leaves are eaten raw in salad now as the ancient time by the Egyptians [25].
Centaurea nicaeensis All.<br>(*Asteraceae*)<br>Fiordaliso<br>(*)	wp	It is eaten boiled and seasoned with oil, lemon and salt. It is a refreshing medicine-food.	
Centaurea solstitialis L. subsp. *schouwii* (DC.) Dostal@<br>(*Asteraceae*)<br>Fiordaliso giallo	wp	It is eaten boiled and seasoned with oil, lemon and salt.	The rosette of the young plant cut and washed is used in Latium to season soups called "*acquacotta*" [43].

Centranthus ruber L. @ (*Valerianaceae*) Valeriana rossa, Savonina, Camarezza comune	le	Raw are used to make tasty salads.	The leaves of "*canonèl*" are used in Liguria in Caprione promontory as nutritional herb [48].<br>Red valerian is eaten in many region of the southern Italy [25].
Ceratonia siliqua L. @ (*Fabaceae*) Carrubo	fr	The flesh of the sweet fruit used to be eaten from poor people and today is still appreciated from the children living in the countryside.<br>The flour is used in order to season the wine.	It was one of the favourite foods of the ancient Italians; they were founded in many houses in Pompei. These fruits were eaten in many part of the Mediterranean region by ancient populations. The Egyptians extracted from the husk of the pod a sort of honey with which they preserved fruits. These pods were imported into the Punjab as food for horses, pigs, cattle and poor people [25].<br>In Cyprus with the "*kharoubya*" is produced a molasses with a very aromatic taste which is eaten together with the "*tapini*" (pasta made out of sesam seeds) food with a great nutritious power [42]. In Turkey the flesh of the fruit is greatly appreciated [29, 38]. In Spain the fruits are used for making a chocolate or coffee substitute or are eaten rawor ground into flour. The leaves are used as preservatives for olives [30].<br>In Latium, a decoction of "carrube" and dried figs in wine and honey were given to the children suffering for dysentery or bronchitis [43]. It is also eaten in Salento [47] and Sardinia [26].
Chamaerops humilis L. @ (*Palmae)* Palma nana, Cefaglione	yb<br>ys	In Mazzarino and in Riesi, in the Caltanissetta province the bud of "*giafagliuni*" was considered to be a delicious food which was eaten for Christmas. Nowadays it is used in the Ragusa province (in Vittoria) the buds and the lower tender parts are eaten raw in salads seasoned with oil, salt, lemon and vinegar.	In Tunisia the buds and the fried stems are used to garnish the couscous [41]. The young shoots or suckers from the bottom of the plants are eaten in Italy. In Barbary the lower part of the young stems and roots are eaten by the Moors [25]. In Sardinia [26] and in Spain are eaten the raw fruits while the young shoots are used to making salads [30].
Chenopodium album L. subsp. *album* @ (*Chenopodiaceae*) Farinello comune	wp	It is eaten boiled or fried in the pan with olive oil, garlic and pepper. It is also used in order to prepare "risotti" and several fillings.	In Garfagnana the aereal parts of the "*cucina*" herb are eaten fried [35]. The leaves are collected in the summer and used in Latium to be cooked and eaten for tonic and antianemic purpose in case of tiredness [43]. This plant is also eaten in Crete [32] and in Turkey where it is called "*yabani hindiba*" [38]; while in Tunisia it is considered to be toxic for its great content of oxalic acid [31]. This plant used to be cultivated in the prehistoric villages of Switzerland; in the Hebrides Islands the leaves are boiled and eaten [25], as also in Spain [30] Unites States and Mexico where the seeds, which ground into the flour, are made into a bread or mush [25].
Chondrilla juncea L. @ (*Asteraceae*) Lattugaccio comune (*)	ap	During the winter the basal part "*a zzotta*" is collected; during the spring the floral axis "*u giummu*", in the summer the buds of the branches "*i taddi*" which are eaten cooked,	Wild species eaten in Sardinia [26], Salento [47], Turkey [29] and in Spain [30].

		raw or like the asparagus for preparing tasty omelettes. In Cattolica Eraclea, in the Agrigento province is considered a medicine-food which helps in falling asleep.	
Chrysanthemum coronarium L. @<br>(*Asteraceae*)<br>Crisantemo giallo	t. st	After being skinned, it is eaten both raw and cooked seasoned with oil and salt.	In Sardinia [26], Spain [30] and Turkey (Bodrum area) [29] the leaves are eaten as greens.<br>In Cyprus the "*khryssanthemon*" besides its use as holy flower during the Christian Easter, it was also used in pagan rituals in order to adorn temples and statues [42].
Cichorium intybus L. @<br>(*Asteraceae*)<br>Cicoria comune<br>(*)	wp	It is eaten raw or cooked seasoned with oil, salt and lemon; in soup, in stew or fried with garlic and oil.<br>The water in which the vegetable is cooked is drunk because of its positive effects on kidneys and liver. Furthermore it "cleans" the stomach and refreshes the intestine.	In Latium it is eaten boiled, browned in the frying pan with oil, garlic, chili peppers and is considered to be a medicine-food, detoxifier, purifier, diuretic and laxative. The chicory represents the main ingredient of the "*aquacotta alla maremmana*" traditional meal eaten by peasants of the Maremma region in Latium and Tuscany [43]. In Garfagnana fresh leaves are eaten as salads, filling for cakes and tortelli, soups, boiled in "mesticanze" and used as medicine-food, as "blood detoxifier" [35, 36, 37].<br>The leaves of the "cicoria, radichella" are greatly appreciated in Versilia [45]. and in Sardinia [26] in order to prepare salads and soups. The Albanian ethnical group Arbëreshë, living in Basilicata uses the "*cikour ma bathe*" boiled and fried with fava beans [27], while the inhabitants of Castelmezzano, in the same region, eat "*a cicoirē*" only boiled and fried [28]. This plant it is eaten also in Salento [47]. In Chieti, the leaves of chicory are eaten both raw and cooked; the roots toasted are used as substitute for coffee [50]. The same thing has been done during the second world war in Liguria [48] and in Spain [30].<br>In Tunisia the "*chkouria*" is used to prepare soups and salads which have diuretic [40]refreshing and laxative [41] properties, same in Spain [33].<br>The Cypriots used the leaves of "*kikhorio*" as salads or vegetables with a purifying stomachic and eupeptic property [42]. This plant is also eaten in Crete [32] and in Turkey where is also called "*citlik*" [38].
Clematis vitalba L.<br>(*Ranunculaceae*) @<br>Clematide vitalba	yb	Boiled and dripped in order to eliminate a toxic and bitter substance they are used to prepare omelettes, fillings and salads.	The young buds of "*vezzadro*" used as "blood cleansing" in Garfagnana [44] well cooked , are eaten, in omelettes, soups or fried in the pan [35, 36, 37] as in Sardinia [26] and in Latium [43].<br>The ethnic Albanians Arbëreshë that live in Basilicata use the young shoot of this plant boiled and then fried with bacon [27]. while the inhabitants of the village of Castelmezzano (in the same region) use the young shoots of "*a vitacchiē*" boiled and with scrambled eggs and cheese [28]. Same way to prepared young shoots of "*virgazas buenas*" is recorded in Spain [30].
Corylus avellana L. @	fr	They are widely used in the confectionary	The seeds of "nocella" are eaten well dried in Tuscany [36, 37] while they

(*Corylaceae*)<br>Nocciolo comune		field in order to produce sweets. In Bisacquino, in the Palermo province, the 6th of January toasted and blessed nuts are distributed to all the families as sign of good auspice.	are used in Sardinia, during Christmas holidays, for making cakes, cookies, "torroni" with honey and others dried fruits [26]. The kernel of "*a nocceddē*"/*a veddamē*" is eaten dried or roasted in Castelmezzano (Basilicata) [28]. In Spain the fruits of "*avellano*" are eaten raw, dried, added to cakes or stewed as a condiment [30].
Crataegus azarolus L. @<br>(*Rosaceae*)<br>Biancospino lazzarolo, Azzaloro	fr	They are eaten as fruit and sweet jam.	In Tunisia the fruits are eaten raw [31], while in Cyprus is prepared a delicious jam called the "*ladhapi*" [42].<br>In Latium are eaten the leaves raw in salad and also the flowers and fruits but in very small quantity for their astringent action [43].
Crataegus monogyna Jacq. subsp. *monogyna* @<br>(*Rosaceae*)<br>Biancospino comune, Azaruolo selvatico<br>(*)	fr	They are eaten fresh or in a sweet jam. In the Messina province, they are considered to be a medicine-food with relaxing properties.	The raw fruits of this wild species are eaten or are used for making jam in Salento [47], Basilicata [28], Sardinia, Corsica [26], Turkey [29], Spain [30] and Britain [53].
Crepis bursifolia L. @<br>(*Asteraceae*)<br>Radicchiella tirrenica	le	They are eaten boiled and seasoned with oil and lemon.	
Crepis vesicaria L. subsp. *vesicaria* @<br>(*Asteraceae*)<br>Radicchiella vescicosa<br>(*)	ap	They are eaten boiled and seasoned with oil and lemon. Medicine-food with laxative and diuretic properties.	In Garfagnana [35, 36] and in Sardinia [26] the leaves are eaten boiled and fried in the pan, while the Albanian ethnical group Arbëreshë living in Basilicata eats the whole plant boiled and fried [27], like also the inhabitants of the village of Castelmezzano in the Potenza province, who eat the "*marogliē*" both raw mixed in salad or boiled and fried with olive oil, garlic, chilli pepper and, sometimes, tomato sauces [28]. Also in Crete [32] and in Spain basal leaves are eaten raw in salads or stewed [30].
Crithmum maritmum L. @<br>(*Apiaceae*)<br>Finocchio marino	le<br>se	They are used like seasoning.	The raw leaves with an aromatic flavour have been always appreciated both as spice and as vegetable in Sardinia [26], and in Puglia where are used to garnish the dishes of fish [54].<br>In France, England [25], Spain. [30] Crete [32] and in Turkey [29] the leaves are eaten as greens, while in Cyprus they are preserved with vinegar and salt and used like seasoning [42].
Cydonia oblonga L. @<br>(*Rosaceae*)<br>Cotogno, mela cotogna	fr	The fruits cooked and mashed are mixed with sugar, lemon peel and juice and dried in order to prepare the energetic and sweet "*cotognata*"<br>They are also eaten in order to aromatise the wine *brulèe* together with carob fruits, cloves, orange peel and cinnamon.	The fruits of "melo cotogno" are used in Tuscany as in Epirus [57] or in Turkey [29] to prepare jams used also in case of diarrhoea and dissentery [37, 44].<br>In Sardinia a fruit sirup is used against intestinal pains [26] as also in Cyprus where the "*kidhonya*" sirup is spreadly used against the diarrhoea [42]. Always in Cyprus the ripe fruits are used to prepare the "*kydhonomeli*" a delicious aromatic jelly which is also seasoned with "*tioulli*" leaves (*Geranium roseum)* [42]. Also in Garfagnana [35] and in Basilicata (by the Albanian ethnical group Arbëreshë) [27], these fruits

			are used in order to prepare jams, while the inhabitants of Castelmezzano in the same region use the fruits of "*cutugnē*" boiled or roasted [28].
Cynara cardunculus L. subsp. *cardunculus* (*Asteraceae*) <u>Carciofo selvatico</u> (*)	t. st fl. b	The tender shafts and the young parts are used cooked and seasoned with oil, lemon and vinegar but also fried with eggs and parsley, boiled, stewed with onions, tomatoes and cheese; fried. The buds instead are cut in the middle and roasted or are cut in small slices and seasoned with oil and vinegar. They are eaten boiled and seasoned with oil and lemon; mixed with flour and fried; in omelettes or stewed with potatoes. This medicinal-food is recommended in the Agrigento province, due to its hypoglycaemic effect, because it protects the liver, as a purifier and also refreshing.	The stems of "a scalirē" are eaten by the inhabitants of Castelmezzano in Basilicata boiled and/or fried [28] as in Sardinia where are eaten also stewed [26]. In Latium, the young flower heads, still not blossomed, are eaten both cooked and preserved with salt [43]. The young leaves, the flower heads and the long and pulpy petioles of "*kinara i cactoss*" are been used since the ancient times on Cyprus as food, full of vitamins and mineral salts with digestive and aphrodisiac properties [42]. In Tunisia the "*kardoun*" is considered to be an essential vegetable for preparing some traditional dishes like the vegetarian couscous or other soups [31]. In Turkey (Bodrum area) the aerial parts are eaten as greens [29] as well as in Crete [32].
Diplotaxis crassifolia DC. (*Brassicaceae)* <u>Ruchetta pendolina</u>	ap	They are used boiled and seasoned with oil, garlic and chilli peppers.	
Diplotaxis erucoides (L.)DC. @ (*Brassicaceae*) <u>Ruchetta violacea</u> (*)	ap	They are used raw in salad; boiled and seasoned with oil and lemon, salt and pepper; fried and scrambled with eggs; fried in "balls" with eggs and cheese, tomato sauce and chilli peppers. Medicine-food appreciated despite its bitter flavour. A Sicilian proverb says: "*i cosi amari teneli cari*" (the bitter food must to be always present in your diet)	In Latium [43] the lower leaves are eaten cooked alone or together with other vegetables in a soup called "*misticanza*". The leaves are used raw in salads also in Salento [47], Sardinia [26], in Spain [30] and Sardinia where are also used fried with oil, onions and tomato sauces as condiment for pasta [26].
Diplotaxis tenuifolia (L.) DC. @ (*Brassicaceae*) <u>Ruchetta selvatica</u>	le	They are eaten in salads with oil and lemon.	The young leaves are eaten raw as salad in Abruzzo [50], in Tuscany [35, 36, 37], in Sardinia [26], in Latium [43], in Salento [47] and in Basilicata by the Albanian ethnical group Arbëreshë [27].
Erucastrum virgatum (Presl) Presl *(Brassicaceae)* <u>Erucastro</u>	ap	It is eaten boiled and fried with oil eggs and also with tomatoes, garlic and small cheese pieces.	
Eryngium campestre L. @ *(Apiaceae*) <u>Calcatreppola campestre</u>)	le; yb	They are eaten raw in salads with salt, oil, lemon and vinegar. In the Agrigento province it is used like medicine-food for its appetizing and laxative properties.	In the cuisine of Sardinia [26] and Latium [43] the tender leaves are used together with other vegetables and the young buds are eaten both raw in salads and cooked. The aerial parts are used in Spain for liqueurs or seasoning; young shoots are eaten raw or stewed, the roots are consumed raw as a snack in Spain [30].

Fedia cornucopiae (L.) Gaertner @ (*Valerianaceae*) <u>Lattughella</u>	wp	It is eaten raw and seasoned with oil and lemon or fried in the pan with eggs.	This species is cultivated in France as a plant mainly used for salads [25] and in Sardinia where it is called "*erba moddi*" that means"very tender greens" [26].
Ferula communis L. @ (*Apiaceae*) <u>Ferula comune</u> <u>Finocchiaccio</u>	mu	In Sicily don't'is eaten but the tasty mushroom "*funciu di ferla*" (*Pleurotus eryngii* (DC.: Fr.)Quélet var. *ferula* Lanzi which grows under it is used as seasoning to ragout and other sauces.	In Tunisia [31] and in Cyprus [42] the tender buds collected (not during the blooming period) are eaten raw or preserved with salt<br>The Cypriots appreciate the "*boarouthkia tis anathrikes*", mushrooms which grow under the "*kinos*" [42].
Ficus carica L. @ (*Moraceae)* <u>Fico comune</u> (*)	fr	They are used fresh, dried or in order to prepare "*viscotta di ficu*" and jams.<br>During the Christmas period they are also used to prepare "*buccellati*", sweets filled with figs-jam or "*purciddrane*" made out of flour, dough and dried figs.<br>Medicine-food used against the weariness.	In Garfagnana [35, 37] and in Sardinia [26] the figs are eaten raw or in jam. In Latium during the famine people used to eat the "*cuzzumbrigli*", small unripe figs boiled and seasoned [43]. Also the Albanian ethnical group Arbëreshë and the inhabitants of Castelmezzano in Basilicata, use the fruits like food [27, 28]. In Chieti the fruits are eaten as mild laxative and used for preparing jams [50]. With the latex the shepherds of Latium used to curdle the milk in order to obtain the cheese [43].<br>The fruits calles "*sika*" in Cyprus are eaten both fresh and dried with almonds or nuts and they are used to prepare an anise-flavoured jam [42].<br>In Tunisia the fig, called "*kerma*" [29], is eaten days both like fruit and syrup [31].
Foeniculum vulgare Miller. subsp. *piperitum* @ (Ucria) Coutinho (*Apiaceae*) <u>Finocchio comune</u>	se	Aromatic seasoning used for many traditional dishes.	The young aerial parts of "u fenucchiē salvaccē" are consumed in Basilicata, boiled and then served with broad beans purée; boiled in mixture with other wild vegetables and then served in various dishes, while the fruits, called "u jurē fenucchiē" are used to season many dishes (home made sausages) and a typical bread called "frisellē" [28]. The seeds and tender parts are eaten also in Spain [33].
Foeniculum vulgare Miller subsp. *vulgare* (*Apiaceae*) <u>Finocchio comune</u> (*)	se ap	The tender parts of the plant are eaten boiled and seasoned with oil and lemon or mixed with vegetables and soups, omelettes, pasta, tomato sauces. They are also an ingredient of the "*pasta chi sardi*" or "*pasta n'casciata*" prepared with onions, pine nuts, raisins, sardines and saffron.<br>The tender parts are also used to season the "*maccu ri favi*" traditional Sicilian meal with broad beans, onion, tomato and olive oil. From that it derives the proverb "*essere favi e finocchi*" to indicate two things which get along very good together.<br>Very tasty are the fried "balls" of wild fennel	The fennel was cultivated by the Romans as a garden herb used in the kitchen to flavouring meat or sauces, the seeds are employed for flavouring liqueurs. The diffusion of this plants in central Europe was stimulated by Charlemagne [25].<br>The seeds are used in the upper Lucca province as condiment especially with fried pig's liver and boiled chestnuts [36, 37, 44]; in Abruzzo and Sardinia to aromatize sausages foods and salami [26, 50].<br>The fennel is eaten in Salento [47] and in Liguria where "*er fenoceto*" is used to prepare soups, biscuits, sweets [48]; in Tuscany it is traditionally used as filling of the cake of "*ervi amari*" or like seasoning and to prepare the "*ramerino*" a round bread cooked with flour, raisin and pine nuts [37]. In Latium the leaves and the tender shafts are eaten raw in salads and are the principal ingredient to prepare the "*acquacotta*", "*strigoli*" and a soup with potatoes, zucchini, beans and green beans. The seeds

		made of leaves mixed with eggs, cheese and pine nuts. The seeds are traditionally used to season sausages, pork meat, olives and biscuits.<br>Also a medicinal-food used as an appetizer, a diuretic, refreshing and can help to protect the liver.	were used to season broad beans, olives, sausages, roasted pork chops or sweets [43]. The Albanian ethnical group Arbëreshë of Basilicata uses the seeds to season home made sausage "sauxic" and biscuit "taralli" [27].<br>In Tunisia it is used to season aromatic bread [31].<br>In Palestine and Israel it is used thanks to its digestive properties [59] as well as in Greece [57].<br>In north Africa, the seeds "*besbes*" are used as stomachic, digestive and carminative [40], in Cyprus the "*finokyo*" is used to season meat dishes, vegetables, soups and green olives [42], similar uses are recorded in Spain where the seeds are used also as preservative for dry figs or for preparing herbal tea or liqueur. [30].
Fraxinus ornus L. @<br>(*Oleaceae*)<br>Frassino da manna	st. j	The manna obtained by scatching the bark of the ash tree is a thick, sweet liquid used in order to sweeten the "*mannetto*" a tasty panettone made in Castelbuono in Madonie. Mountains. The manna can be taken dissolved in the milk against constipation and it is therefore considered a medicine-food.	This tree is the *melia* of Dioscorides, the *melos* of the modern Greece; the seeds are imported into Egypt for alimentary purposes [25].
Glycyrrhyza glabra L. @<br>*(Fabaceae)*<br>Liquirizia comune	ro	The root is chewed and has a sweet taste. It is used to prepare delicious candied fruits.	It is used in north Africa as aromatic, sweetener and against abdominal pains [41]. The root of this plant in Basilicata and Sardinia it is consumed raw as snack [28, 26]. The leaves, called "*nakhalsa*" are employed by the Mongols as a substitute for tea [25]. The roots and rhizomes of this plant called in Spain "*regaliz, regalissia*" are used to make a liqueur (ratafia) or digestive tea [30] or a infusions for the treatment of cold [33].
Hedysarum coronarium L.<br>*(Fabaceae)*<br>Sulla comune<br>(*)	ap	They are eaten boiled, seasoned with oil and lemon or scrambled with eggs and onions.<br>It is a medicine-food with laxative, refreshing and soothing properties.	
Helicrysum italicum (Roth) Don@<br>(*Asteraceae*)<br>Perpetuini d'Italia	le	It is used to season meat, fish and potatoes.	In Spain [30] and in Sardinia the flowered aerial part are used as herbal tea or as seasoning roasted meat and fish [26].
Hermodactylis tuberosus (L.)Salish.<br>(*Iridaceae*)<br>Bocca di lupo, Bella vedova	ro	In the Catania province they are eaten roasted or boiled without the skin and seasoned with oil and lemon.	
Hirschfeldia incana (L.)Lagr. F. @<br>*(Brassicaceae)*	inf	The tender flowers called "*sciuritti*" are eaten boiled or scrambled with eggs, fried, stewed with onions, tomatoes and peas.	In Crete [32] and Spain [30] the tender stems are used as food.

Senape canuta			
Hyoseris radiata L.<br>(*Asteraceae*)<br>Radicchio selvatico<br>(*)	br;<br>yb	They are eaten boiled and seasoned with oil and lemon; in soups with other vegetables or fried in the pan. The tender buds were also eaten raw by shepherds and peasants. It is a medicine-food with soothing and purifying properties used in the Trapani province.	In Versilia the "wild radicchio" is occasionally eaten like herb during the spring [45] as well as in Sardinia where this little green it is eaten raw in salad or boiled in traditional soup with others 18 wild edible herbs , onions and olive oil [26].
Hypochoeris cretensis (L.)Chaub. et Bory<br>(*Asteraceae*)<br>Costolina cretese	ap	They are eaten raw and seasoned with oil and lemon.	
Hypochoeris laevigata L.<br>(*Asteraceae*)<br>Costolina levigata	ap	They are eaten fried in the pan or boiled with the "cicoina" (*Urospermum dalechampi)* and seasoned with salt and olive oil.	
Hypochoeris radicata L.<br>(*Asteraceae*) @<br>Costolina giuncolina	ap	They are eaten boiled together with the "*cicoina*" (*Urospermum dalechampii)* seasoned with oil or fried in the pan with oil, garlic and other ingredients. The "*costolina*" is widely used in the Etna region. The stems with inflorescence "*micc'i scalora*" are eaten like the asparagus.	In Tuscany [35, 36, 37, 44] and in Sardinia [26] the young aerial parts, used as depurative food, are eaten raw in salads, boiled, in soup or stewed.<br>In Versilia the herb called "*grassaporci, grasselli, grugnetti, ingrassaporci, lardelli*" is normally used in order to prepare salads and fried vegetables [45]. Also the Albanian ethnical group Arbëreshë in Basilicata eats the plant boiled or fried [27].<br>Similar uses are recorded in Spain [30].
Hypochoeris radicata L. subsp. *neapolitana* (DC.) Guad.<br>(*Asteraceae*)<br>Costolina giuncolina	wp	It is eaten together with the cicoina (*Urospermum dalechampii*) and seasoned with oil or fried in the pan.<br>The "*zotta*" (which is the basal part of the plant where the leaves grow) is appreciated in the countryside for its taste despite the bitterness. It is eaten boiled and seasoned with oil and lemon or "*maritata*" (= together) with other plants. The aromatic stems with inflorescence, "*micc'i scarola*", and "*scaranzizuli*" are eaten after being boiled like the asparagus, otherwise they are also used as ingredient for omelettes.	
Juncus acutus L.<br>(*Juncaceae*)<br>Giunco pungente	yb	It is eaten boiled.	In the island of Maddalena (Sardinia) the tender rhizome is eaten raw [26]
Lactuca serriola L. @<br>(*Asteraceae*)<br>Lattuga selvatica, scarola	le	They are eaten like salad.	The leaves are eaten in Tuscany as salads [36, 37] and used as food medicine, depurative and "intestine cleansing" [44]. The young aerial parts of "lactucastrē" are consumed in Castelmezzano (Basilicata) [28],

			as also in some villages of the Spain raw in salad, boiled and/or fried [30].
Lactuca viminea (L.) Presl.<br>(*Asteraceae*)<br>Lattuga alata	br	It is a very tasty vegetable which is normally collected before the plant blooms. It is eaten boiled or in salads also seasoned with oil. The tender middle stem called "*cimuzzu*" is cooked like the asparagus.	In Spain the young shoots are eaten raw in salads [30].
Lamium flexuosum Ten.<br>*(Lamiaceae)*<br>Falsa-ortica flessuosa	fl	The children use to suck the flowers called "*sucameli*" because of their sweet flavour.	In Tunisia with the juice of the plant and with the flowers a delicious jam is prepared [31].
Lathyrus articulatus L.<br>*(Fabaceae)*<br>Cicerchia articolata	se	The seeds are cooked like the peas when they are tender and they can be also eaten raw.	
Lathyrus clymenum L.<br>*(Fabaceae)*<br>Cicerchia porporina	se	They are eaten boiled and seasoned with tomato, oil, salt and pepper; in omelettes or stewed with onions, oil and parsley.	The seeds are eaten boiled in Tunisia [31] and raw in salad in Sardinia [26].
Lathyrus ochrus (L.)DC. @<br>*(Fabaceae)*<br>Cicerchia pisellina	se	They are eaten boiled and seasoned with tomato, oil, salt and pepper; in omelettes or stewed with onions.	It was used to be eaten by the ancient Greeks and considered a precious food by the Egyptian [25].<br>Today it is also eaten in the Bodrum area in Turkey [29].
Lathyrus odoratus L. @<br>*(Fabaceae)*<br>Cicerchia odorosa	se	They are eaten boiled and seasoned with tomato, oil, salt and pepper; in omelettes or stewed with onions.	
Lathyrus sylvestris L.<br>*(Fabaceae)*<br>Cicerchia silvestre	inf	They are eaten boiled and seasoned with oil and lemon; scrambled with eggs and cheese; fried in the pan with onions in order to prepare delicious side dishes.	
Laurus nobilis L. @<br>(*Lauraceae*)<br>Alloro, Lauro<br>(*)	le	The dried leaves are used to season ragout and other dishes especially meat and game dishes or figs and mustard.<br>In Bisacquino it is used to make a traditional "*rosolio*" produced with 9-10 green leaves left in alcool at 90°, sirup, 1 liter of water and 700 g of sugar. It is used as digestive after the meals.	In Tuscany [35, 36, 37, 44, 45] the leaves are used to season sauces, boiled chestnuts, meats and sausages like in Latium [43]. Sardinia [26] and in Liguria where in the butcher shops it is still usual to see branches of bay beside the meat [48].<br>The Albanian ethnical group Arbëreshë in Basilicata uses the leaves as condiment and as digestive infusion [27].<br>Also in Tunisia [31, 40] the leaves are used for prepare and season food as well as in Cyprus where the leaves are used like seasoning the "*kleftiko*" a lamb-based dish [42]. In Spain the leaves of "*laurel*" are used as a condiment for stews or olives and for making liqueur (ratafia) [30].
Lavatera trimestris L. @<br>(*Malvaceae*)<br>Malvone reale<br>(*)	le	It is eaten boiled and seasoned with oil and salt. In the Agrigento province it is considered a medicine-food helpful against gastritis and constipation.	
Leontodon tuberosus L.	le	It is eaten boiled and seasoned with olive oil.	The leaves are eaten boiled in Tuscany with other vegetables and they are

(*Asteraceae*)<br>Dente di leone tuberoso			used as medicinal-food for "cleaning blood" [36, 44].<br>The tubers are eaten raw as a snack while the basal leaves are consumed well stewed in Spain [30] and in Sardinia [26].
Leopoldia comosa (L.) Parl. @<br>(*Liliaceae*)<br>Cipollaccio	bu	The bulb must be collected before the bloom and it is eaten boiled or fried like seasoning in omelettes, soups or sauces; it can also be cooked in the oven, roasted or preserved in vinegar becoming a delicious appetizer.<br>According to an ancient recipe of Ragaina, in the Catania province, the bulbs boiled in salty water, mashed and mixed with minced meat, eggs and cheese are used to prepare meat balls.	The bulb is also used in Crete for an alimentary purpose [32]. It is also a custom of the ethnic Albanian group Arbëreshë that lives in Basilicata and uses the bulb cooked after cutting it and macerating it in cold water overnight [27]. The bulb called by the inhabitants of Castelmezzano (Basilicata) "*u cipuddēnē*" is cooked, after maceration in water, in different way: fried with eggs, boiled and served with "*vin cuttē*" (mosto cotto), boiled in water or pickled in olive oil and aromatized with chilli pepper, garlic and mint [28]. In Sardinia it is eaten raw or stewed [26].
Lupinus albus L. @<br>*(Fabaceae)*<br>Lupino bianco	se	They are eaten boiled and served with oil and salt after having been "*curati*" (cured) i. e. washed for a couple of with salty water.<br>The toasted seeds were used to make the coffee-surrogate.	This plant has been cultivated since the times of the ancient Egyptians, Romans and Greek [25].<br>The ethic Albanian group, Arbëreshë that lives in Basilicata eats the boiled seeds [27]. In Latium it was given particularly to diabetic patients [43]. Also in Sardinia, Corsica [26] and Tuscany the seeds well boiled in salty water are often eaten [56]. The Peloponnesian inhabitants were scornfully called "*loupinarya*" eaters due to their poverty [42]. Also in Sardinia the roasted seeds were used to make coffee surrogate [26].
Lycium europaeum L. @<br>(*Solanaceae)*<br>Spina santa comune,	wp	It is eaten boiled and seasoned with oil, lemon and pepper or scrambled with eggs, cheese and onions.	
Malva nicaensis All. @<br>(*Malvaceae*)<br>Malva scabra<br>(*)	le	They are eaten raw in salads seasoned with lemon and oil. In Lucca Sicula and Piazza Armerina it is also considered a good medicinal food with a refreshingly laxative effect.	In Spain the immature fruits are eaten raw as a snack [30].
Malva sylvestris L. @<br>(*Malvaceae*)<br>Malva selvatica	le	The leaves are eaten boiled and seasoned with oil as medicinal food with a laxative, anti-inflammatory and emollient effect.<br>The tender fruits called "*panuzzi du Signuri*" are eaten as a pastime during country walks.	In Sardinia [26] as well as in the upper Lucca province (Tuscany) [36, 37] the leaves are eaten in soup , used also in this region as mild laxative [44]. In Spain the flowers are used as herbal tea and for making liqueur (ratafia) [30]. The same way to eat the immature fruits and the tender leaves and stems, is recorded in Sardinia, Turkey and Spain [26, 29, 30].
Mentha aquatica L. @<br>(*Lamiaceae*)<br>Menta d'acqua	le	They are used both fresh and dry as. aromatising for typical dishes.	In Sardinia the leaves are used as aromatising of the sausage of pig's blood called "*sanguinaccio*" and as filling, mixed with cheese and potatoes. for "*ravioli*" [26]. In Tuscany leaves are used as aromatising for liqueurs and omelettes [44]. The aerial parts of "té del rio" are used in Spain as herbal tea or as condiment [30].
Mentha pulegium L. @<br>(*Lamiaceae*)	le	They are used both fresh and dry as aromatising for typical dishes.	The leaves are used as spices and herbal tea in Turkey [29] and Spain where the tender stems with leaves are also used as condiment for the

Menta poleggio			soup "*gazpacho de invierno*" or as seasoning for snails [30]. Similar uses are recorded in Sardinia [26].
Mentha spicata L. subsp. *glabrata* (Lej et Court.) Lebeau (*Lamiaceae*)<br>Menta romana	le	They are used both fresh and dry as aromatising for typical dishes.	In Tuscany leaves are used as aromatising liqueur and omelettes [37]. and theirs infusion it is used as digestive [44].
Mentha spicata L. subsp. *spicata* (*Lamiaceae*)<br>Menta romana	le	Fresh leaves are used for the preparation of refreshing drinks or even for "rosolio" mint (liqueur), for "salmoriglio" a sauce prepared with olive oil, garlic, lemon juice, mint, oregano, laurel to aromatise meat and fish, as seasoning for stewed broad beans grounded with garlic, bread crumbs, salt and vinegar.	The ethnic Albanian group Arbëreshë that lives in Basilicata uses raw leaves as a condiment [27]; in Tuscany leaves are used as aromatising liqueur and omelettes [36, 44].<br>ln Tunisia the leaves of "*naanaa*" are used to aromatise the tea [31], while in Cyprus the plant called "*dhiosmi*" is used to season salads and other traditional dishes [42].
Mentha suaveolens Ehrh. @ (*Lamiaceae*)<br>Menta a foglie rotonde	le	It is used to aromatise grilled food, sauces and salads. With 20-30 leaves left and soaked in alcohol for 8 days, filtered and with the adjunction of 500 gr. of sugar you can obtain an excellent liqueur.	In Sardinia and in the upper Lucca province (Tuscany) the leaves are used to aromatise liqueur and omelettes [26, 44], in Tunisia they are used to aromatise tea and bread [31].
Mespilus germanica L. @ (*Rosaceae*)<br>Nespola volgare	fr	It is eaten as a fruit.	The fruits are eaten in Sardinia raw or cooked [26] as well as in upper Lucca province (Tuscany) [36, 37] where they are used as medicinal-food which helps to combat diarrhoea [44].<br>In Spain the fruits of "*nispero, ciparu, mizpira*" are eaten raw after stored [30]
Morus alba L. @ (*Moraceae*)<br>Gelso comune	fr	They are eaten both fresh and as a jam.	They are called "*tout abiad*" and are a precious food in Tunisia [40]. In Sardinia, Basilicata and Tuscany the mulberries are eaten raw [26, 28, 35, 37].
Morus nigra L. @ (*Moraceae*)<br>Gelso nero	fr	They are eaten both fresh and as jam.	In Sardinia, Basilicata and Tuscany the fruits are eaten raw [26, 27, 28, 37]. Syrup of the fruits in cold water is used in Latium [43]. The fruits called "*tout akhel*" in Tunisia and "*morea i melena*" in Cyprus are not only used as food but also as a vermifuge [40, 42].
Moricandia arvensis (L.)DC. (*Brassicaceae*)<br>Moricandia comune	wp	It is eaten cooled down and seasoned with oil and salt.	In Tunisia it is called "*ham*" and it is consumed cooled down and fried in a pan with garlic and pepper [31]. It is also used in Spain [30] and in Sardinia as edible vegetable [26].
Myrtus communis L. @ (*Myrtaceae*)<br>Mirto	fr<br>le<br>fl	The fresh fruits are used to prepare jam, the flowers are eaten and with the leaves the olives in brine are aromatised.	The fresh fruit and the leaves are used in Cyprus [42.], as well as in Latium [43], in Sardinia [26], in Tuscany [35], in Liguria [48] in the promontory of Caprione as an aromatic seasoning for pork, poultry and game and for making very delicious liqueur [26].<br>In Greece the plant was sacred to Venus and its fruit was eaten by the modern and the ancient Athenians [25]. In Spain the fruits of "*arrijan, mirto, murteras*" are eaten raw while the branches with leaves are used as

			a condiment for olives [30].
Narcissus tazetta L. subsp. *tazetta* (*Amaryllidaceae*) Narciso nostrale	inf	It is eaten blanched and seasoned with oil, lemon and salt or cooked with dry broad beans.	In the ancient Egypt, the leaves, which had an onion flavour, were used cooked together with grounded hazel-nuts in order to prepare a highly appreciated dish [25].
Nasturtium officinale R. Br. @ (*Brassicaceae*) Crescione d'acqua	ap le	The aerial part of the "*crisciuna*" is eaten blanched or raw in salads and seasoned with oil and salt.<br>It is believed that the water in which the plant is cooked can stimulate milk production after having given birth.	The young shoots and leaves have been used as a salad from ancient time by the Greek, Romans and Persians. A Greek proverb says "*eat cress and learn more*" [25].<br>In Latium, the nasturtium is eaten raw in salads together with tarassaco and chicory in a "*misticanza*" of vegetables or boiled as an ingredient of "*acquacotta*". The poor citizens used to eat it, many years ago, with bread when working on the fields and sellers used to sell it in the streets of Rome [43].<br>The ethnic Albanian group Arbëreshë that lives in Basilicata uses the leaves raw in a salad or boiled and fried [27], as the inhabitans of Sardinia [26]. The leaves are today as sought after as in the past, in Liguria in the promontory of Caprione, for the preparation of herbs and soups [48]. The plant is also used in Salento [47]. In Cyprus the leaves of "*nasturzion*" are eaten as vegetables and have a purifying and remineralising effect [42].<br>It is the same in Tunisia [31] where the plant is called "*heberched*" [40].
Notobasis syriaca (L.) Cass. (*Asteraceae*) Cardo siriano	t. st	They are eaten raw with bread and goat cheese.	
Onopordum horridum Viv. (*Asteraceae*) Onopordo orrido	t. st	They are eaten cooled down and seasoned with oil and salt.	The tender stems are eaten in Sardinia raw in salads, stewed with onion and tomatoes sauce, fried in olive oil whit tender leaves of *Foeniculum vulgare* Miller subsp. *vulgare* as condiment for pasta [26].
Onopordum illyricum L. @ (*Asteraceae*) *Onopordo maggiore*	t. st	They are eaten cooled down and seasoned with oil and salt; with flour and fried; or battered with eggs.	In Sardinia it is eaten raw or boiled as condiment for roast meat, it is also preserved in olive oil and winegar [26].<br>The roots of "cardonē" are eaten in Castelmezzano (Basilicata) boiled and fried [28]. In Turkey (Bodrum area) the top part of the root and the bottom leaves are collected, the spines and green parts are eliminated and the large central veins are left, boiled and filled with rice, onions and spices, cooked in a pan and after dipped in a mixture of flour, eggs and water and then fried in hot olive oil [29].
Opuntia ficus indica (L.) Miller @ *(Cactaceae*) Fico d'India (*)	fr	It is eaten as a fruit.<br>With the pulp of the fruits without seeds it is prepared a sauce used for pasta or for sweets or a typical pastry called "pizzichinti"; with the rind of ripe fruit, well cleaned from the thorns, are prepared delicious pancakes.	Fruits are eaten in Turkey [29].<br>In California the fruits and the young cladods are consumed (after having removed the thorns), cut, roasted and seasoned with oil, salt and lemon or fried in butter with cheese [59].

		The fruits with the seeds are eaten for the treatment of diarrhoea, while the fruit without the seeds are used against constipation.	
Origanum heracleoticum L. @ *(Lamiaceae)* <u>Origano meridionale</u> (*) (§)	le	The leaves are used to season meat, fish or salads and to prepare a typical condiment used together with onions and goat cheese, oregano and anchovies to bake a traditional pizza of the Trapani province called "*rianata*". It is considered a medicine-food because it helps with the digestion.	It is an aromatic herb with a sweet flavour and it is used for soups, broths and stuffing as described by Pliny [25]. The Albanian ethnical group Arbëreshë living in Basilicata uses the leaves as condiment [27] as also in the same region the inhabitants of Castelmezzano do. They call the leaves "*riganē*" [28]. The Bedouins use it in order to preserve the butter [31].
Origanum majorana L. @ *(Lamiaceae)* <u>Origano maggiorana</u> (*)	le	The leaves are used to season meat, fish or salads. It is considered a medicine-food because it helps with the digestion.	In Sardinia and in the Elba island the leaves are used as seasoning for food and as digestive herb [26, 46]. In Latium it is used to prepare ragouts, sauces, fish-dishes, maccheroni, meat balls and "*trippa*" (tripe) [43]. In Cyprus the "*mandzourana*" is used as seasoning and as spice with carminative and aphrodisiac properties [42]. The same use is known in Turkey where it is drunk as tea [32]. This herb was used as flavouring agent by ancient population of the Mediterranean basin [25]. In Tunisia the "*mardkouch*" is used as spice [31, 40].
Origanum vulgare L. @ *(Lamiaceae)* <u>Origano comune</u> (*)	le	The leaves are used to season meat, fish or salads. It is considered a medicine-food because it helps with the digestion.	In Sardinia and in Tuscany the leaves are used as condiment and to aromatize sauces [26, 36, 37] and also as food-medicine for their digestive action [44]. In Spain flowered aerial parts of "*oregano, orenga, ourego, oriénganu*" are used for making an herbal tea or the liqueur "*ratafia*" and for seasoning pork, olives, roasted meat [30].
Oryzopsis miliacea (L.)Ash. et Schweinf (*Poaceae*) <u>Miglio multifloro</u>	se	In Licata, in south Sicily, they are considered a very good food for pregnant women.	
Oxalis pes-caprae L. @ (*Oxalidaceae*) <u>Acetosella gialla</u>	wp	The leaves are eaten raw with salads. The bulbs called "*capulasisa*" are eaten grilled and seasoned with oil and lemon. The juice, bitter and at the same time sweet of the "*caracitula*" is appreciated as refreshing in the Agrigento province.	In Cyprus the "*oxaliss*" is used to prepare a traditional dish called "*spanakopites*" a triangular cake filled with *epinard,* oxalis leaves, and onions mixed with lemon juice, olive oil and salt. The leaves are also used to prepare soups and salads to which they confer a bitter taste [42]. It is a wild species eaten also in Salento [47], Sardinia [26], Spain [30] and in Turkey [29].
Papaver rhoeas L. subsp. *rhoeas* @ (*Papaveraceae*)	se le	In some villages of the Agrigento province it is traditional to spread the poppy seeds on the bread and on some sweets offered to the	In Tuscany, the boiled leaves are used to prepare delicious soups used as purifiers [35, 36, 37, 45] but only after being accurately boiled [56]. The Albanian ethnical group Arbëreshë living in Basilicata eats the whole

Papavero comune<br>Rosolaccio		bride in order to stimulate the fertility and bring them happiness.<br>The boiled leaves seasoned with oil are eaten in soups.	plant boiled and fried or in special soup with beans called "*luljekuq ma fazulje*" [27].<br>The tender leaves are eaten in Sardinia, Corsica and Latium in salads, raw, boiled, fried, in soup with the asparagus while the small seeds were used to season and decorate "*focacce*" and biscuits [26, 43]. It is a wild species eaten also in Salento [47].<br>Also in Crete [32], Spain [30] and in Turkey [29] the leaves of this plant are consumed as food. The Cypriots use the petals of the "*paparouna*" to paint with red the eggs during Eastern [42].
Papaver setigerum DC.<br>(*Papaveraceae*)<br>Papavero setoloso	se	The seeds are spread on the bread.	
Petroselinum sativum Hoffm. @<br>(*Apiaceae*)<br>Prezzemolo comune	le	It is used as seasoning for many dishes.	This edible plants it is used in many countries of the world [24] i<br>In Sardinia it is widely used for making cake, bread, as seasoning for different typical dishes [26]. In Latium the leaves used to be utilised in order to increase the milk quantity of the breastfeeding by putting them in soup with wild fencil and other vegetables [43]. The Albanian ethnical group Arbëreshë living in Basilicata eats the leaves raw or cooked as condiment [27].<br>Also in Tunisia the leaves of "*maadnous*" [31, 40] and in Turkey the leaves of "*gelincik*" [38] are used as seasoning.
Phagnalon saxatile (L.) Cass.<br>(*Asteraceae*)<br>Scuderi angustifoglio	le	It is used as seasoning for meat and fish dishes and also for potatoes.	
Phlomis fruticosa L.<br>*(Lamiaceae)*<br>Salvione giallo	le<br>fl	The leaves are used to aromatise meat; browned in butter or in lard they make an excellent pasta seasoning.<br>In the olden days, children used to suck the flowers due to their sweet taste.	In Turkey the leaves are used as condiment or spice [29].
Picris echioides L. @<br>(*Asteraceae*)<br>Aspraggine volgare	t. wp	It is eaten raw, seasoned with oil and salt.	In Tuscany and in Sardinia the leaves of the "*radicchio peloso*" are eaten boiled, alone or with other soups [35, 36, 37].<br>In Latium the tenderest basal leaves are eaten steamed, in turnip soups and browned in the frying pan, they are basic ingredients for vegetable pizzas [43]. The leaves of *Hyoseris radiata* e *P. echioides* are used in Liguria in the promontory of Caprione [48]. as a base for the preparation of "*torta d'erbi amai*".<br>The ethnic Albanian group Arbëreshë that lives in Basilicata eats it boiled and fried [27], the same use has been registered in the same region, in the village of Castelmezzano, where the plant is called "*spuscēnē*" [28].<br>In Spain the basal leaves of "*llengua de bou*" are eaten well stewed [30].
Pinus pinea L. @	se	They are used for the preparation of both	Pines have been used for centuries, since the Mediterranean has been

(*Pinaceae)*<br>Pino domestico		salty (pasta with sardines, meat-balls with sardines, "*pesto*") and sweets dishes.	populated. The young shoots and the male inflorescence were then eaten as vegetables, similarly to the seeds, which are rich in lipids and sugars [25]. In Sardinia, Spain, Turkey the ripe seeds are eaten raw , dried or as a condiment [26, 30, 29].
Pistacia vera L. @<br>(*Anacardiaceae*)<br>Pistacchio	se	They are used as dried fruits and for the preparation of salty dishes, to aromatise pork products, cheeses and also for pastries.	In Sardinia the dried fruits are widely used for making cakes and pastries [26]. In Cyprus the seeds of the "*pistakya*", when roasted and salted, are used as food and for the production of edible oil. The pistachios, when mixed with honey, are used as an aphrodisiacal sweet and a stimulant of virility [42]. In Tunisia, the fruits and the oil of the pistachio are called "*fousdouk*" and are used as food [40].
Pisum sativum L. subsp. *sativum*<br>(*Fabaceae*)<br>Pisello	se	The seeds are eaten boiled and seasoned with oil, salt and pepper; in an omelette; stewed with onions and tomatoes; browned in a frying pan with onion, parsley and oil.	In Cyprus the seeds are called "*biselli*" and if mixed with lupin they are used as medicinal food with the effect of lowering blood pressure [42]. In Sardinia the fresh or dried seeds are eaten boiled, stewed, browned in olive oil with onion and used as condiment for meat, eggs and fishes [26].
Plantago lagopus L.<br>(*Plantaginaceae*)<br>Piantaggine piede di lepre	br	It is eaten boiled and seasoned with oil and salt.	Different species of *Plantago* have been making part of the human diet for millions of years [49].<br>This herb is considered a refreshing type of food both in Crete [32].
Plantago serraria L<br>(*Plantaginaceae*)<br>Piantaggine seghettata	le	They are eaten boiled, drained and browned in garlic and oil, adding bread-crumbs and a drop of vinegar.	
Portulaca oleracea L. @<br>(Portulacaceae)<br>Porcellana comune	t. wp	It is used to prepare salads with tomatoes, capers and cucumbers or as an ingredient for delicious soups.	Dioscorides used to recommend it for its refreshing power against stomach inflammations.<br>This plant has been cultivated since ancient times in England, France, Italy, in Brazil and Central America, and was a very useful food for a ship crew suffering from scurvy [25].<br>The tender plant is added in mixed salads, due to its refreshing effect, diuretic and detoxicant. In Ciociaria it is used as an ingredient for anchovy salads and the seeds are used as an energetic bird-food [43]. The ethnic Albanian group Arbëreshë that lives in Basilicata uses the leaves raw or boiled in soups [27]. A similar use has been registered in the same region, in the village of Castelmezzano where the plant is called "*perchiazzē*" [28] and in Sardinia [26].<br>Alimentary plant of extensive use in Tunisia, also used as a curative food of a refreshing and antinflammatory effect [31].<br>It is eaten raw, used as a pot herb or browned with eggs in Yemen, Central Asia, Burma [25], Cyprus [42] Crete [32], Britain [53], Turkey [29, 38], Spain [30] where the tender parts are used as diuretic medicine food [33].
Prasium majus L.<br>(*Lamiaceae*)	le	Used for preparing drinks with a diuretic and thirst-quenching effect in Chiusa Sclafani,	The leaves are also used in Tunisia [31].

Thè siciliano		Favignana, Riserva dello Zingaro.	
Prunus dulcis (Miller) D. A Webb. @ (*Rosaceae*) Mandorlo	se	With the seeds: pounded with garlic, oil, tomatoes, basil, parsley and mature cheese, an exquisite "*pesto alla trapanese*" can be prepared which is used as a pasta seasoning; Grounded with garlic and parsley, they are used to aromatise fish cous cous; instead, when caramelised, they can be used to prepare the sweet made out of nougat and macaroon: "*la cubaida*". By peeling off the external skin and grounding it with water and sugar, the dish of "pasta with almonds" can be prepared, the chief ingredients for traditional sweets, particularly the fruits sold during the Celebration of the Dead, which are called "*frutta di marturana*". The seeds of the "*miennule*", when put together with those of the broad beans, peas and "*cocuzze*"(pumpkins), toasted and salted they make the "*kalia e simenza*" which is sold by pedlars during local celebrations. In addition, with the seeds, a refreshing and nutritious drink "*latte di mandorla*" is prepared, which is usually drunk in the summer.	In Chieti (Abruzzo) "*almond milk*" is prepared from the minced, crushed seeds mixed with water and sugar [50]. The almond, which in Cyprus is called "*amyghdalya*", is used both as a fruit and in order to extract an edible oil. The village inhabitants also prepare almond milk' which is called "*soumadha*", and which has to be diluted in water. It can then be used in confectioners' shops [42]. Almonds, when mixed with sugar and honey, are widely used to prepare sweets in all Mediterranean countries [26]. The kernels of "*menēla*" are eaten raw or dried and are used to aromatise traditional dishes of Basilicata such as "*sanghēnaccē*" and "*frisellē*" [28].
Prunus spinosa L. @ (*Rosaceae*) Pruno selvatico	fr	They are eaten during the autumn.	With the fruits of "*strozzapreti, prignole*" are prepared in Tuscany jams and also liqueurs they are used also as medicinal food which helps with digestion and works against diarrhoea [35, 36, 37, 44]. This wild edible species is used in Salento [47] and also in Latium where aromatic liqueurs, which help with digestion and stomach disturbances, are prepared [43]. The ethnic Albanian group Arbëreshë which lives in Basilicata, uses the cooked fruit in order to prepare jam and also raw as a snack [27]. A similar use of the fruit has been registered in the same region in the village of Castelmezzano where it is called "*trignonē*" [28]. In Corsica the fruits are used for making a liqueur [26]. The fruits are eaten in some regions of northern Europe and with sugar they are used to make a very good jam. The leaves are used to makea tea in France where the unripe fruits are used as a substitute for olives; while in Germany, Russia [25] and Spain [30] the crushed and fermented fruits are used to make a good liqueur.
Punica granatum L. @	fr	The pulp that covers the pomegranate seeds is	In Sardinia the fresh juice was used as seasoning for salads, meat and

(*Punicaceae*)<br>Melograno		used as a fruit or a freshly squeezed juice. By adding sugar and boiling it, a syrup known as "pomegranate syrup" is prepared.	fishes, today it is also used as refreshing juice [26]<br>The ethnic Albanian group Arbëreshë which lives in Basilicata uses the fruits boiled and added to wheat and beans, in a grape juice [27]. Precious food and seasoning, it is used in Tunisia [31, 40] and Turkey [29] as a fruit and for the preparation of a sweet syrup. In Palestine it has been used since ancient times for its digestive effect [59]. In Cyprus, the "*rodhya*" is used as a fruit and its juice, apart from being used as a refreshing drink; it is administered in cases of diarrhoea and parasites in the intestines.<br>The Cypriots consider the plant a symbol of fertility. The rituals of their tradition comprise of throwing a mature grenadine fruit on the floor, shortly before the newly wed couple enter their nuptial home, in order to bring luck [42].
Pyrus amygdaliformis Vill. @<br>(*Rosaceae*)<br>Pero mandolino	fr	They are eaten as fresh fruits.	The fruits of *P. amygadliformis* Vill. var. *amygadliformis* are eaten also in Turkey [29] and in Sardinia [26].
Raphanus raphanistrum L. @<br>(*Brassicaceae*)<br>Ravanello selvatico	yb<br>t. st	The young shoots are eaten salted, in the pan with garlic and peppers, or the tender stalk is eaten raw as a side dish with sausages. "*A razza nun fà cavuliceddu*" it is said of people who have no hope of refining themselves. Even if the "*razza*" (race) is similar, the taste cannot be compared with "*cavulideddu*" (*Brassica fruticulosa).*	The leaves of "gramolaccio" are used in vegetables soups in Sardinia [26], Tuscany [36, 37] as also in Spain [30] and in Turkey [29]. In Britain and northern Europe the young leaves and shoots (best collected before flowering time) are served finely chopped to give a piquant flavour to salads, cottage cheese, paté, omelettes and sandwiches. Larger quantities can be served as a soup with onions, lemon juice and season according to taste, the flower buds are used like broccoli, the seeds can be used to mak mustard [53].
Raphanus raphanistrum L. subsp. *landra* (Moretti) Bonnier @<br>(*Brassicaceae*)<br>Ravanello selvatico<br>(*)	ap	They are eaten boiled and seasoned with oil and lemon; in soups together with other vegetables; stewed in tomato sauces and in soups.<br>It is considered a medicinal-food and is used in Aragona for its digestive effect.	It is eaten as a vegetable in Cyprus, where it is called"*Raphanis*" [42], and also in Tunisia where it is known by the name of "*fjil el jabi*" [40]. In Latium and in Tuscany, the tender plant is eaten in salads or, alternatively, cooked in soups [43, 35].<br>In the Hebrides, its leaves are eaten as a salad [25], while in England, are sold the seeds as a substitute for mustard due to their aromatic and pungent aroma [25].
Raphanus raphanistrum L. subsp. *raphanistrum*<br>*(Brassicaceae*)<br>Ravanello selvatico	le<br>yb	The tender shoots or buds are eaten raw with salt like radish.<br>The top, "*spicuneddi*", and the leaves, when fried or boiled, are eaten as a side dish with meat dishes.<br>Fried with oil, garlic and peppers they are used as pasta seasoning.	
Rapistrum rugosum (L.) All.<br>(*Brassicaceae*)<br>Miagro peloso	ap	They are eaten cooled down and fried with peas and onions; boiled and seasoned with oil and lemon. Added to tomato sauces for	In Spain the leaves of this wild edible plant are consumed raw in salads or stewed [30].

		the preparation of "*u sucu cu a mazzaredda*" excellent as a pasta seasoning.	
Reichardia picroides (L.) Roth. @<br>(*Asteraceae*)<br>Grattalingua comune<br>(*)	br	It is eaten both raw and cooked, on its own or "*maritata*" (married) to other vegetables"cardedda *(Sonchus sp.),* cicoina *(Urospermum dalechampii),* scursunara *(Lactuca viminea)* and cutulidda *(Chondrilla juncea)*" and seasoned with oil and lemon.<br>Considered a medicinal-food, it is eaten for its refreshing, emollient and diuretic effect and is used in Canicattì, Campobello di Licata and Ravanusa.	The young aerial parts are eaten in Tuscany raw in salads or boiled and eaten as depurative food [44] or boiled in a mixture with other herbs and also in soups [35, 36, 37, 45] as well as the ethnic Albanian group Arbëreshë which lives in Basilicata [27]. A similar use has been registered in the same region in the village of Castelmezzano where it is called "*panē grizzitiddē*" [28].<br>The rosettes of the young plant, which were harvested in Spring and Autumn, are used in Sardinia and in Latium in salads or together with other vegetables in "*misticanza*" [26, 43]. In Brindisi it is used as a component of cooked, mixed vegetables [54].<br>In Cyprus, the leaves and roots of "*picridha*" are used in salads, boiled, in soups and with other vegetables [42]. Even in Crete [32] and in Spain, the plant is considered as food [30].
Ridolfia segetum Moris<br>(*Apiaceae*)<br>Aneto puzzolente<br>(*)	ap	They are eaten raw in salads and as medicinal-food in cases of gastric acidity, in Sant'Angelo Muxaro.	
Rosa canina L. @<br>(*Rosaceae*)<br>Rosa selvatica comune	psfr	It is eaten as a fruit.	The pseudo-fruits are eaten as a snack in Sardinia [26] and in Tuscany where fruits are used to make a jam [36, 37] and as medicinal food for its refreshing action [44]. The pseudo-fruits of "*scaddapoddēcē*" are eaten raw as a snack in the village of Castelmezzano (Basilicata) [28]. In Spain, the fruits of "tapaculos, escaramujo" are eaten raw as snack and are used for making liqueur [30], but the Spanish traditional medicine recommends using the fresh fruits for treatment of cold, diarrhoea and anaemia [33].
Rosmarinus officinalis L. @<br>*(Lamiaceae)*<br>Rosmarino<br>(*)	le	The leaves are used to aromatise meat, potatoes, bread and focacce like the "*vucciddati*" that are prepared on the 19th of March in the honour of Saint Joseph.<br>Considered medicinal-food, used for its aromatic properties which assist with digestion.	It was described by Pliny, Galen, and Dioscorides for its many medicinal virtues, but the first time that it was mentioned as a seasoning is by Lignamine in 1475, who described rosemary as the usual seasoning with meat [25]. The leaves are used as seasoning and as medicinal food in Tuscany [44] and also by the etnic Albanian group, Arbëreshë, that lives in Basilicata [27].<br>In Tunisia "*aklil*" is used as a spice in the kitchen in order to avoid that butter turns rancid and in addition as food which assists with stomach disturbances and is also carminative [40].<br>In Sardinia [28], Latium [43] and Puglia [54] it is used as a type of spice for food which is difficult to digest, with potatoes and roast meat. In Spain the leaves of "romero, romer, roman" are used for making liqueurs, as a preservative for meat or fish, as a condiment for olives, roasted meat and different stews [30]. It is also used in Turkey as

			condiment and medicine food [29]. In Cyprus it is called "*lasmari*" and is used as a carminative and an aperitive. It can also assist with gastric cramps. In the kitchen, a seasoning can be prepared with a basis of vinegar, rosmarin and coriander, which is then added to fried fish [42].
Rubia peregrina L.<br>(*Rubiaceae*)<br>Robbia selvatica	fr	They are eaten fresh or as a jam and also to prepare a delicate rosolio.	In Morocco it is used as aphrodisiac drink [31].
Rubus ulmifolius Schott. @<br>(*Rosaceae*)<br>Rovo comune	fr<br>le	They are eaten fresh or as a jam.<br>In Licata, a liqueur can be prepared by using the "*rivettu*" leaves.	In Sardinia [26] and in Tuscany the fruits are eaten raw or in a jam and the young shoots are eaten boiled or in omelettes [36, 44] and constitute a type of medicinal-food used as a remedy against cough [44]. In the village of Castelmezzano in Basilicata, the fruits called "*ciuz*" *(dē rēvētalē)* are eaten raw as a snack [28]. In Spain the fruits are eaten raw, cooked in jam or used for making liqueur, while the flowers are eaten as snack. [30, 33].<br>In Tunisia the fruits and the syrup of "*zinebit*" are eaten with honey [31].
Rumex crispus L. @<br>(*Polygonaceae*)<br>Romice crespo<br>(*)	ap	The tender aerial parts are used, boiled and seasoned with oil and lemon. Detoxifying medicinal-food.	In Sardinia and Tuscany the leaves are eaten boiled, in soups, on their own or with other vegetables [26, 35, 36, 37, 44]<br>The leaves are extremely rich in salts; therefore it is advisable that particular attention is paid, when consuming the leaves, by people who suffer from kidney stones [56].<br>This plant is called "*kazan kulpu*" and is eaten in Turkey [38] and Crete [32]. The basal leaves of "vinagrera" are a traditional ingredient in Spain for making stewed, soups, sauces and as a condiment [30]. It is used also in this country as medicinal food for cold, diarrhoea and digestive disorders treatment [33].
Rumex scutatus L.<br>(*Polygonaceae*)<br>Romice scudato	ap	It is chewed in order to extract the thirst-quenching juice.<br>It is also used to give flavour to salads.	The tender leaves and stems raw of "acedera agrietas" are used as a snack or in salads in Spain [30].
Rumex thyrsoides Desf.<br>(*Polygonaceae*)<br>Romice a farfalla	ap	The tender aerial parts are used, boiled and seasoned with oil and lemon	The tender aerial parts are eaten boiled also in Sardinia [26].
Ruscus aculeatus L. @<br>(*Liliaceae*)<br>Ruscolo pungitopo	tu	The young shoots are eaten as an asparagus: boiled and seasoned with oil and lemon, scrambled with eggs and cheese or in an omelette.<br>A traditional Palermitan dish, called in dialect "*sparaci di scupazzi impurrazzati*", is prepared by wrapping the "*sparaci*" in the "*purrazzu*" leaves and by cooking them in tomato sauce.	The young shoots are eaten cooked like an asparagus in Sardinia, [26], Tuscany [56], Basilicata [28], Latium where they are considered a medicinal food with detoxifying and diuretic properties [43]. In Tunisia it is used as an asparagus and the seeds, when roasted, can substitute the coffee [31]. The young shoots of "*rusco*" are eaten in Spain [30] as also in Slovakia and Croatia [51] stewed, in omelettes or with scrambled eggs. The young shoots are eaten also in Turkey [29].

		The seeds are roasted and a substitute of coffee is prepared in Piazza Armerina.	
Ruscus hypoglossum L.<br>(*Liliaceae*)<br><u>Ruscolo maggiore</u>	tu	The young shoots are boiled and seasoned with oil and lemon, scrambled with eggs, in omelettes or as an asparagus cream.	
Ruscus hypophyllum L.<br>(*Liliaceae*)<br><u>Ruscolo maggiore</u>	tu	The young shoots are boiled and seasoned with oil and lemon, scrambled with eggs, in omelettes or as an asparagus cream.	
Salvia officinalis L. @<br>(*Lamiaceae*)<br><u>Salvia domestica</u><br>*($)*	le	The fresh or dry leaves are used to aromatise various main dishes like roasts and sauces.	This plant is one of the most important occupants of the herb garden since ancient times, used as spices and for domestic medicine [21].<br>Even the ethnic Albanian group Arbëreshë that lives in Basilicata use the leaves as a condiment [27]. Similarly, in Sardinia and in the upper central Lucca province (Tuscany) and, the leaves are used as a condiment and as medicinal-food for its digestive action [26, 44].<br>In Latium, it is used to aromatise meat and game. It is considered that the animals that eat the wild sage during pasture have a tender and tastier meat [43].
Salvia sclarea L. @<br>*(Lamiaceae)*<br><u>Salvia moscatella, sclarea</u>	le	The fresh or dry leaves are used to aromatise various main dishes.<br>Their decoction was used to soak wine barrels and to give the wine a nice aroma.	In Sardinia are used the aerial parts as aromatising for wine to obtain a "moscato" flavour. The fresh or dry leaves are used to aromatise breads, cakes, omelettes, roast meat, soups, "*focaccie*" [26]
Sambucus nigra L. @<br>(*Caprifoliaceae*)<br><u>Sambuco nero,</u><br><u>Sambuco comune</u>	fl<br>fr	Both the fresh fruits and the flowers are eaten; the latter iced or fried in butter, powdered with a coating of sugar.<br>In Caltanisetta, the dry flowers were eaten with bread as snack.	The fruits are eaten cooked or as a jam in the Amalfitan coast (Campania) [55], like in Liguria [48], in Latium [43] and in Tuscany [36, 44] as "cleansing food" which acts as a laxative.<br>In the upper valley of the Serchio river in Garfagnana the inflorescence of sambuco are eaten fried or as jam and syrups (37). In Latium [43] and in Tuscany [35], pancakes can be prepared, by using the flower powdered with sugar.<br>The same recipe is prepared in Cyprus with the flowers of the "*zamboukkoss*" [42], in Greece and in many other countries in Europe since ancient times [25].<br>In Greece, the unripe, raw fruits are used as medicinal-food against diarrhoea [57], while in Tunisia the juice extracted out of the ripe fruits, is used for its refreshing and laxative action [31]. In Abruzzo, it is drunk after having eaten, as a digestive, a type of wine in which the flowers have been soaked [50], while the fruits are used in Portugal for colouring the wine "Port" [25]. The flowers or fruits of saúco, are used for making herbal tea liqueur or other beverages, while the fruits are a good ingredient for making delicious jam [30].
Scolymus grandiflorus Desf. @<br>(*Asteraceae*)	le<br>t. st	They are eaten boiled and seasoned with oil and lemon; scrambled or in an omelette with	The stalks are eaten raw or boiled by the Arabs and the Egyptians [25]. In Tunisia, it is called "*guenaria*" and is used as a medicinal-food which

Cardogna maggiore		eggs, in batter or in a salad.	stimulates good liver function [40].
Scolymus hispanicus L. @ (*Asteraceae*) Cardogna comune (*)	t. st	It is eaten cooked or raw and seasoned with oil and lemon; fried in butter, scrambled with eggs. In Sant'Angelo Muxaro it is considered a medicinal food for its digestive action.	Theophrastus and Pliny said that this plant was used to be eaten as a vegetable since ancient times [25]. After removing the thorns, the tender stem can then be eaten cooked in soups in Latium [43] and Sardinia [26]. It can also be eaten raw in salads or boiled and also as a timbale "*verdhet*", by the ethnic Albanian group Arbëreshë that lives in Basilicata [27]. It is used in Tunisia as a delicious seasoning for vegetable couscous [31]. The leaves are eaten cooked or raw and they are also used in Cyprus [42], Turkey [29, 32] and Spain, where the raw flowers are used as a saffron substitute [30].
Scolymus maculatus L. @ (*Asteraceae*) Cardogna macchiata	t. st	It is eaten cooked or raw and seasoned with oil and lemon.	The young leaves have been eaten as spinach since ancient times in Greece [25] and in Spain [30].
Silene vulgaris (Moench.) Garcke@ (*Caryophyllaceae*) Bubbolini	yb	Cooled down, chopped up and mixed with eggs and cheese, they are used to prepare the exquisite meat-balls with a "*nannata*". '(fried fish) taste; boiled in a soup with other vegetables or browned. Also used to aromatise omelettes.	In the upper central Lucca province, the young shoots and leaves of "erba striscia" are eaten boiled in a soup with other vegetables and they are also used as a stuffing for tortellini [36, 37] and the cooked leaves are also used as medicinal food for "cleansing" the organism from impurities [44]. It is also eaten stewed or raw in salads in Spain [30] and in Turkey [29] and raw or boiled in soup in Sardinia and as filling for pasta and cakes in Corsica [26].
Silene vulgaris (Moench.) Garcke subsp. *angustifolia* (Miller) Hayek (*Caryophyllaceae*) Silene rigonfia, Bubbolini, Strigoli	yb	The tender "*spicuneddi*", harvested in Spring before full bloom, are eaten boiled in "*misticanza*" with other vegetables or, alternatively, cooled down and scrambled with eggs. Boiled, mashed and mixed with eggs and cheese, pecorino cheese and black pepper are used to prepare delicious meat balls.	In Garfagnana the young leaves of "*erba striscia*" are eaten in soups, boiled and browned in a frying pan [35], while in Latium the young, fresh shoots of the plant are eaten in soups and in omelettes [43]. The same use has been registered in Puglia [54].
Silybum marianum L. @ (*Asteraceae*) Cardo di S. Maria, Cardo mariano	ap	They are eaten raw in salads, battered, stewed like the artichokes, fried with garlic and chili pepper tomato and "*tuma*" (goat cheese); cooked in the oven with garlic, oil and crumbled-bread. In Campobello di Licata the peaseants used to suck the stem in order to quench their thirst during the working hours.	In Cyprus it is called "*Silyvon*" and it is used by the inhabitants like food since ancient times [42]. In England, Scotland, Italy [25] and Tunisia [31] the young leaves are used in spring salads or boiled as a substitute of spinach, the stalk is peeled, cooked and fried, while the receptacle of the flowers are cooked and eaten as artichokes. The Albanian ethnical group Arbëreshë living in Basilicata uses the whole plant boiled and fried [27], in the same region, in the village of Castelmezzano, the tender stem of "*cardeddē/cardonē spicchialicchiē*" is eaten raw as a snack [28]. In Sardinia [26] and in Spain [30] basal leaves, young shoots or tender part of inflorescence are eaten raw or stewed and are use in this last country as a food medicine as circulatory stimulant [33].
Sinapis alba L. @	ap	They are eaten like vegetables, boiled with oil	In Tunisia "*chenaf el khadad lift*" it is used like seasoning and laxative

(*Brassicaceae*)<br>Senape bianca<br>(+)		and lemon. This plant is traditionally considered an "*cauru*" (hot) food and an excesive use it is not recommended because it could cause stomach pains and diarrhoea.	food [40]. This species called "*sinapiss to lefko*" is used in Cyprus to prepare a condiment for a typical cipriot dish called "*moungrac chenaf el khadal lift*" [42]. This is a edible plant also in Spain [30] and in Sardinia [26].
Sinapis arvensis L. @<br>(*Brassicaceae*)<br>Senape selvatica<br>(+)	ap	They are also eaten like vegetable soup seasoned with oil and lemon; like side dish for sausages after being fried in the pan with garlic, oil and chilli pepper.<br>The elderly suggest not eating big quantities of it because it is a "*cauru*" (hot) food. A popolar proverb of Raffadali says: "*alassani tastali e lassali*" (try it and leave it).	In Sardinia and also in Turkey this wild edible plant is eaten as grens [26, 29]. The Cypriots eat the young leaves in salads or boiled and seasoned with oil and lemon as medicine-food against constipation [42]. The Albanian ethnical group Arbëreshë living in Basilicata uses the aerial parts boiled and fried to prepare a special dish during the Christmas when this herb is cooked with stockfish [27], in the same region, in the village of Castelmezzano the roots and the aerial parts of the plant called "*làssan[illegible]*" are eaten boiled or fried [28].
Sisymbrium irio L.<br>(*Brassicaceae*)<br>Erba cornacchia irida	Le	They are eaten raw in salad or boiled and seasoned with oil, lemon and salt.	The leaves are eaten in Sardinia raw in salad, boiled in soup, browned with oil, garlic and onion as condiment for roast meat [26].
Sisymbrium officinale<br>(L) Scop. @<br>(*Brassicaceae*)<br>Erba cornacchia commune<br>(+)	inf	The inflorescences called "*sciuriti*" are eaten boiled and seasoned with oil and lemon, in soups, battered, or fried with oil and garlic. The elderly suggest not eating big quantities of the plant because it is a food "*cauru*" (hot).	Used for its garlic aroma since the ancient time in Europe and North Africa now it is naturalised in the United States, and used to season sauces and salads and as a pot herb [25].<br>In Tuscany the leaves of "*sportavecchia*" [35, 36, 37] "*erbo nero, rape selvatiche*" are eaten boiled together with other vegetables as well as in Latium [43].<br>In Cyprus it is considered to be an aphrodisiac food [42].
Smilax aspera L. @<br>(*Smilacaceae*)<br>Salsapariglia nostrana	yb	They are eaten after being "*curati*" ("washed") and seasoned with oil and lemon; also used like ingredient for omelettes.	In Puglia [54] as well as in Turkey [29, 32] the young aerial edges of the plant are used like the asparagus boiled and seasoned with oil and lemon or also scrumbled with the eggs. In Spain the root of "*zarzaparrilla*" is used for making a beverage, while young shoots are eaten raw as snack [30].
Smyrnium olusatrum L. @<br>(*Apiaceae*)<br>Corinoli comune	Yb<br>fl. b	They are eaten raw in salad and like seasoning for soups as substitute for celery.	In Sardinia [26], Turkey [29]. and Spain [30] the leaves are eaten as greens. The "*smyrnyon*" is used in Cyprus like medicine-food added to the food because of its antiseptic, stomachic, carminative and tonic properties [42].
Sonchus asper L. @<br>(*Asteraceae*)<br>Grespino spinoso	ap	They are eaten in soup or raw in salad seasoned with oil and lemon.	In Sardinia, Corsica and Tuscany, the leaves of "*cicerbica*, *crescino*" are eaten raw in salad or boiled in soup with other vegetables "*erbi*" [26, 36, 37] and are used as medicine-food in order to clean the intestine [44].<br>The leaves of the various species of *Sonchus* are normally eaten in Salento [47], by the Albanian ethnical group Arbëreshë living in Basilicata [27], always in the same region in the village of Castelmezzano [28] and in Latium where they are also eaten in soups with turnip and other wild herbs or used as ingredient in vegetarian pizzas [43].<br>In Spain [30] Turkey [29] and Crete [32] the leaves are appreciated like food as well as in Cyprus where the "*sokkhos*" is considered to be a

			medicine-food with laxative properties [42].
Sonchus asper (L.) Hill subsp. *nymanii* (Tineo et Guss.) Hegi (*Asteraceae*) <u>Grespino spinoso</u>	ap	They are eaten in soup or raw in salad seasoned with oil and lemon.	Laeves are eaten in upper central Lucca province (Tuscany) raw salads or boiled in soup with other vegetables, it is also used as medicinal-food, for depurative and intestine cleansing [44].
Sonchus oleraceus L. @ (*Asteraceae*) <u>Grespino comune</u> (*)	ap	They are eaten in soup or raw in salad, boiled and fried in the pan like seasoning for tasty omelettes. In Caltanisetta, Vittoria and Partanna, the leaves boiled and the water in which they have been boiled, are considered medicine-food in Vittoria because of their laxative, refreshing and diuretic properties.	Described as food by Dioscorides, Pliny wrote that Hecate prepared a dish with this plant for Theseus, before he went into the labyrinth [25]. In Tuscany the leaves of "*cicerbica*, *crescino, cardelini*, *scargiglioni*"" are eaten in salads, boiled or in soups together with other vegetables "*erbi*" [35, 37, 45]. The same happens beside the Albanian ethnical group Arbëreshë that live in Basilicata [27], in Puglia [47] and in Liguria" [48]. The young leaves are put into tasty salads and soup in Egypt, Germany, New Zeeland [25], Spain [30, 33], Tunisia [31], Crete [32], Cyprus [42].
Sonchus tenerrimus L. @ (*Asteraceae*) <u>Grespino sfrangiato</u> (*)	ap	They are eaten in soups or raw in salad seasoned with oil. The leaves boiled and the water in which they have been boiled, are considered medicine-food in Vittoria because of their laxative properties.	The leaves are eaten raw in salad or boiled in soups in Corsica, Sardinia [26], Spain [30], Tunisia [31] and Cyprus [42].
Sorbus domestica L. @ (*Rosaceae*) <u>Sorbo comune</u> (*)	Fr	The fruits can be eaten in case of diarrhoea because of their astringent properties. It is normally eaten like fruit.	The fruits of "*sorbo*" are eaten raw or used for preparing jam in Basilicata [28] and in Tuscany, where are also used in popular medicine, against diarrhoea [35, 44], similar use as medicine food is recorded in Sardinia [26] and in Spain [30, 33].
Tamus communis L. @ (*Dioscoreaceae*) <u>Tamaro, Cerasiola, Uva tamina, Vite nera , Viticella</u> (+)	yb	They are only eaten cooked (because they are toxic if eaten raw) seasoned with oil and lemon; browned with oil and onions to season omelettes and *risotti*; fried also with eggs and cheese; cooked with the tomato sauce.	The plant originates from Europe, Persia and Africa and was already used as food from Dioscoride's time. Its young shoots were only eaten boiled [25]. The tender leaves can be eaten both cooked and raw, in soups and omelettes. In Sardinia [26] and in Basilicata are eaten the young shoots boiled and then fried with eggs eats [27]. They are still eaten in Turkey [29], Spain [30], Crete [32], Cyprus [42], Slovakia and Croatia [51].
Tanacetum vulgare L. @ (*Asteraceae*) <u>Erba amara selvatica</u>	Le	They are used both as a spice and as a seasoning.	
Taraxacum officinale Weber@ (*Asteraceae*) <u>Tarassaco comune</u> (*)	ap	The fresh leaves are eaten raw or cooked, in omelettes or browned in a frying pan. The buds are kept in salt, like capers, and are used to flavour main courses. The boiled root, when seasoned with olive oil, becomes a tasty dish with a wonderful aroma. The plant is considered medicinal-food for its detox and laxative effect. It also provides protection for the liver.	The tender leaves of this plant, which in some regions of Tuscany it is called "*cicoria selvatica, radicchio selvatico, piscialletto, insalatina selvatica*" [34, 35, 36, 37, 44], are used raw in salads. boiled in soups with other vegetables or in omelettes in Sardinia [26], Abruzzo [50], Latium [43], Liguria [48] and in [27, 28] In Turkey [29] and in Greece [57] it is used as medicinal food in the cases of digestive difficulties. In Spain "*diente de leon*" is used as herbal tea, coffee substitute, snack or salad [30].
Teucrium fruticans L.	le	They are eaten boiled and seasoned with oil	

(*Lamiaceae*)<br>Camedrio femmina		and salt.	
Teucrium scordium L.<br>(*Lamiaceae*)<br>Camedrio Scordio	le	They are used to prepare aromatic wines, liqueurs, aperitifs and digestives.	
Thymus capitatus (L.) Hoffmanns. et Link@<br>(*Lamiaceae*)<br>Timo arbustivo	le	They are used to aromatise traditional focaccia bread, tinned olives and salads with sardines, fish and meat dishes.	In Tunisia "*zaateur*" [40] is used as an aromatic seasoning and also in order to enhance sexual potency [31]. In Cyprus, "*thymos*" is used as seasoning for cheeses, focaccia bread, olives and as an aromatic combustible fuel for grilled food and also as an excellent "mellifera" (out of which honey can be extracted) plant [42]. It is used in Turkey, when dried like tea, as a type of seasoning [32].
Thymus spinulosus Ten.<br>(*Lamiaceae*)<br>Timo spinosetto	le	They are used as aromatic spices.	
Tolpis virgata (Desf.) Bertol.<br>(*Asteraceae*)<br>Radicchio virgato	br	It is eaten after having been boiled and seasoned with oil.	
Tragopogon crocifolius L. @<br>(*Asteraceae*)<br>Barba di becco minore	ap	They are eaten boiled in soups or raw in salads, seasoned with oil and salt.	Pliny wrote about its alimentary use in Egypt [25].
Tragopogon porrifolius L. subsp. *australis* (Jordan) Br.-Bl. @<br>(*Asteraceae*)<br>Barba di becco violetta,	ap<br>ro	The root is eaten after having been boiled; the stem and tender leaves are eaten raw in salads or boiled.	The tender leaves and stems are eaten in Sardinia and in Spain raw as snack, in salads or stewed [26, 30].
Urospermum dalechampii (L.) Schmidt @<br>(*Asteraceae*)<br>Boccione maggiore	le	This vegetable is much appreciated in countrymen's culinary tradition, due to its bitter taste and the fleshy consistency of its leaves that are eaten boiled or seasoned with oil or browned with garlic, oil and peppers.	This vegetable is eaten raw or cooked and seasoned with oil and lemon in Sardinia [26], Latium [43], Tuscany [35, 37], Puglia [47] and Basilicata [28].
Urospermum picroides (L.) Schmitd.<br>(*Asteraceae*)<br>Boccione minore	le	They are eaten raw in soup or cooked in salads seasoned with oil, salt and pepper.	This plant is eaten in salads or boiled in Sardinia [26], Puglia [47]. Crete [32], Spain [30] and Cyprus [42].
Urtica membranacea Poiret<br>(*Urticaceae*)<br>Ortica membranosa	le	The leaves harvested before full bloom and cooked in boiling water, are eaten both on their own and "*maritate*" (married, together) with other vegetables, seasoned with oil and lemon or they are used for the preparation of risottos and soups.	The tender leaves and stems of "ortiga" are eaten in Spain raw in salads or stewed in omelettes [30] , while are used in Sardinia to make soups , timballe or, mixed with cheese, as filling for "ravioli" [26].

Urtica urens L. @<br>(*Urticaceae*)<br>Ortica minore	le	The leaves harvested before full bloom and cooked in boiling water, are eaten both on their own and "*maritate*" (= married, together) with other vegetables, seasoned with oil and lemon or they are used for the preparation of risottos and soups.	In some areas of Sardinia [26] the whole tender plant is used for the preparation of excellent omelettes, that often are prepared in Tuscany together with young shoots of *Clematis vitalba, Asparagus acutifolius, Rubus fruticosus* [45]. This wilfd plant it is also used in soups and as a stuffing for tortellini. This plants it is used as medicinal-food due to its intestine-cleansing effect [35, 44]. In Spain is eaten in salads or in omelettes [30].<br>In Cyprus, when the seeds are mixed with honey, they are eaten as an aphrodisiac sweet [42].
Valerianella eriocarpa Desf. @<br>(*Valerianaceae*)<br>Valerianella campanulata	wp	It is eaten raw in salads and seasoned with oil, salt and vinegar.	The tender leaves are added to the "*insalata campagnola*" (country salad) of Latium [43].
Veronica anagallis-aquatica L. @<br>(*Scrophulariaceae*)<br>Veronica acquatica	le	It is eaten raw in salads and seasoned with oil and salt.	In Sardinia [26], Spain [30] and Turkey [38], the plant is used for an alimentary purpose raw in salad or in soup; the same use has registered in Tunisia where it is recommended to control hunger [31]. In Cyprus, the tender leaves of "*veroniqui*" are eaten in salads or in soups, and constitute a purifying main course, refreshing and with a slight laxative effect [42].
Vitis vinifera L. @<br>(*Vitaceae*)<br>Vite comune	fr	It is eaten as a fruit and its fresh juice is drunk. If the must is let to cool down for at least 1 hour, a dense liquid, "*vinu cottu*" (cooked wine), is extracted and sugar which is used to aromatise the "*cuccìa*" (a traditional dish based on grain and cooked chick-peas, typical of the Saint Lucia celebration on the 13th of December), ragú (meat sauce) and "mustazzoli" (sweets made out of grain flour). The pips, when left to dry in the sun, are called "*passulina*" and are used to prepare many traditional, Sicilian dishes, both sweet and salty.	The ancient Egyptians used to make great wine with the use of this plant [25]. The fruits are widely used in Sardinia for making different types of traditional dishes [26].<br>In Abruzzo a "*vin cot*" is prepared by scalding the fresh "*must*" of *V. vinifera* until its volume decreases to 2/3, it is then drunk after meals as a digestive [50]. The ethnic Albanian group Arbëreshë that lives in Basilicata uses the fruit raw, as a condiment and to prepare wine and vinegar [27]. In the old days, in Latium the young shoots were eaten both raw and boiled, the leaves were instead used to wrap stuffed meat rolls. The pips, when dried up in the sun, "*uva passa*", eaten with bread constituted a meal for the poor citizens; the vine shoots are used to aromatise lamb, sausages and roasted artichokes [43].<br>The grapes, called "*aanab*" in Tunisia, are considered an excellent, nutritious and refreshing food [40].<br>The vine leaves, both fresh and tinned in salt or vinegar, are commonly used in Greece, Bulgaria and Romania for the preparation of meat rolls with a rice and meat stuffing [60]. In Spain the fruits of "*parreña, parra, soteña*" are eaten raw, are used for making beverages (wine and liqueur) or vinegar while the young peeled shoots are eaten raw as snack or prickled in brine [30]. In Turkey the fruits are eaten raw and the dried plant is used as a fuel [29].

The taxa with the symbol (@) are edible wild plants according to PFAF database (see article's text)
The taxa with the symbol (*) are used as medicinal food
The taxa with the symbol (+) are considered as irritants if consumed in excessive quantities
The taxa with the symbol (§) indicate that the same plant is used as food both by Arbëreshë Albanian ethnical group of the Basilicata and by the ethnical group based in Sicily
The underlined names refer to the common Italian botanical plant names according to *Flora d'Italia* of Pignatti [60]
Parts used abbreviation : ap, aerial part; br, basal rosette; bu, bulb; fl, flowers; fl. b, flower buds; fr, fruits; infl, inflorescence; le, leaves; mu, mushroom; ro, root/tuber; ps. fr,
pseudofruits; se, seeds; t. p, tender part; t. st, tender stem; st. j, stem juice; wp, whole plant; yb, young buds; ys, young shoots; tu; turions
