# Supplementary material for: Wild food plants of popular use in Sicily
Source: J Ethnobiol Ethnomed. 2007 Mar 30;3:15. doi: 10.1186/1746-4269-3-15 (PMC1858679; doi:10.1186/1746-4269-3-15)
Supplement: Additional file 2 — Sicilian vernacular names of food plants [file 1746-4269-3-15-S2.pdf]

Table 2. Sicilian vernacular names of the recorded wild food plants

Scientific name and botanical family	Sicilian vernacular name
Agave americana L. (*Agavaceae*)	**Zamara** (EN-Piazza Armerina)
Allium ampeloprasum L. (*Liliaceae*)	**Agghiastru** (CT-Randazzo, Bronte)<br>**Agghiu porru** (CT-Adrano, Milo, Linguaglossa, Nicolosi, Ragaina, Zafferana)<br>**Aggioru** (CT-Randazzo)<br>**Cipuddazzu** (CT-Nicolosi, Pedara)<br>**Ghiastru** (CT-Bronte)<br>**Porru** (AG-Montallegro, Realmonte)<br>**Puorru sarvaggiu** (RG-Vittoria, Ragusa)<br>**Purri** (EN-Piazza Armerina)<br>**Purrietti** (ME-Mistretta)<br>**Purru** (CL-Butera, Riesi)
Allium nigrum L. (*Liliaceae*)	**Porra** (AG-Cattolica Eraclea)
Allium roseum L. (*Liliaceae*)	**Porru** (AG-Canicattì, Campobello di Licata, Ravanusa)
Allium sativum L. (*Liliaceae*)	**Agghia** (AG, CL.CT.EN, ME, RG, SR, TP)
Allium schoenoprasum L. (*Liliaceae*)	**Erva cipuddrina** (AG-Montallegro, Realmonte)
Allium triquetrum L. (*Liliaceae*)	**Porrua** (AG-Sambuca di Sicilia)<br>**Pràs** (PA-Contessa Entellina)
Amaranthus retroflexus L. (*Amaranthaceae*)	**Lippia** (RG-Vittoria)
Ammi majus L. (*Apiaceae*)	**Sberra,Caliota** (CL-Butera)
Anthemis precox L. (*Asteraceae*)	**Cucumidda, Pani cavaddu** (AG-Licata)
Apium graveolens L. (*Apiaceae*)	**Accia** (CL-Santa Caterina Villermosa, Vallelunga Pratameno); (PA-Caccamo, Sclafani Bagni, Valledolmo, Ustica); (TP)
Apium nodiflorum L. (*Apiaceae*)	**Crisciuni** (AG-Bivona)<br>**Scavuna** (ME-Mistretta)
Arabis turrita L. (*Brassicaceae*)	**Mazareddra duci** (CL-Caltanissetta, San Cataldo, Serradifalco)
Arbutus unedo L. (*Ericaceae*)	**Corbezzulu** (PA-Cefalù)<br>**M'briacotti** (EN-Piazza Armerina)<br>**M'briacheddi** (ME-Filicudi, Vulcano)<br>**Acummaru** (ME-Panarea, Salina)
Asparagus acutifolius L. (*Liliaceae*)	**Asparaci** (AG-Licata)<br>**Sparaci** (Pa-Ustica)<br>**Sparaci finu** (TP- Valderice)<br>**Sparaci sarbaggiu** (AG- Montallegro, Realmonte)<br>**Sparaci servaggi** (ME-Messina, Mistretta, Santo Stefano di Camastra)<br>**Sparacia** (TP-Calatafimi, Campobello di Mazara Castelvetrano, Salemi, San Vito Lo Capo); (CT-Bronte)<br>**Sparaciara** (CT-Bronte, Randazzo)<br>**Sparaciara** (CT-Randazzo)<br>**Sparacieddru** (CL-Campofranco, Sutera)<br>**Sparacio** (SR -Pachino)<br>**Sparacio** (SR-Pachino)<br>**Sparaciu sarvaggiu** (TP-Riserva dello Zingaro)<br>**Sparaciu** (CL-Butera, Mazzarino, Riesi, Sommatino); (TP-Erice,

	Riserva dello Zingaro); (CT-Maletto, Randazzo)<br>**Sparaciù** (CT-Randazzo)<br>**Sparaciu** (RG-Vittoria); (AG- Burgio, Lucca Sicula, Sambuca di Sicilia, Villafranca Sicula)<br>**Sparaciu** (TP-Custonaci, Vita); (CT-Maletto)<br>**Sparaciu niuru** (TP-Vita); (CT-Nicolosi, Biancavilla, Ragaina, Belpasso); (AG-Lampedusa, Linosa)<br>**Sparaciu sarbaggiu** (CL- Caltanisetta, San Cataldo, Serradifalco)<br>**Sparaciu sarbaggiu** (CT-Castiglione)<br>**Sparaciu scuru** (CT-Ragaina)<br>**Sparaciu spinusu** (CT-Castiglione)<br>**Sparacogna** (CT- Acireale Catania)<br>**Sparacogna** (CT-Catania, Acireale, Pedara, S.Giovanni la Punta, Nicolosi, Ragaina, Zaffarana, Belpasso, Santa Venerina)<br>**Sparangiu** (EN-Piazza Armerina)<br>**Spariciu** (RG-Vittoria)<br>**Spina ri puci** (Tp-Erice, Gibellina , Levanzo)<br>**Spinapucciu** (CT-Linguaglossa, Castiglione)<br>**Spinapuggiu** (CT-Linguaglossa, Castiglione)<br>**Spinapulici** (CT-Milo, Giarre, Pedara, Nicolosi, S. Venerina, Zafferana)<br>**Spini du bamminu** (CT-Adrano)<br>**Spinipuggiu** (CT-Linguaglossa)
Asparagus albus L.<br>(*Liliaceae*)	**Sparaci** (PA-Ustica); (Tp-Favignana, Levanzo)<br>**Sparaciu spinosu** (TP-Riserva dello Zingaro)<br>**Sparaciu sarvaggiuu** (TP-Riserva dello Zingaro)<br>**Sparacogna** (CT-Adrano Ragaina)<br>**Sparaciu** (AG-Cattolica Eraclea)<br>**Sparaciu jancu** (CT- Belpasso, Bronte, Biancavilla)
Asparagus officinalis L.<br>(*Liliaceae*)	**Sparaciu** (AG-Bivona, Cammarata)<br>**Sparaciu manzu** (CT-Adrano, Bronte, Linguaglossa, Maletto, Randazzo, Zafferana)<br>**Sparaciu 'mpiriali** (CT-Castiglione, Nicolosi)<br>**Sparaciù di vigna** (CT-Zafferana)
Asphodeline lutea L.<br>(*Liliaceae*)	**Baffalupo** (TP-Riserva dello Zingaro)<br>**Baffaluchi** (ME- Messina Mistretta,)<br>**Battagghiori** (CT-Maletto)<br>**Garufi** (AG-Bivona)<br>**Garufu** (PA-Bisaquino)<br>**Musuluchi** (TP-Buseto Palizzolo, Campobello di Ma zara)<br>**Puddicina** (AG-Cianciana)<br>**Puddicini** (AG-Aragona, Sambuca di Sicilia)<br>**Puddicinu** (AG-Comitini, Raffadali, Sant'Angelo Muxaro, San Biagio Platani, Burgio, Lucca Sicula. Villafranca Sicula)<br>**Scannabbeccu** (CT-Pedara, Regaina)<br>**Scornabeccu** (AG-Montevago); (TP-Partanna); (CT-Nicolosi, Regaina Zafferana)<br>**U' puddicinu** (AG-Raffadali)<br>**Zzubbi** (CT-Adrano, Biancavilla, Bronte, Maletto, Randazzo)
Asphodelus microcarpus Salzm.et Viv.<br>(*Liliaceae*)	**Agghiu porru** (ME-Filicudi, Panarea, Vulcano)<br>**Agghiu porru, Purrazzu** (ME-Salina)
Atractylis gummifera L.<br>(*Asteraceae*)	**Masticogna** (ME); (CT-Bronte)
Barlia robertiana Loisel.<br>(*Orchidaceae*)	**Patatara, Peri di patatara** (CT-S. Pietro di Caltagirone)
Bellis perennis L.<br>(*Asteraceae*)	**Erva di primu ciuri** (CT-Linguaglossa)<br>**Erba bianca** (CT-Maletto)<br>**Jancuzzu** (CT-Nicolosi)<br>**Primusciuri** (CT-San Giovanni La Punta)
Beta vulgaris L. subsp. *maritima* (L.) Arcang.	**Aggiti** (RG-Vittoria)<br>**Agiri** (EN-Piazza Armerina)

(*Chenopodiaceae*)	**Biletti** (CL-S.Caterina Villermosa)<br>**Geri** (CT-Biancavilla)<br>**Gidi** (AG-Bivona, Cianciana)<br>**Gira** (TP-Buseto Palizzolo, Castellammare del Golfo. Custonaci, Favignana, San Vito lo Capo, Valderice, Riserva dello Zingaro); (CT-Adrano, Randazzo); (ME-Mistretta, Tortorici)<br>**Gira di marina** (TP-Favignana)<br>**Giri** (CT-Maletto, Bronte, Regaina); (AG-Sambuca di Sicilia, S. Caterina Villermosa, Isole Pelagie);<br>(PA-Ustica); (TP-Calatafimi, Campobello di Mazara, Gibellina, Salemi, Vita, Riserva dello Zingaro)<br>**Sarchi** (AG-Casteltermini, S. Giovanni Gemini)<br>**Secala** (CT-Nicolosi, Pedara, Zafferana, Linguaglossa, Castiglione, Belpasso)<br>**Secala sarbaggia** (CT- Milo, S.Giovanni la Punta, S.Venerina,)<br>**Seghila** (CT-Linguaglossa)<br>**Zarca** (Butera, Mazzarino, Riesi, Sommatino); (AG-Licata, Raffadali)<br>**Zarca sarbaggia** (AG-Siculiana)<br>**Zarchi** (ME-Santo Stefano di Camastra); (AG-Isole Pelagie)<br>**Zarchi sarbaggi** (AG-Campofranco, S. Angelo Muxaro)<br>**Zarchi sarvaggi** (AG-Cattolica Eraclea)<br>**Zarchitedda** (AG-Aragona)<br>**Zarchiteddi di campagna** (AG-Favara)<br>**Zarchiteddi di giri** (AG-Comitini)<br>**Zarchiteddra** (AG-Realmonte, Montallegro)
Beta vulgaris L. subsp.*vulgaris* (*Chenopodiaceae*)	**Gira sarvaggia** (TP-Favignana)<br>**Giri** (ME-Messina)<br>**Zalaca** (TP-Castelvetrano, Partanna, Poggio Reale, Salaparuta)<br>**Zarca** (AG-Raffadali)
Borago officinalis L. (*Boraginaceae*)	**Bburraina** (AG-Comitini, Favara , Bivona); (ME-Galati Mamertino, Longi, S.Marco di Alunzio, Santa Agata di Militello, Tortorici)<br>**Bburraina** (CT-Linguaglossa, Castiglione, Randazzo, Maletto Bronte)<br>**Burraina,Vurraina** (AG-Realmonte, Montallegro, Canicattì)<br>**Burraini** (AG-Bivona)<br>**Burrana** (AG-Burgio, Lucca Sicula, Villafranca Sicula)<br>**Burrania** (TP-Buseto Palizzoloto, Calatafimi,<br>Pantelleria); (PA-Bisaquino); (AG- Aragona, Cattolica Eraclea, Comitini, Favara, Raffadali, Sant'Angelo Muxaro, Siculiana, Montevago); (TP-Trapani)<br>**Burranii** (AG-Raffadali, Lampedusa), (Me-Santo Stefano di Camastra); (TP-Favignana, Levanzo, Marittimo)<br>**Urrania** (CT-Nicolosi, S.Giovanni la Punta, Pedara, Zafferana, Biancavilla, Regaina, Belpasso)<br>**Vurraiina** (RG-Vittoria)<br>**Vurraina** (ME-Mistretta, Isole Eolie) ; (CL-Caltanissetta); (AG-Sant'Angelo Muxaro); (Me- Messina), (PA-Ustica)<br>**Vurraini** (AG-Cammarata); (CL-Mussumeli)<br>**Vurrana** (TP-Riserva dello Zingaro)<br>**Vurrania** (AG-Licata, Casteltermini); (CT- Adrano , Milo, Nicolosi, S. Venerina, Zafferana); (RG-Vittoria) ; (TP-Calatafimi, Salemi, Vita, Zingaro); (ME-Tusa); (AG-Campobello di Licata, Canicattì, Ravanusa)
Brassica fruticulosa Cyr. (*Brassicaceae*)	**Caliceddu** (CT-Adrano)<br>**Caliceddu (**CT-Pedara, Biancavilla, Regaina)<br>**Caluceddu** (CT-Adrano, Belpasso, Nicolosi)<br>**Cauliceddu** (CT-S. Giovanni la Punta, Castiglione)<br>**Caulicellu di vigna** (CT-Maletto)<br>**Cauricellu** (CT- Bronte, Randazzo)<br>**Cavuliceddu** (CT-S. Venerina,)<br>**Coliceddu** (CT-Linguaglossa)<br>**Coluceddu** (CT-Adrano)

	**Coricellu** (CT-Bronte)<br>**Qualiceddu** (S.Venerina, Zafferana, Milo)<br>**Quaricellu** (CT-Maletto, Bronte)<br>**Rapuddi** (ME-Isole Eolie)
Brassica nigra (L.) Koch<br>(*Brassicaceae*)	**Cavuliceddu niuru, Qualeddu** (AG-Casteltermini)<br>**Lassani** (AG-Cianciana)<br>**Mazzaredda amara** (CL-Caltanissetta)<br>**Mazzareddi** (AG-Montevago)<br>**Qualedda** (CT-Giarre); (TP-Castelvetrano, Partanna)<br>**Senapa** (TP-Poggio Reale, Salaparuta)<br>**Sinàpi** (ME-Mistretta, Messina), (TP-Buseto Palizzolo, Castellammare del Golfo, Custonaci, San Vito lo Capo, Valderice, Trapani)<br>**Sinapu** (CL-Butera, Riesi)
Brassica rapa L. subsp. *sylvestris* (L). Janchen<br>(*Brassicaceae*)	**Alassanu sarbaggiu** (AG-Aragona, Realmonte)<br>**Cavulazzi, Sciuriddi, Alassanu sarbaggiu, Cavuliceddru amaru** (AG-Campobello di Licata, Canicattì, Ravanusa); (ME-Messina)<br>**Cavuledda** (AG-Sambuca di Sicilia)<br>**Cavuliceddi** (TP-Calatafimi, Gibellina, Salemi, Vita)<br>**Cavuliceddu amaru** (AG-Montallegro), (CT-Catania, Acireale); (CL-Caltanissetta, Serradifalco, S.Cataldo)<br>**Cavuliceddu** (TP-Erice, Riserva dello Zingaro)<br>**Mazzarreddi, Spicuna sarbaggia** (AG-Sant'Angelo Muxaro, San Biagio Platani)<br>**Qualeddu** (TP-Erice)<br>**Spicuni, Assani** (AG-Burgio, Lucca Sicula, Ravanusa)
Brassica tournefortii Gouan<br>(*Brassicaceae*)	**Musuluchi** (TP)
Bunias erucago L.<br>(*Brassicaceae*)	**Catanzículi** (CT-Regaina)<br>**Cicoina** (CT-Maletto, Bronte)<br>**Cicoina sarbaggia** (CT-Adrano)<br>**Ciconia** (CT-Nicolosi, Pedara)<br>**Ciconia di vigna** (CT-Milo, Giarre, Venerina),<br>**Erba stidda** (CT-Maletto).<br>**Cicoira, Mazzarelli** (CT-Randazzo)<br>**Spinacia** (CT-Zafferana, Linguaglossa,)<br>**Spinacia sarvaggia** (CT-Castiglione)<br>**Triuliddi** (CT-Belpasso)
Calamintha nepeta (L.) Savi<br>(*Lamiaceae*)	**Amintastru** (AG-Sant'Angelo Muxaro, San Biagio Platani)<br>**Menta** (AG-Licata); (CL-Butera)<br>**Nieputa, Nieputedda** (Me-Isole Eolie)<br>**Nipitedda** (AG-Bisaquino, Aragona, Burgio, Cammarata, Casteltermini, Cianciana, Lucca Sicula, Ravanusa); (PA-Geraci Siculo, Giardinello, Grisi)<br>**Niputedda** (RG-Vittoria); (SR-Pachino); (TP-Riserva dello Zingaro)
Capparis ovata Desf.<br>(*Capparidaceae*)	**Cappero** (CL-Caltanisetta, Delia)<br>**Cappiru** (RG-Vittoria)<br>**Chiapparu sarbaggiu** (AG-Raffadali)<br>**Chiappara** (AG-Favara, Raffadali)<br>**Chiappara sarbaggiu** (Raffadali)<br>**Chiappara sarvaggia, Chiapparedda liscia** (CL-Butera, Riesi)<br>**Chiappareddra** (CL-Campofranco, Sutera)<br>**Chiappari** (AG-Aragona, Cattolica Eraclea , Realmonte)<br>**Chiappira** (ME-Tusa)<br>**Ciapparedda** (AG-Licata, Campofranco); (CL-Sutera)<br>**Riappari** (AG-Montevago)
Capparis spinosa L.<br>(*Capparidaceae*)	**Cappero** (TP-Riserva dello Zingaro); (ME-Isole Eolie)<br>**Chiappaira** (PA-Caccamo)<br>**Chiappara** (ME-Galati Mamertino); (AG- Sambuca Di Sicilia); (TP-Pantelleria); (CT- Giarre, S. Giovanni la Punta); (CL-Caltanissetta); (PA- Partinico, Vicari, Villafrati);

	(TP-Castellammare del Golfo, Castelvetrano, Custonaci, Favignana, Levanzo, Marettimo, Mazara del Vallo, Partanna, Poggio Reale, Salaparuta, San Vito lo Capo, Valderice, Riserva dello Zingaro)<br>**Chiappare** (CT-Santa Venerina, Zafferana)<br>**Chiappari** (TP-Favignana, Levanzo, Marettimo)<br>**Chiapparina** (AG-Realmonte); (CT-Nicolosi)<br>**Chiapparo** (PA-Ustica)<br>**Chiapparu** (CT-Adrano, Biancavilla, Bronte, Santa Venerina, Zafferana)<br>**Chiapparu manzu** (AG-Raffadali)<br>**Chiappiru** (CT-Castiglione)<br>**Chiappuli** (CT-Linguaglossa)<br>**Ciàppiru** (TP-Riserva dello Zingaro)<br>**Riappari** (SR-Pachino)
Carduncellus pinnatus (Desf.) DC.<br>(*Asteraceae*)	**Carduncellu** PA (Petralia Soprana , Petralia sottana)
Cardus argyroa Biv.<br>(*Asteraceae*)	**Napordi d'acqua** (AG-Sant'Angelo Muxaro)
Cardus pycnocephalus L.<br>(*Asteraceae*)	**Scoddi** (EN-Piazza Armerina)
Carlina sicula Ten.<br>(*Asteraceae*)	
Carthamus lanatus L.<br>(*Asteraceae*)	**Vavanazzi** (AG-Sambuca Di Sicilia)
Castanea sativa Miller<br>(*Fagaceae*)	**Castagnera** (ME-Galati Mamertino, Tortorici)<br>**Castagna** (PA)
Celtis australis L.<br>(*Ulmaceae*)	**Caccamo** (PA-Castronovo)<br>**Milicuccu** (CT-Bronte)<br>**Milikukku** (RG-Vittoria)
Centaurea calcitrapa L.<br>(*Asteraceae*)	**Aprocchiu** (AG-Aragona, Sant'Angelo Muxaro)<br>**Abbrocciu** (AG-Licata)<br>**Grap l'occhi** (EN-Piazza Armerina)<br>**Occhi n'Cristu** (AG-Montallegro); (CT-Catania, Acireale)
Centaurea nicaeensis All.<br>(*Asteraceae*)	**Approchiu** (AG-Aragona)
Centaurea solstitialis L. subsp.*schouwii* (DC.) Dostal<br>(*Asteraceae*)	**Gattaredda** (PA-Gangi, San Mauro Castelverde)
Centranthus ruber L.<br>(*Valerianaceae*)	**Pasqua rossa, Pasqua bianca** (SR-Noto)<br>**Valeriana** (CL-Caltanissetta, San Cataldo, Serradifalco); (AG-Cattolica Eraclea, Sambuca Di Sicilia, San Biagio Platani)
Ceratonia siliqua L.<br>(*Fabaceae*)	**Carruba** (PA-Borghetto), (TP- Custonaci, Erice, Mazara del Vallo, Pantelleria, Trapani, Valderice)<br>**Carrubbu** (PA-Bisaquino); (TP-Gibellina)<br>**Cicibanoz** (PA-Contessa Entellina)
Chamaerops humilis L.<br>(*Palmae)*	**Giummarra** (CL-Mazzarino, Riesi)<br>**Safagguni** (RG-Vittoria)<br>**Scupazzu** (RG-Vittoria)
Chenopodium album L.subsp. *album*<br>(*Chenopodiaceae*)	**Erva fitenti** (RG-Vittoria)
Chondrilla juncea L.<br>(*Asteraceae*)	**Cud'e attu** (CT-Linguaglossa)<br>**Cudidda** (CT-Nicolosi, Zafferana, Ragaina, Belpasso)<br>**Curi î suggi** (CT-Bronte)<br>**Curidda** (CT-Pedara)<br>**Cutuledda** (CT-Adrano)<br>**Cutulidda** (CT-S.Venerina, Zafferana, Milo, Linguaglossa)<br>**Inestruola** (CT-Randazzo)<br>**Inestruora** (CT-Randazzo)<br>**Inistrora** (CT-Maletto
Chrysanthemum coronarium L.	**Ciuri di cacamaiu** (AG-Sant'Angelo Muxaro)

(*Asteraceae*)	**Maiazzu** (CL-Butera, Riesi)<br>**Maiu** (AG-Licata)
Cichorium intybus L.<br>(*Asteraceae*)	**Cicoira** (ME-S.Stefano di Camastra)<br>**Cicoria** (AG-Burgio, Lucca Sicula, Ravanusa, Cattolica Eraclea, Comitini, Favara, Realmonte, Siculiana, Campobello di Licata, Canicattì, Raffadali, Sant'Angelo Muxaro, San BiagioPlatani, Montallegro),<br>(PA-Bisaquino); (ME-Mistretta); (TP-Trapani, Erice, Custonaci); (CT-Catania, Acireale, Nicolosi)<br>**Cicoria amara** (TP-Pantelleria)<br>**Cicoria catalogna** (AG-Aragona, Realmonte, Montevago)<br>**Cicoria di campagna** TP-Riserva dello Zingaro)<br>**Cicoria di chianca** (CL-Butera, Riesi)
Clematis vitalba L.<br>(*Ranunculaceae)*	**Liara** (ME-Tortorici), (CT-Linguaglossa, Ragaina)<br>**Liareddi** (ME-Mistretta)<br>**Ligara** (CT-Randazzo, Maletto, Bronte)<br>**Mitabbi** (CT-Castiglione)<br>**Mitarbi** (CT-Milo)<br>**Vitalba** (PA-Cefalù)<br>**Viterbi** (CT-Linguaglossa)
Corylus avellana L.<br>(*Corylaceae*)	**Nucidda** (PA-Bisaquino)
Crataegus azarolus L.<br>(*Rosaceae*)	**Azaloru** (TP-Trapani),<br>**Lazzarolu, Azzaloru, Lanzarolu giarnu** (TP-Riserva dello Zingaro)
Crataegus monogyna Jacq.subsp.*monogyna*<br>(*Rosaceae*)	**Br'zulinu** (EN-Piazza Armerina)<br>**Brizzulina** (AG-Sambuca di Sicilia)<br>**Brizzulino** (ME-Tortorici)<br>**Bruzzellinu** (ME-Mistretta)<br>**Russulina** (SR-Noto)<br>**Ursuliddru** (AG-Campobello di Licata, Canicattì, Ravanusa Montallegro); (CT-Catania, Acireale)<br>**Vrizzulina, Zinzuli** (AG-Favara)
Crepis bursifolia L.<br>(*Asteraceae*)	**Ricuttella** (CT-Randazzo)<br>**Rizzaredda** (CT-Ragaina)<br>**Rizzarella** (CT-Maletto)
Crepis vesicaria L. subsp.*vesicaria*<br>(*Asteraceae*)	**Cicoria amara** (TP-Buseto Palizzolo, Favignana, Levanzo, Marettimo)<br>**Cicoria missinisa** (AG-Aragona)<br>**Cicuriuni** (EN-Piazza Armerina)<br>**Erba d'acietri** (ME-Tusa)<br>**Occhi pirnici** (ME-Santo Stefano di Camastra)
Crithmum maritmum L.<br>(*Apiaceae*)	**Finucchieddu di mari, Finucchieddu marinu** (TP-Favignana, Levanzo, Marettimo)
Cydonia oblonga L.<br>(*Rosaceae*)	**Cotogno** (PA-Ustica)<br>**Cutugnu** (PA-Bisaquino); (ME-Caronia); (TP Erice, Gibellina, Salemi, Trapani, Vita), (AG-Sambuca di Sicilia)
Cynara cardunculus L. subsp. *cardunculus*<br>(*Asteraceae*)	**Cacocciula sarbaggia** (AG-Raffadali)<br>**Cacocciuliddu sarbaggiu** (AG-Siculiana)<br>**Cacocciuliddu spinusu** (AG-Raffadali)<br>**Cacocciulu sarbaggiu** (AG-Sant'Angelo Muxaro)<br>**Cacucciuliddu** (AG-Licata)<br>**Carcocciula sarvaggia** (PA-Bisaquino); (AG-Sambuca di Sicilia)<br>**Carduna servaggi** (ME-Mistretta)<br>**Carduni** (AG-Campobello di Licata, Canicattì, Ravanusa, Montallegro); (CT-Acireale, Catania)<br>**Carduni centutesti** (AG-Aragona, Realmonte)<br>**Carduni amaru, Caccucciuledda, Carduni sarvaggiu** (AG-Bivona)
Diplotaxis crassifolia DC.<br>(*Brassicaceae*)	**Erva cavulara, cavuliceddi** (AG-Sant'Angelo Muxaro, San Biagio Platani)
Diplotaxis erucoides (L.)DC.<br>(*Brassicaceae*)	**Ciuri bianchi** (PA-Ustica)<br>**Finacciolu** (AG-Licata)<br>**Finacciuola** (AG-Montevago)

	**Finacciuolu** (CL-Mussomeli)<br>**Lariani** (AG-Favara, Raffadali)<br>**Lassanu d'aglia** (PA-Prizzi)<br>**Pissineddi** (AG-Cianciana)<br>**Razzi** (AG-Sambuca Di Sicilia)<br>**Razzina, Ruca, Sinicciola** (AG-Aragona, Cattolica Eraclea, Raffadali)<br>**Ruca** (AG-Aragona)<br>**Sanacciola** (RG-Vittoria)<br>**Sanaccioli** (EN-Piazza Armerina)<br>**Sanacciolo** (CL-Butera, Riesi)<br>**Senàpa** (CL-Caltanissetta , San Cataldo, Serradifalco)<br>**Sinacciola** (RG-Vittoria), (AG-Cattolica Eraclea, Realmonte, Sant'Angelo Muxaro, San Biagio Platani, Bivona); (CT-Nicolosi)<br>**Sinacciuddu** (AG-Cammarata, Casteltermini)<br>**Sinacciulu** (Cl-Butera, Riesi, Mazzarino, Sommatino)
Diplotaxis tenuifolia (L.) DC.<br>(*Brassicaceae*)	**Ruca** (CT-Giarre), (TP-Trapani, Valderice, Erice)
Erucastrum virgatum (Presl) Presl<br>(*Brassicaceae*)	**Sinapi** (PA-Ganci)
Eryngium campestre L.<br>(*Apiaceae*)	**Insalata ru riavuliu, panicallu** (CL-Butera, Caltanissetta, San Cataldo, Serradifalco)<br>**N'zalata du diavulu, N'zalata du scessu** (TP-Riserva dello Zingaro)<br>**Panicaudu** (CL-Caltanisetta, San Cataldo, Santa Caterina Villermosa, Serradifalco)<br>**Panicauru** (TP-Riserva dello Zingaro)
Fedia cornucopiae (L.) Gaertner<br>(*Valerianaceae*)	**Lattucheddra ri maio** (ME-Mistretta)<br>**Lattuchedda modda** (AG-Lampedusa)<br>**Spazzaquartara** (ME-Tortorici)<br>**Maggio** (ME-Galati Mamertino)<br>**Peri ri ciocca** (TP-Favignana)<br>**Peri ciocca, Ervi moddi** (TP-Riserva dello Zingaro)<br>**Spezzaquartari** (ME-Tusa)
Ferula communis L.<br>(*Apiaceae*)	**Fella, Ferla** (TP-Riserva dello Zingaro)<br>**Ferra** (RG-Vittoria), (CT-Bronte)
Ficus carica L.<br>(*Moraceae*)	**Ficara** (ME-Tortorici)<br>**Fichera** (ME-San Fratello, Santo Stefano di Camastra)<br>**Ficu** (TP-Trapani), (AG-Campobello di Licata, Canicattì, Ravanusa, Realmonte, Montallegro); (CT-Acireale, Catania, Nicolosi), (Me-Isole Eolie)<br>**Ticchiara, Ficu** (AG-San Biagio Platani, Sant'Angelo Muxaro); (ME-Galati Mamertino, Longi, Mirto, Mistretta, Naso)
Foeniculum vulgare Miller.subsp.*piperitum* (Ucria) Coutinho<br>(*Apiaceae*)	**Finucchieddru silvaticu** (TP)
Foeniculum vulgare Miller subsp. *vulgare*<br>(*Apiaceae*)	**Finichieddu sarbaggiu** (AG-Comitini, Favara, Raffadali, Siculiana)<br>**Finocchi** (ME-Mistretta)<br>**Finocchiu** (AG-Realmonte, Sambuca di Sicilia); (CT-Nicolosi); (ME-Galati Mamertino, Longi, Tortorici, Isole Eolie)<br>**Finocchiu di campagna** (AG-Burgio, Lucca Sicula, Ravanusa, Montevago)<br>**Finocchiu duci** (AG-Raffadali)<br>**Finocchiu 'ngranatu** (AG-Canicattì)<br>**Finocchiu sarbaggiu** (AG-Raffadali, Sant'Angelo Muxaro, San Biagio Platani, Bivona, Casteltermini Cianciana)<br>**Finocchiu sarvaggiu** (TP-Pantelleria, Trapani, Erice, Custonaci)<br>**Finocciu** (ME-San Fratello)<br>**Finucchieddu** (AG-Isole Pelagie)<br>**Finucchieddu di giru** (AG-Aragona, Raffadali, Realmonte)<br>**Finucchieddu sarbaggiu, Finucchieddru rizzu** (AG-Raffadali)<br>**Finucchieddu rizzu, Finocchieddu î timpa** (CT-Adrano, Belpasso,

	Biancavilla Bronte, Castiglione, Linguaglossa, Maletto, Milo, Nicolosi, Pedara, Ragaina, Randazzo, S.Giovanni la Punta, S.Venerina, Zafferana)<br>**Finucchieddu sarvaggiu** (CT-Giarre)<br>**Finuccieddu sarvaggiu** (AG-Licata)<br>**Finuccieddu rizzu** (AG-Licata)
Fraxinus ornus L.<br>(*Oleaceae*)	**Manna** (PA-Castelbuono)
Glycyrrhyza glabra L.<br>(*Fabaceae*)	**Niculizia** (AG-Aragona, Cattolica Eraclea, Licata, Sambuca di Sicilia, Sant'Angelo Muxaro, San Biagio Platani)<br>**Nigulizia** (AG-Casteltermini, Cianciana)<br>**Niquirizia** (AG-Montevago)
Hedysarum coronarium L.<br>(*Fabaceae*)	**Sudda** (AG-Aragona, Sant'Angelo Muxaro, San Biagio Platani, Calatafimi, Campobello di Mazara, Salemi, Vita, Custonaci)<br>**Suddra** (CL-Campofranco, Sutera, Comitini, Bivona)
Helicrysum italicum (Roth) Don<br>(*Asteraceae*)	**Rosamarina sarvaggia mascolina** (AG-Sant'Angelo Muxaro
Hermodactylis tuberosus (L.) Salish.<br>(*Iridaceae*)	**Buttuni di iaddu**, (CT-Linguaglossa, Castiglione)<br>**Cantaliaddi, Cantajaddu, Canta addu** (CT-Ragaina)<br>**Castagnotto** (CT-Randazzo)<br>**Cricch'êaddu** (CT-Milo)<br>**Pizzicaladdi** (CT-Zafferana)<br>**Sucameli** (CT-Linguaglossa)
Hirschfeldia incana (L.) Lagr.F.<br>(*Brassicaceae*)	**Assini biancu** (PA-Ganci)<br>**Sanapieddu duci** (CL-Butera, Riesi)
Hyoseris radiata L.<br>(*Asteraceae*)	**Attalebbra** (TP-Riserva dello Zingaro)<br>**Buttuni ri gallo** (CT-Randazzo)<br>**Cardeddi di serpi** (TP-Favignana, Levanzo, Marettimo)<br>**Cicuriuni** (AG-Casteltermini)<br>**Cudduredda** (CT-Ragaina)<br>**Cudduruni (**CT-Ragaina)<br>**Erba duci** (CT-Linguaglossa)<br>**Perigallu** (CT-Maletto)<br>**Pidicudda** (PA-Bisaquino)<br>**Tarassaco** (TP-Campobello di Mazara)
Hypochoeris cretensis (L.) Chaub.et Bory<br>(*Asteraceae*)	**Citula duci** (PA-Vicari)
Hypochoeris laevigata L.<br>(*Asteraceae*)	**Razza** (ME-Tortorici)
Hypochoeris radicata L.<br>(*Asteraceae*)	**Coscî vecchia** (CT-Nicolosi, S. Giovanni la Punta, Pedara, S.Venerina, Zafferana, Milo, Linguaglossa, Adrano, Biancavilla, Ragaina)<br>**Coscia vecchia** (CT-Belpasso)<br>**Costi vecchia** (CT-Castiglione, Randazzo, Maletto, Bronte)<br>**Costa ri vecchia** (CT-Randazzo)<br>**Costavecchia** (CT-Bronte)<br>**Cucummareddu** (CT-Ragaina Nicolosi)<br>**Erva rassudda** (PA-Partitico)<br>**Micci scalora** (CT-Linguaglossa)<br>**Scaranzinzuli, Cazzicatummuli** (CT-Zafferana)<br>**Scarla** (PA-Gangi, San Mauro Castelverde)<br>**Scarri** (ME-Mistretta)
Hypochoeris radicata L. subsp.*neapolitana* (DC.) Guad.<br>(*Asteraceae*)	**Cosci vecchia** (CT-Nicolosi, S. Giovanni la Punta, Pedara, S.Venerina, Zafferana, Milo, Linguaglossa, Adrano, Biancavilla, Ragaina)<br>**Cosciavecchia** (CT-Belpasso)<br>**Costi vecchia** (CT-Castiglione, Maletto, Bronte; ME-Tortorici)<br>**Costa ri vecchia** (CT-Bronte)<br>**Costavecchia** (CT-Bronte)<br>**Scarri** (ME-Mistretta)

Juncus acutus L. (*Juncaceae*)	**Iuncu** (AG)
Lactuca serriola L. (*Asteraceae*)	**Lattuca sarbaggia** (CT-Bronte)
Lactuca viminea (L.) Presl. (*Asteraceae*)	**Cacciacunigghia** (CT-Milo)<br>**Caccialepri, pirnici amara** (CT-Linguaglossa)<br>**Caranzicula** (CT-Zafferana)<br>**Cardedda di petra** (CT-Castiglione)<br>**Ervascursuni** (CT-S. Giovanni la Punta)<br>**Evva di scursuni** (CT-Nicolosi)<br>**Evva di scussuni** (CT-Belpasso)<br>**Evvascursuni** (CT-Nicolosi)<br>**Gattaru** (CT-Bronte)<br>**Lattughedda du signori** (CT-Adrano<br>**Ntossicaceddi** (CT-Biancavilla, Ragaina)<br>**Pieririnigghiu** (CT-Randazzo)<br>**Perinigghiu** (CT-Randazzo, Bronte)<br>**Pisciacunigghia** (CT- Milo, S. Venerina,)<br>**Scursunara** (CT-Pedara, Ragaina)<br>**Virinella** (CT-Maletto)
Lamium flexuosum Ten. (*Lamiaceae*)	**N'zinzili** (ME-Galati Mamertino, San Marco d' Alunzio)
Lathyrus articulatus L. (*Fabaceae*)	**Fajoru** (CT-Randazzo)<br>**Fasola** (CT-Adrano)<br>**Fasoli** (CT-Ragaina)<br>**Fasuledda** (CT-Belpasso)<br>**Fasuledda sarbaggia** (CT-Nicolosi)<br>**Pusedda sabbaggia** (CT-Castiglione)<br>**Puseddu sarbaggiu** (CT-Bronte)<br>**Vizza** (CT-Santa Venerina, Zafferana , Maletto)
Lathyrus clymenum L. (*Fabaceae*)	**Piseddu sarbaggiu** (AG-Sambuca Di Sicilia)
Lathyrus ochrus (L.)DC. (Fabaceae)	**Piseddu sarbaggiu** (AG-Raffadali),<br>**Fasolu sarbaggiu** (AG-Sant'Angelo Muxaro)
Lathyrus odoratus L. (Fabaceae)	**Fasolu sarbaggiu** (AG-Sant'Angelo Muxaro)<br>**Piseddu sarbaggiu** (AG-Aragona, Raffadali)<br>**Pisidduzzu sarvaggiu** (PA-Partinico)
Lathyrus sylvestris L. (Fabaceae)	**Cessavuoi** (ME-Santo Stefano di Camastra)<br>**Gelsaù** (ME-Mistretta)
Laurus nobilis L. (*Lauraceae*)	**Addagaru** (ME-Raffadali, Sant'Angelo Muxaro, San Biagio Platani, Siculiana, Montevago)<br>**Addaura** (PA-Palermo)<br>**Addauro** (CL-Caltanissetta, Delia)<br>**Addauru** (AG-Aragona, Realmonte); (ME- Mistretta, San Marco di Alunzio, Isole Eolie); (RG-Vittoria); (PA- Ustica); (TP-Buseto Palizzolo, Calatafimi, Castellammare del Golfo, Custonaci, Erice, Favignana, Levanzo, Marettimo, Mazara del Vallo, San Vito lo Capo, Valderice, Riserva dello Zingaro)<br>**Addavuru** (PA-Bisaquino); (TP-Gibellina, Salemi, Vita); (AG-Sambuca di Sicilia)<br>**Addrauru** (AG-Campobello di Licata, Canicattì, Ravanusa, Realmonte, Montallegro); (TP-Mazara del Vallo); (CT- Catania, Acireale, Nicolosi)<br>**Dadàr** (ME-San Fratello)<br>**Dàuru** (EN-Piazza Armerina); (ME-Galati Mamertino, Tortorici, Naso)
Lavatera trimestris L. (*Malvaceae*)	**Marba, Bianca russina** (AG-Comitini, Favara, San Biagio Platani)
Leontodon tuberosus L. (*Asteraceae*)	**Occhiu di pinnici** (CT-Castiglione)<br>**Occhi pinnici** (CT-Randazzo)<br>**Lattughedda** (CT-Ragaina)

Leopoldia comosa (L.) Parl. (*Liliaceae*)	**Agghioru niuru** (CT-Randazzo)<br>**Cipuddazza** (CT-Biancavilla, Regaina)<br>**Cipuddazzu** (CT-Adrano, Maletto Ragaina)<br>**Cipuddruzza, Trubittuni** (CL-Caltanissetta, San Cataldo, Serradifalco)<br>**Cipudduzza sarvaggia** (ME-Salina)<br>**Cipudduzzu** (CT-Nicolosi, Milo, Adrano)<br>**Purrazzu** (CT-Zafferana)
Lycium europaeum L. (*Solanaceae)*	**Spina santa** (AG-Aragona, Comitini, Licata, Raffadali, Realmonte, Siciliana);<br>(EN-Piazza Armerina)<br>**Spinasanta** (PA-Ganci)
Lupinus albus L. (Fabaceae)	**Luppini** (ME-Mistretta, Isole Eolie)
Malva nicaensis All. (*Malvaceae*)	**Marba** (AG-Lucca Sicula)<br>**marva** (EN-Piazza Armerina)
Malva sylvestris L. (*Malvaceae*)	**Marba** (CT-Zafferana, Milo, Linguagloss, Ragaina)<br>**Marva** (CT-Nicolosi, S. Giovanni la Punta, Castiglione, Randazzo, Maletto, Biancavilla, Regaina)<br>**Mavva** (CT-Nicolosi, Pedara, Zafferana, Adrano, Belpasso)<br>**Mavvascu** (CT-Bronte)
Mentha aquatica L. (*Lamiaceae*)	**Amenta** (PA-Ganci)
Mentha pulegium L. (*Lamiaceae*)	**Amenta** (Me-Alicudi, Filicudi, Lipari)<br>**Menta** (ME- Panarea, Salina, Stromboli, Vulcano)
*Mentha spicata*L. subsp.*glabrata* (Lej et Court.) Lebeau (*Lamiaceae*)	**Menta** (CT-Acireale)
Mentha spicata L. subsp. *spicata* (*Lamiaceae*)	**Amentastru, Scordiu, Rintuzza, Menta** (CT-Acireale); (PA-Bisaquino); (TP-Castellammare del Golfo, Mazara Del Vallo, Partanna Poggioreale)
Mentha suaveolens Ehrh. (*Lamiaceae*)	**Amenta** (AG-Campofranco)<br>**Amenta sarvaggia** (AG-Casteltermini)<br>**Amintastru** (AG-Cattolica Eraclea, Casteltermini)<br>**Mintastru** (AG-Sambuca di Sicilia, Montallegro); (CT-Catania Acireale)
Mespilus germanica L. (*Rosaceae*)	**Nespula d'invernu**
Morus alba L. (*Moraceae*)	**Ceusa bianca** (TP-Pantelleria)<br>**Ceusi** (PA-Ustica)<br>**Ceuso bianco** (SR-Noto)<br>**Ceusu biancu** (TP-Erice, Mazara del Vallo)
Morus nigra L. (*Moraceae*)	**Ceusa nivura** (TP-Pantelleria)<br>**Ceuso nero** (SR-Noto)<br>**Ceuso nivuru** (TP-Mazara del vallo)<br>**Ceusu niuru** (TP-Erice, Custonaci)<br>**Cevusi** (PA-Ustica)
Moricandia arvensis (L.)DC. (*Brassicaceae*)	**Cavulu sarvaggiu** (AG-Sant'Angelo Muxaro),
Narcissus tazetta L. subsp. *tazetta* (*Amaryllidaceae*)	**Agghi porri** (TP-Calatafimi)
Myrtus communis L. (*Myrtaceae*)	**Mirtu, Murtidda** (PA-Borgetto, Caccamo, Camporeale, Polizzi Generosa, San Giuseppe Jato); (SR-Pachino)<br>**Murtitra** (ME-Caronia, Messina, Tusa)
*Nasturtium officinale (*R.)Br. (*Brassicaceae*)	**Crisciuna** (ME-Mistretta)<br>**Crisciuni** (ME-Butera, Mazzarino, Riesi, Sommatino, Bronte, Corleone, San Giuseppe Jato)<br>**Scavuni** (AG-Sambuca di Sicilia)<br>**U' crisciuni** (PA-Castelbuono)
Notobasis syriaca (L.) Cass. (*Asteraceae*)	**Piscialasinu** (AG-Sant'Angelo Muxaro, Campofranco); (PA-Prizzi)<br>**Lamanna** (PA-Collesano)

Onopordum horridum Viv.<br>(*Asteraceae*)	**Napordu** (TP-Riserva dello Zingaro)
Onopordum illyricum L.<br>*(*Asteraceae*)*	**Caccocciulidda sarvaggia** (PA-Montelepre)<br>**Cardu anapordu** (PA-Collesano)<br>**Muìni** (CT-Castiglione)<br>**Munaceddi** (AG-Bivona, Cammarata)<br>**Munaceddu** (CT-Randazzo)<br>**Munacheddu** (PA-Castonovo)<br>**Muni** (CT-Linguaglossa)<br>**Napordu** (AG-Sant'Angelo Muxaro, San Biagio Platani)<br>**Napuordo** (SR-Avola, Noto)<br>**Napuordu** (CL-Campofranco, Riesi, Sutera)<br>**Napurda** (PA-Petralia Soprana, Petralia Sottana, Polizzi generosa, Pollina, San Mauro Castelverde)<br>**Napurdu** (PA-Gangi)<br>**Piddonicu** (CT-Maletto)<br>**Scaddallasinu** (CT-Milo)<br>**Trimazzi** (CT-Nicolosi)<br>**Trummazzi** (CT-Ragaina)<br>**Zanuri** (EN-Piazza Armerina)
Opuntia ficus indica (L.)Miller<br>(*Cactaceae*)	**Ficalinni** (ME-Filicudi, Lipari, Panarea, Salina, Stomboli, Vulcano)<br>**Fico d'India** (CL-Caltanissetta, Delia)<br>**Ficodindia** (PA-Polizzi Generosa)<br>**Ficubala** (TP-Calatafimi)<br>**Ficudinia** (PA-Bisaquino); (AG-Campobello di Licata, Burgio, Lucca Sicula, Montallegro); (CT-Catania, Acireale); (PA-Ganci, Castellammare Del Golfo, Mazara Del Vallo, Partanna, Poggioreale)<br>**Ficudinna** (CL-Butera Riesi)<br>**Ficudinni** (AG-Montevago)<br>**Ficudinnia** (AG-Aragona, Realmonte)<br>**Ficulinia** (AG-Sambuca Di Sicilia)<br>**Ficumori** (TP-Riserva dello Zingaro)<br>**Ficurigna** (Caccamo, (TP-Favignana, Levanzo, Marettimo, Riserva dello Zingaro)<br>**Ficurini** (TP-Buseto Palizzolo)<br>**Ficurinia** (TP-Partitico, San Cipirello, Gibelina, Salemi, Vita)<br>**Ficurinii** (TP-Favignana, Levanzo, Marettimo)<br>**Ficurinnia** (TP-Campobello di Mazara, Mazara del Vallo, Riserva dello Zingaro)<br>**Ficurinnia, ficadinniera** (ME-Galati Mamertino, Longi, S.Agata di Militello, Santo Stefano di Camastra, Tortorici)<br>**Fikupali** (RG-Vittoria); (TP-Riserva dello Zingaro)
Origanum heracleoticum L.<br>(*Lamiaceae*)	**Arìanu** (AG-Aragona, Realmonte, Montevago); (CT-Giarre); (CL-Mussomeli); (TP- Erice Castelvetrano, Favignana, Marittimo, Mazara del Vallo, Partanna, Poggio Reale, Salaparuta, Riserva dello Zingaro)<br>**Arìfinu** (RG-Vittoria)<br>**Ariganu** (PA-Chiusa Sclafani, Petralia Soprana, Petralia Sottana); (AG- Bugio, Licata, Raffadali, Sant'Angelo Muxaro, San Biagio Platani, Ravanusa)<br>**Arrìano** (PA-Palazzo Adriano , Castronovo); (TP- Favignana)<br>**Arrianu** (PA-Borghetto, Corleone, Roccamena, S. Giuseppe Jato)<br>**Origano** (CL-Caltanissetta, Delia)<br>**Origanu** (CL-Campofranco, Sutera)<br>**Renìu** (PA-Caccamo)<br>**Rianu** (AG-Aragona, Favara, Realmonte, Menfi)<br>**Rianu** (PA-Galati Mamertino, Longi, Castelbuono)<br>**Rienu** (ME-Mistretta)<br>**Rigane** (PA-Piana degli Albanesi)<br>**Riganu** (CL-Caltanissetta, San Cataldo, Santa Caterina Villermosa, Serradifalco); (ME- Tortorici, S. Stefano di Camastra)<br>**Riganu sarvagiu** (EN-Piazza Armerina)<br>**Riùni** (PA-Collesano)

Origanum majorana L. (*Lamiaceae*)	**Maggiorana** (AG-Campobello di Licata, Canicattì, Ravanusa)<br>**Maiurana** (PA-Palazzo Adriano, Chiusa Sclafani), (TP-Campobello di Mazara)<br>**Majurana** (AG-Montallegro); (CT-Catania, Acireale)
Origanum vulgare L. (*Lamiaceae*)	**Rianu** (PA-Ustica)<br>**Ricunu** (SR-Pachino)
Oryzopsis miliacea (L.) Ash.et Schweinf (*Poaceae*)	**Fiurbugliuni** (AG-Licata)
Oxalis pes-caprae L. (*Oxalidaceae*)	**Agari duci** (PA-Castronovo)<br>**Agra e duci, Caracitula** (AG-Raffadali)<br>**Agri e duci** (AG-Sambuca Di Sicilia)<br>**Airu e duci** (CT-Giarre); (TP-Castelvetrano, Partanna, Poggio Reale, Salaparuta)<br>**Auru acitu** (AG-Licata)<br>**Cacitula** (AG-Sant'Angelo Muxaro)<br>**Caracitula** (AG-Montevago)<br>**Cicireddu** (TP-Riserva dello Zingaro)<br>**Gracìtula** (CL-Butera, Maazarino, Riesi, Sommatino)<br>**Jathe iembel** (PA-Contessa Entellina, Collesano)
Papaver rhoeas L. subsp. *rhoeas* (*Papaveraceae*)	**Paparina russa, Paparinazzu, Paparina** (AG-Campobello di Licata, Canicattì, Ravanusa, Montallegro); (CT-Acireale); (CL-Butera, Riesi)
Papaver setigerum DC. (*Papaveraceae*)	**Paparina manza** (TP-Partitico); (PA-Petralia Soprana)
Petroselinum sativum Hoffm. (*Apiaceae*)	**Piddrusino, Piddusino** (TP-Erice, Valderice, Mazara del Vallo)<br>**Pitrusino** (TP-Erice, Gibellina, Salemi, Vita)<br>**Pitrusinu** (PA-Bisaquino); (AG-Campobello di Licata, Canicattì, Ravanusa)
Phagnalon saxatile (L.) Cass. (*Asteraceae*)	**Rosamarina sarvaggia femminina** (AG-Sant'Angelo Muxaro)
Phlomis fruticosa L. (*Lamiaceae*)	**Salvia** (CL-Butera, Mazzarino, Riesi.Sommatino)<br>**Sarbia** (CL-Butera , Riesi)<br>**Sarvia sarvaggia** (AG-Cianciana)<br>**Sucameli** (AG-Raffadali)
Picris echioides L. (*Asteraceae*)	**Asparedda** (AG-Aragona, Sant'Angelo Muxaro)<br>**spinedda** (TP-Vita); (PA-San Cipiriello, Roccamena)<br>**Spiredda** (PA-Castelbuono)
Pinus pinea L. (*Pinaceae*)	**Pinu** (AG-Canicattì, Ravanusa)
Pistacia vera L. (*Anacardiaceae*)	**Festuca** (AG-Canicattì, Campobello di Licata, Ravanusa)<br>**Pastuca** (SR-Noto, Avola); (CT-Brolo
Pisum sativum L. subsp. *sativum* (*Fabaceae*)	**Casola, Piseddri** (AG-Sambuca Di Sicilia)
Pyrus amygdaliformis Vill. (*Rosaceae*)	**Prainu** (ME-Mistretta)
Plantago lagopus L. (*Plantaginaceae*)	**Cutidduzzi** (ME-Mistretta)
Plantago serraria L (*Plantaginaceae*)	**Cutidduzzu** (ME-Santo Stefano di Camastra)
Portulaca oleracea L. (*Portulacaceae*)	**Burdulaca** (TP-Pantelleria)<br>**Cucciara** (SR-Pachino)<br>**Pirciddana** (PA-Pollina)<br>**Porcellana** (AG-Linosa)<br>**Pucciddana** (CT-Nicolosi, Zafferana, Linguaglossa, Ragaina, Belpasso); (ME-Isole Eolie)<br>**Puccillana** (CT-Maletto, Bronte, Biancavilla)<br>**Purciddana** (PA-Bisaquino); (ME-Galati Mamertino, San Fratello, San Marco d' Alunzio, Tortorici); (CT-S. Giovanni la Punta, Pedara, S.Venerina, Zafferana, Milo, Linguaglossa, Castiglione, Adrano,)<br>**Purciddana** (AG-Cattolica Eraclea, Sambuca Di Sicilia), (PA-San Biagio Platani Castellana Sicula, Corleone, Ganci, Isnello, Pollina,

	San Mauro Castelverde, Ustica), (RG-Vittoria); (TPCastelvetrano, Favignana, Levanzo, Partanna, Poggio reale, Salaparuta);<br>(EN-Piazza Armerina)<br>**Purciddrana** (RG-Vittoria), (AG-Ravanusa , Campofranco)<br>**Purciddrara campofranco**<br>**Purcillana** (CT-Randazzo)
Prasium majus L.<br>(*Lamiaceae*)	**Camedriu biancu** (TP-Riserva dello Zingaro)<br>**Erva thè** (TP-Favignana),<br>**Menta sarvaggia** (PA-Chiusa Sclafani)<br>**Tè nustrali** (TP-Riserva dello Zingaro)
Prunus dulcis (Miller) D.A Webb.<br>(*Rosaceae*)	**Miennulu** (TP-Riserva dello Zingaro)<br>**Miennula** (TP-Trapani, Erice, Valderice, Custonaci, Riserva dello Zingaro)
Prunus spinosa L.<br>(*Rosaceae*)	**Atrignu** (PA-Prizzi)<br>**Brugnole, trigne** (ME-Mistretta)<br>**Pruna** (PA-Chiusa Sclafani)<br>**Prunu sarvaggiu** (PA-Camporeale, Villafrati)<br>**Vrignola** (PA-Ganci, Polizzi Generosa)
Punica granatum L.<br>(*Punicaceae*)	**Granatu** (AG-Sambuca Di Sicilia); (PA-Borgetto, Ustica)<br>**Shege** (PA-Contessa Entellina)<br>**Ranatu** (TP-Favignana, Mazara del Vallo, Riserva dello Zingaro)
Raphanus raphanistrum L.<br>(*Brassicaceae*)	**Razza** (CT-Nicolosi, S. Giovanni la Punta, Pedara, S. Venerina, Zafferana, Linguaglossa, Castiglione, Randazzo, Adrano, Bronte, Ragaina, Belpasso, Milo)<br>**Razza ruci** (CT-Maletto)
Raphanus raphanistrum L.subsp.*landra* (Moretti) Bonnier<br>(*Brassicaceae*)	**Alassani** (AG-Cattolica Eraclea)<br>**Amareddi** (EN-Enna, Piazza Armerina)<br>**Mazzaredda** (AG-Aragona, Comitini, Favara)<br>**Razza** (PA-Caccamo)<br>**Spicunedda di lassaru** (AG-Sambuca Di Sicilia)<br>**Vrucculuni**, (AG-Siculiana)
Raphanus raphanistrum L. subsp. *raphanistrum*<br>(*Brassicaceae*)	**Lapistra** (PA-Ganci, Pollina, San Mauro Castelverde)<br>**Razza duci** (CT-Maletto)<br>**Razzi** (ME-Mistretta)<br>**Rraricedda** (RG-Vittoria)<br>**Razza** (CT-Nicolosi, S. Giovanni la Punta, Pedara, S. Venerina, Zafferana, Linguaglossa, Castiglione, Randazzo, Adrano, Bronte, Ragaina, Belpasso, Milo)<br>**Sanapieddu duci** (CL-Butera, Riesi)<br>**Tadduzzu sanapa** (PA-Collesano)<br>**Zarra amara** (TP-Favignana)<br>**Zazzu** (TP-Castelvetrano, Partanna)
Rapistrum rugosum (L.) All.<br>(*Brassicaceae*)	**Mazzareddra** (AG-Licata)<br>**Sinapina** (AG-Vittoria)
Reichardia picroides (L.) Roth.<br>(*Asteraceae*)	**Insalatedda** (AG- Canicattì, Campobello di Licata)<br>**Lattugheddra** (AG-Ravanusa)<br>**Coccialebbra** (AG-Favara)<br>**Caccialebbra** (CT-S.Giovanni la Punta, Zafferana, Adrano)<br>**Caccialebbri** (CT-Pedara , S. Venerina , Milo)<br>**Pirnici, Pirnici duci** (CT-Linguaglossa)<br>**Erva di pinnici** (CT-Nicolosi)<br>**Giallepura** (CT-Maletto, Bronte)<br>**Caccianepura** (CT-Castiglione)<br>**Caccialiepura** (CT-Randazzo)<br>**Caccialebbru** (CT-Adrano)<br>**Caccialepri** (CT-Belpasso)<br>**Scaccialebbra** (CT-Ragaina)<br>**Caccialepre** (EN-Piazza Armerina)<br>**Cattalebra** (PA-Petralia Soprana , Petralia sottana)<br>**Lazzini** (ME-Isole Eolie)

	**Scarcitula** (PA-Ganci)<br>**Cacazzina** (ME-Tortorici)
Ridolfia segetum Moris<br>(*Apiaceae*)	**Finocchiu anitu** (AG-Sant'Angelo Muxaro)
Rosa canina L.<br>(*Rosaceae*)	**Giarrauta** (CT-Bronte)
Rosmarinus officinalis L.<br>(*Lamiaceae*)	**Rosamarina** (ME-Mistretta, Galati Mamertino, Longi, San Marco di Alunzio, Tortorici, Santo Stefano di Camastra, Isole Eolie); (PA-Bisaquino); (AG-Isole Pelagie); (RG-Vittoria)<br>**Rosmarina** (AG-Aragona, Raffadali, Realmonte, Sant'Angelo Muxaro, San Biagio Platani, Bivona, Burgio, Casteltermini, Cianciana, Lucca Sicula, Ravanusa, Montevago)<br>**Rosmarinu** (AG-Comitini, Licata, Siculiana)<br>**Rosmarinu, rusamarinu** (AG-Campobello di Licata, Canicattì, Ravanusa)<br>**Rusamarinu** (AG-Montallegro); (CT-Acireale Catania)
Rubia peregrina L.<br>(*Rubiaceae*)	**Rascalingua** (PA-Petralia Soprana, Petralia Sottana)
Rubus ulmifolius Schott.<br>(*Rosaceae*)	**Rivettu** (AG-Licata)<br>**Rovu** (AG-Montallegro, Ravanusa); (CT-Catania, Acireale,)<br>**Ruetto** (AG-Raffadali)<br>**Ruettu** (AG-Raffadali, Sant'Angelo Muxaro)<br>**Ruitti** (PA-San Mauro Castelverde)<br>**Runzi** (PA-Castronovo); (TP-Trapani, Calatafimi, Campobello Di Mazara, Castelvetrano, Salemi, San Vito Lo Capo, Valderice)<br>**Ruvettu** (AG-Aragona, Cattolica Eraclea, Comitini, Favara, Siculiana, Burgio,<br>Lucca Sicula, Ravanusa, Montevago); (PA-Borghetto, Lercara Friddi, Partinico, Ustica); (TP- Partanna, Poggio Reale, Salaparuta); (ME-Tortorici, San Marco d'Alunzio, Isole Eolie)<br>**Ruviettu** (AG-Bivona, Casteltermini, Cianciana, Canicattì, Montallegro); (CT-Catania, Acireale); (ME-Galati Mamertino, Mistretta), (AG-Canicattì), (RG-Vittoria)<br>**Ruvitti** (PA-Ganci)
Rumex crispus L.<br>(*Polygonaceae*)	**Apazzu** (PA-Lercara Friddi)<br>**Auru acitu** (CL-Butera , Riesi)<br>**Lapazzu** (AG-Aragona, Butera, Comitini, Siculiana)
Rumex scutatus L.<br>(*Polygonaceae*)	**Acìtula** (CT-Nicolosi),<br>**Acitura** (CT-Randazzo)<br>**Ocitura** (CT-Maletto)<br>**Citulidda** (CT-Linguaglossa)
Rumex thyrsoides Desf.<br>(*Polygonaceae*)	**Acitura** (CT-Randazzo, Maletto)
Ruscus aculeatus L.<br>(*Liliaceae*)	**Bammuschitta** (CT-Randazzo)<br>**Bammuscittu** (CT-Maletto, Bronte, Mistretta)<br>**Buscu, Puci pulci, Spina scuparina** (TP-Riserva dello Zingaro)<br>**Grattacula** (PA-Chiusa Sclafani)<br>**Grattaculo** (EN-Piazza Armerina)<br>**Scuparini** (ME-Mistretta)<br>**Spinapruci** (CT-Bronte); (TP-Marettimo, Riserva dello Zingaro)<br>**Spinapurci** (CT-Randazzo)<br>**Sineddu** (PA-Grisi)<br>**Sparabusch** (ME-San Fratello)<br>**Sparaceddi, Sparaci, Sparaci trona servaggi** (TP-Marittimo, Trapani, Erice, Custonaci)<br>**Sparaci di scupazzu** (PA-Ganci)<br>**Sparaci scuparini** (PA-Caccamo, Castelbuono, Piana degli Albanesi, Pollina)<br>**Sparaciu di vadduna** (CT-Zafferana)

	**Sparaciu sarvaggiu** (PA-Petralia Soprana, Petralia Sottana)<br>**Sparaciu 'mpiriali** (CT-S. Giovanni la Punta)<br>**Sparacogna** (CT-Castiglione, Nicolosi, Pedara)<br>**Spinapruci** (ME-Mistretta)<br>**Spinapulci**, **Sparacogna** (CT-Nicolosi, Pedara)<br>**Spinapulici** (ME-Mistretta, Naso); (CT-Castiglione, S. Giovanni la Punta, S. Venerina, Zafferana, Adrano, Ragaina)<br>**Spinocciuoli** (ME-Tortorici)<br>**Taddispruni** (CT-Linguaglossa)<br>**Taddispruni** (ME-Mistretta)
Ruscus hypoglossum L.<br>(*Liliaceae*)	**Sparaci di tronu** (AG-Aragona)<br>**Aparaciu** (AG- Montallegro); (CT-Catania , Acireale)
Ruscus hypophyllum L.<br>(*Liliaceae*)	**Sparaciu 'mpriacu** (CT-Adrano)<br>**Sparaciu 'mpiriali** (CT-Adrano, Linguaglossa)<br>**Sparaciu di Spagna** (CT-Castiglione)<br>**Sparaciu di salamunia** (CT-Milo, S. Venerina)<br>**Sparaciu di Bordura** (CT-San Giovanni la Punta)
Salvia officinalis L.<br>(*Lamiaceae*)	**Salvia** (AG-Campobello di Licata, Canicattì, Ravanusa Montevago, Sambuca di Sicilia); (CL-Caltanissetta, Delia); (TP- Trapani, Erice, Valderice, Custonaci, Castelvetrano, Favignana, Partanna, Poggio Reale Salaparuta); (PA-Partitico, Piana degli Albanesi, Ustica); (RG-Vittoria)<br>**Sarvia** (AG-Realmonte, Aragona, San Biagio Platani); (ME-Mistretta, Isole Eolie); (PA-Bisaquino, Castronovo, Corleone, Roccamena, San Giuseppe Jato); (RG- Vittoria)
Salvia sclarea L.<br>(*Lamiaceae*)	**Erva muscatiddara** (PA-Caccamo, Castellana Sicula, Geraci Siculo, Prizzi)<br>**Muscaiddara** (CL-Mussomeli)<br>**Salvia** (SR-Noto)
Sambucus nigra L.<br>(*Caprifoliaceae*)	**Savuiccu, Savucu** (CL-Mussomeli); (PA-Castellana Sicula)<br>**Sammuccu, Savucu** (PA-Bisaquino)
Scolymus grandiflorus Desf.<br>(*Asteraceae*)	**Pepellu** (CL-Butera, Mazzarino, Riesi, Sommatino)<br>**Rattameli** (CT-Linguaglossa)<br>**Scoddi** (Adrano, Biancavilla, Regaina, Belpasso)<br>**Scoddi** (AG-Favara, Siculiana Bivona, Casteltermini, Cianciana, Canicattì)<br>**Scoddu** (CT-Giarre)<br>**Scolli** (CT-Randazzo , Maletto, Bronte)<br>**Scuoddi** (ME-Mistretta)<br>**Scuoddu, Scuedda, Scoddi** (PA-Caccamo, Collesano, Ganci, San Giuseppe Jato, San Mauro Castelverde)<br>**Scuddu, Scoddo, Scoddi** (TP-Buseto Palizzolo, Castellammare del Golfo, Castelvetrano, Custonaci, Levanzo, Favignana, Marettimo, Partanna, Poggio Reale, Salaparuta, San Vito lo Capo, Valderice, Vita, Riserva dello Zingaro)<br>**Zammuri di campagna** (CT-Zafferana)
Scolymus hispanicus L.<br>(*Asteraceae*)	**Scoddu** (PA-Vicari)<br>**Spina bianca** (AG-Sant'Angelo Muxaro)
Scolymus maculatus L.<br>(*Asteraceae*)	**Scoddu** (AG-Cattolica Eraclea, Sant'Angelo Muxaro, San Biagio Platani)<br>**Scuoddo** (CL-Butera, Riesi)<br>**Scuoddu** (PA-Valledolmo, Collesano)
Silene vulgaris (Moench.) Garcke<br>(*Caryophyllaceae*)	**Calicedda di muru** (CT-Biancavilla)<br>**Calicedda î mura** (CT-Adrano)<br>**Cannatella** (CT-Randazzo)<br>**Ebba priricatura** (CT-Bronte)<br>**Cannatedda** (CT-Nicolosi, S. Giovanni la Punta, S. Venerina, Zafferana, Milo, Linguaglossa, Ragaina, Belpasso)<br>**Erba du pridicaturi** (CT-Randazzo)<br>**Erba pridicatura** (CT-Castiglione)<br>**Campanedda** (CT-Pedara, Zafferana)

	**Erva ru priricaturi** (CT-Maletto)<br>**Priricaturi** (CT-Randazzo)
Silene vulgaris (Moench.) Garcke subsp. *angustifolia* (Miller) Hayek (*Caryophyllaceae*)	**Calicedda di muru** (CT-Biancavilla)<br>**Calicedda i mura** (CT-Adrano)<br>**Campanedda** (CT-Pedara Zafferana)<br>**Cannatedda** (CT-Nicolosi, S. Giovanni la Punta, S. Venerina, Zafferana, Milo, Linguaglossa, Ragaina, Belpasso)<br>**Cannatella, Erba du priricaturi, Pricaturi** (CT-Randazzo)<br>**Ebba priricatura** (CT-Bronte)<br>**Erba pridicatura** (CT-Castiglione)<br>**Erva ru priricaturi** (CT-Maletto)<br>**Scaf'ti'nfrunti** (EN-Piazza Armerina)
Silybum marianum L. (*Asteraceae*)	**Cardu marianu** (TP-Riserva dello Zingaro)<br>**Cardunazzu** (TP-Zingaro, Canicatti)<br>**Cardunciu** (PA-Ganci)<br>**Crox** (PA-San Mauro Castelverde),<br>**Mianu** (TP-Zingaro)<br>**Piscia l'asinu** (AG-Campobello di Licata)
Sinapis alba L. (*Brassicaceae*)	**Lassani** (AG-Bivona)<br>**Mazzareddu, Arazzi** (AG-Canicattì)
Sinapis arvensis L. (*Brassicaceae*)	**Alassani** (AG-Raffadali)<br>**Qualeddu** (TP-Buseto Palizzolo, Campobello di Mazara, Custonaci, Erice, Valderice)
Sisymbrium officinale (L) Scop. (*Brassicaceae*)	**Lassani duci** (TP-Zingaro)<br>**Lassinu di sceccu** (TP-Zingaro)<br>**Lssaneddi** (TP-Zingaro)<br>**Mazzareddri** (ME-Mistretta)<br>**Sciuriti, Ciunciuli** (PA-Ganci)
Sisymbrium irio L. (*Brassicaceae*)	**Aprocchiu** (AG-Favara)
Smilax aspera L. (*Smilacaceae*)	**Cafaretra** (ME-Santo Stefano di Camastra)<br>**Cannacìtura** (CT-Castiglione)<br>**Gratta culu, Ratta culu, Liara, Sarsa siciliana, Sciracausi, Erva serretta** (TP-Zingaro)<br>**Raja** (CT-Nicolosi, S. Giovanni la Punta, Pedara, S. Venerina, Linguaglossa, Randazzo, Ragaina. Belpasso)<br>**Strazzacammisi** (CT-Maletto, Bronte)<br>**Ugna di attu** (CT-Adrano)<br>**Ugna ri gattu** (CT-Bronte)
Smyrnium olusatrum L. (*Apiaceae*)	**Accia sarvaggia** (AG-Lucca Sicula)<br>**Laccia sarvaggia** (ME-Salina)
Sonchus asper L. (*Asteraceae*)	**Cardedda** (ME-Mistretta); (CT-Linguaglossa, Belpasso, Milo, Pedara, Zafferana)<br>**Cardedda mascula** (CT-Adrano)<br>**Cardedda masculina** (AG-Sambuca Di Sicilia, Sant'Angelo Muxaro); (CT- Castiglione, Ragaina, San Giovanni La Punta, Santa Venerina<br>**Cardedda di cani** (AG-Favara)<br>**Cardedda di puorci** (CL-Mussomeli)<br>**Cardedda spinusa** (CT-Nicolosi , Maletto, Bronte)<br>**Cardedda ri porci** (CT-Bronte)<br>**Cardedda niura** (CT-Nicolosi)<br>**Cardedda russa** (CT-Randazzo)
Sonchus asper (L.) Hill subsp.*nymanii* (Tineo et Guss.) Hegi (*Asteraceae*)	**Cardedda (**Collesano**)**
Sonchus oleraceus L. (*Asteraceae*)	**Cardedda** (AG-Cattolica Eraclea, Licata, Raffadali); (PA-Borgetto, Camporeale, Castelbuono, Geraci Siculo, Grisi, Lercara Friddi,

	Palazzo Adriano, Petralia Soprana, Pollina, Prizzi, San Mauro Castelverde, Vicari, Ustica); (TP-Partanna); (CT-Belpasso, Bronte , Linguaglossa, Milo, Pedara, Zafferana); (ME-Isole Eolie, Mistretta); (RG-Vittoria)<br>**Cardedda bianca** (PA-Petralia Soprana, Petralia Sottana); (CT-Randazzo)<br>**Cardedda femminina** (AG-Sambuca Di Sicilia<br>**Cardedda fimmina** (CT-Adrano)<br>**Cardedda fimminedda** (CT-Castiglione, Ragaina, San Giovanni La Punta, Santa Venerina)<br>**Cardedda janca** (CT-Maletto)<br>**Cardedda liscia** (CT-Nicolosi, Maletto)<br>**Cardedda scucivola** (AG-Aragona, Realmonte)<br>**Cardella** (AG-Isole Pelagie)<br>**Cardetra** (AG-Sant'Angelo Muxaro, San Biagio Platani)<br>**Scarola** (AG-Canicattì)
Sonchus tenerrimus L.<br>(*Asteraceae*)	**Cardedda** (ME-Isole Eolie); (PA-Caccamo, Castellana Sicula, Corleone, Ganci, San Giuseppe Jato, San Mauro Castelverde, Ustica); (TP-Levanzo, Marettimo, Favignana)<br>**Cardedda femminina** (CL-Campofranco, Sutera)<br>**Cardedda scucivola, Cardeddra** (CL-Caltanissetta, San Cataldo, Serradifalco)<br>**Cardella** (AG-Isole Pelagie)<br>**Kardedda** (RG-Vittoria)<br>**Spargola** (CL-Butera, Riesi); (AG-Realmonte, Siculiana)
Sorbus domestica L.<br>(*Rosaceae*)	**Zorba** (AG-Sambuca di Sicilia); (Pa-Partinico)
Tamus communis L.<br>(*Dioscoreaceae*)	**Sparaciu arrampicusu** (PA-Castelbuono)<br>**Sparaci cuccu** (PA-Caccamo)<br>**Sparaci cuccu, Sparaci di serpa, Sparaci i' serpi**<br>**Sparaci di serpa** (PA-Chiusa Sclafani)<br>**Sparaci i serpi** (PA-Ganci , San Mauro Castelverde)<br>**Sparaciu 'mpiariali** (CT-Milo,<br>**Sparaciu impiriali** CT-Ragaina)<br>**Sparaciu di lupu** (AG-Burgio, Lucca Sicula, Ravanusa)<br>**Sparaciu di serpa** (AG-Cammarata, Cianciana)<br>**Sparaciu di tronu** (PA-Bisaquino)<br>**Sparacogni** (CT-Maletto , Bronte, Adrano, Biancavilla)<br>**Sparacognu** (CT-Maletto)<br>**Sparacuogna** (CT-Randazzo)<br>**Tamusceddi** (CT-Piazza Armerina)<br>**Viddiceddu** (CT-Castiglione)<br>**Virriceddu** (CT-Castiglione)<br>**Viticeddu** (CT-Castiglione)
Tanacetum vulgare L.<br>(*Asteraceae*)	**Tannavira** (ME-Tortorici)
Taraxacum officinale Weber<br>(*Asteraceae*)	**Tarassacu, Erba di pirnici, Piscialetto** (CL-Caltanissetta, San Cataldo, Serradifalco)
Teucrium fruticans L.<br>(*Lamiaceae*)	**Ricutedda** (CL-Butera)
Teucrium scordium L.<br>(*Lamiaceae*)	**Scordiu** (PA-Bisaquino)
Thymus capitatus (L.) Hoffmanns. et Link<br>(*Lamiaceae*)	**Santariedda** (RG-Vittoria)<br>**Santarieddru** (CL-Campofranco, Sutera)<br>**Sataredda** (AG-Cattolica Eraclea, Sambuca di Sicilia, San Biagio Platani, Siciliana, Raffadali)<br>**Satareddu** (TP-Castelvetrano, Favignana, Partanna, Poggio Reale, Salaparuta)<br>**Sitaredda** (AG-Isole Pelagie)<br>**Timu** (EN-Piazza Armerina), (TP-Erice)
Thymus spinulosus Ten.	**Timo**

(*Lamiaceae*)	
Tolpis virgata (Desf.) Bertol.<br>(*Asteraceae*)	**Erba janca** (CT-Linguaglossa)<br>**Gallinella** (CT-Maletto)<br>**Lattuchedda** (CT-Nicolosi)<br>**Scaluredda** (CT-Belpasso, Zafferana)
Tragopogon crocifolius L.<br>(*Asteraceae*)	**Barbabecchi** (CT-Randazzo)<br>**Brambascu** (CT-Linguaglossa)<br>**Cuttuneddu** (CT-Nicolosi)<br>**Erva di S. Petru** (CT-Pedara)<br>**Lattaroli** (CT-Maletto)<br>**Pampasciuscia** (CT-Nicolosi)<br>**Latti d'aceddu** (CT-S. Giovanni la Punta , Zafferana, Milo)<br>**Pedi di lupu** (Castiglione)<br>**Percia canedda** (AG-Sant'Angelo Muxaro)<br>**Pistalaceddi** (CT-Zafferana)<br>**Stuppacannedda** (CT-Pedara)
Tragopogon porrifolius L. subsp. *australis* (Jordan) Br.-Bl.<br>(*Asteraceae*)	**Erba di gnagnaru pilusa** (CL-Caltanissetta)<br>**Pedi di gaddru** (CL-Butera, Mazzarino, Riesi, Sommatino)<br>**Perciacanneda** (PA-Lercara Friddi , Mussomeli)<br>**Reggia** (EN-Piazza Armerina)<br>**Varva di beccu** (CL-Caltanissetta)
Urospermum dalechampii L.Schmidt<br>(*Asteraceae*)	**Cartedda, Cicoria sarvaggia, Scursunera giara** (PA-Palermo, Chiusa Sclafani, Collesano, Lercara Friddi, Palazzo Adriano, Prizzi, Vicari)<br>**Cuosti i porci** (ME-Mistretta)<br>**Cicoina** (ME-Tortorici, Santo Stefano di Camastra)
Urospermum picroides L.Schmitd.<br>(*Asteraceae*)	**Coccialebbra** (AG-Sant'Angelo Muxaro<br>**Bukeriepur** (PA-Piana Degli Albanesi)
Urtica membranacea Poiret<br>(*Urticaceae*)	**Addricula** (AG-Bivona)<br>**Ardicela** (PA-Bisaquino), (AG-Aragona, Realmonte, Montevago)<br>**Ardichi** (PA-Castelbuono)<br>**Lurida** (PA-Polizzi Generosa), (AG-Favara, San Biagio Platani)<br>**Ziculiedda** (AG-Sant'Angelo Muxaro)
Urtica urens L.<br>(*Urticaceae*)	**Arzicula, ardica** (PA-Ustica)
Valerianella eriocarpa Desf.<br>(*Valerianaceae*)	**Gaddinedda , periciocca, lattughedda** (TP-Riserva dello Zingaro)
Veronica anagallis-aquatica L.<br>(*Scrophulariaceae*)	**Crisciuni, scavuni** (CL-Caltanissetta)
Vitis vinifera L.<br>(*Vitaceae*)	**Zuccu, Racina** (TP-Erice, Custonaci, Valderice)<br>**Racina,Vigna,Vigni,**<br>**Viti** (TP-Riserva dello Zingaro)<br>**Racina, Vigna, Viti Zuccu** (TP-Mazara del Vallo)

The names reported in brackets refer to the villages where a food ethnobotanical use was registered; the code to the provinces where the villages belong to:
AG-Agrigento; CL-Caltanisetta; CT-Catania; EN-Enna; ME-Messina, PA-Palermo; RG-Ragusa; SR-Siracusa; TP-Trapani (The Egadi Islands (Favignana, Levanzo and Marittimo) and the island of Pantelleria are part of the Trapani province; the Eolian Islands (Alicudi, Filicudi, Lipari, Panarea, Salina, Stromboli and Vulcano) are part of the Messina province; the Pelagie Islands (Lampedusa and Linosa) are part of the Agrigento province; the Island of Ustica is included in the Palermo province)
